# Parental Mental Health and Suicidal Behavior as Predictors of Adolescent Suicidal Ideation and Attempts: A Systematic Review and Meta-Analysis

**DOI:** 10.3390/jcm14196860

**Published:** 2025-09-28

**Authors:** Elena Alexandra Bratu, Lavinia-Alexandra Moroianu, Oana-Maria Isailă, Cătălin Pleșea-Condratovici, Oana-Elisabeta Avram, Eduard Drima

**Affiliations:** 1 Doctoral School of Biomedical Sciences, Dunărea de Jos University, 800201 Galati, Romania; alexandra.brt99@gmail.com (E.A.B.); titeoana@yahoo.com (O.-E.A.); 2 Department of Pharmaceutical Sciences, Dunărea de Jos University, 800201 Galati, Romania; lavinia.moroianu@yahoo.com; 3 Department of Legal Medicine and Bioethics, Faculty of Dentistry, “Carol Davila University” of Medicine and Pharmacy, 020021 Bucharest, Romania; 4 Morphological and Functional Sciences Department, Faculty of Medicine and Pharmacy, “Dunărea de Jos” University, 800201 Galati, Romania; 5 Clinical Medical Department, Faculty of Medicine and Pharmacy, “Dunărea de Jos” University, 800201 Galati, Romania; drima_edi1963@yahoo.com

**Keywords:** adolescent suicidality, intergenerational risk, meta-analysis, parental mental health, suicidal behavior, systematic review

## Abstract

**Background/Objectives**: The intergenerational transmission of suicidal risk is a major global health concern. Evidence on the role of parental psychopathology, including suicidal behavior, in predicting adolescent suicidality remains inconsistent. This systematic review and meta-analysis aimed to synthesize recent findings and quantify these associations. **Methods**: The review followed the Preferred Reporting Items for Systematic Reviews and Meta-Analyses (PRISMA) guidelines and the Population, Intervention/Exposure, Comparator, Outcome (PICO) framework. Searches (2015–2025) identified observational studies on parental suicidal behavior, depression, or psychiatric disorders predicting adolescent suicidal ideation or attempts. Thirty-one studies met eligibility, including over 12 million adolescents. Random-effects meta-analyses estimated pooled associations. Study quality was rated with the Newcastle–Ottawa Scale (NOS), and evidence certainty with the Grading of Recommendations, Assessment, Development and Evaluation (GRADE). **Results**: Both parental suicidal behavior and psychiatric disorders were consistently linked to increased adolescent suicidality. The pooled odds ratio (OR) for any parental psychopathology was 2.77 (95% confidence interval, CI: 2.22–3.47), indicating nearly a threefold higher risk of suicidal ideation or attempts in exposed youth. Subgroup analyses showed comparable risks for parental suicidal behavior (OR = 2.69, 95% CI: 2.30–3.14) and parental psychiatric morbidity (OR = 2.72, 95% CI: 2.05–3.60). **Conclusions**: Parental psychopathology, whether manifesting as suicidal behavior or psychiatric disorder, is a strong and universal risk factor for adolescent suicidal ideation and attempts. These findings underscore the need for family-centered prevention, early identification, and targeted interventions to disrupt intergenerational transmission of suicide risk.

## 1. Introduction

### 1.1. Central Research Question

Adolescent suicide constitutes a grave and escalating public health crisis worldwide, cutting across geographical, cultural, and socioeconomic boundaries [1,2,3,4]. In this review, we explicitly address both suicidal ideation and suicidal attempts, as these outcomes are conceptually distinct yet closely interrelated. According to the Diagnostic and Statistical Manual of Mental Disorders, Fifth Edition (DSM-5) and the International Classification of Diseases, Tenth Revision (ICD-10), suicidal ideation refers to thoughts of engaging in behavior intended to end one’s life, whereas a suicidal attempt denotes a non-fatal, self-directed, potentially injurious act carried out with at least some intent to die [5,6,7,8]. Both outcomes represent critical indicators of suicide risk, with suicidal ideation often preceding attempts, and attempts being among the strongest predictors of eventual suicide death.

Mounting evidence has established that suicidal ideation and behaviors during adolescence are not isolated phenomena but frequently arise from a convergence of individual vulnerabilities and familial risk factors, among which parental mental health and suicidal behavior figure prominently. The intergenerational transmission of risk is now recognized as multidimensional, involving genetic, psychological, and environmental pathways. Nevertheless, significant uncertainty persists regarding the comparative magnitude of risk conferred by parental suicidal behavior as opposed to broader parental psychopathology, as well as the extent to which these associations are robust across diverse populations and methodological approaches [9,10,11].

Over the last decade, the literature on the familial transmission of suicidality has expanded considerably in scope and rigor. Large-scale cohort studies, national registry linkages, cross-sectional surveys, and genetically informed designs have collectively elucidated the consistent association between parental mental health problems—including suicidal behavior, depression, bipolar disorder, and substance use disorders—and increased risk of suicidal ideation and attempts in adolescent offspring. Notably, these associations remain significant after accounting for a range of confounders and mediators, such as socioeconomic status, offspring psychiatric morbidity, and exposure to adversity. While both genetic vulnerability and environmental mechanisms—such as impaired parenting, family disruption, and modeling of maladaptive coping—contribute to this risk, the relative strength and specificity of these effects across distinct parental exposures has not been conclusively determined [12,13,14,15,16,17,18,19,20].

Despite this rapidly maturing evidence base, previous reviews have been limited by narrow inclusion criteria, insufficient attention to study heterogeneity, or lack of systematic meta-analytic synthesis [21,22,23,24,25,26,27,28,29,30]. To address these gaps, the present systematic review and meta-analysis synthesizes data from 31 observational studies published between 2015 and 2025, encompassing over 12 million participants, including both parents and their adolescent offspring, from six continents [31,32,33,34,35,36,37,38,39,40,41,42,43,44,45,46,47,48,49,50,51,52,53,54,55,56,57,58,59,60,61]. Our analytic framework is unique in its direct comparison of two principal parental exposures—suicidal behavior (including suicide attempts and deaths) and psychiatric disorders (including depression, anxiety, bipolar disorder, and substance use)—and their impact on two major adolescent outcomes: suicidal ideation and suicide attempts. The robustness of the findings is underscored by rigorous random-effects modeling, cumulative and sensitivity analyses, and explicit assessment of publication bias and study quality.

Accordingly, this review addresses the following hypotheses: Null Hypothesis (H_0_): There is no statistically significant association between parental suicidal behavior or psychiatric disorders and the risk of suicidal ideation or suicide attempt in adolescent offspring. Alternative Hypothesis (H_1_): Both parental suicidal behavior and parental psychiatric disorders are independently and significantly associated with an increased risk of suicidal ideation and suicide attempts among adolescents, with comparable effect sizes across exposure types, as demonstrated by pooled estimates from recent observational studies.

### 1.2. Theoretical Background and Scientific Rationale

#### 1.2.1. Methodological Issues and Gaps in the Literature

Research on the association between parental mental health—including suicidal behavior—and adolescent suicidality has expanded considerably, yet significant methodological challenges remain. A key limitation is the heterogeneity in how exposures and outcomes are defined and measured. Some studies rely on self-report or administrative data, while others use standardized diagnostic tools, making comparisons across contexts difficult. For instance, certain analyses aggregate multiple parental disorders or conflate suicidal ideation with attempts, whereas others draw finer distinctions, leading to variability in reported effect sizes.

Study design is another challenge. Large registry-based cohort studies have advanced the field, but many investigations remain cross-sectional, limiting causal inference. Longitudinal studies, which are essential for clarifying timing and pathways of intergenerational transmission, are relatively scarce. Furthermore, few studies consistently adjust for key confounders such as socioeconomic adversity, family structure, or adolescent psychopathology, raising concerns about whether observed associations reflect direct parental effects, shared environments, or unmeasured variables.

Generalizability also remains limited. Earlier research predominantly originated from Western, high-income countries, with less representation from diverse cultural and socioeconomic settings. More recent contributions from Asia, Africa, and Latin America broaden the evidence base, but data from these regions remain relatively sparse. Given rapid changes in mental health systems and family dynamics, synthesizing contemporary studies—particularly from the last decade—is crucial for ensuring relevance to current clinical and policy contexts.

#### 1.2.2. Conceptual Basis and Rationale for the Review

Adolescent suicidal thoughts and behaviors rarely occur in isolation; rather, they reflect the interaction of biological, psychological, and social influences. Stress–diathesis models suggest that parental mental illness may function as both genetic vulnerability and a chronic stressor, heightening maladaptive coping in adolescents [62,63,64,65,66]. Attachment theory and related frameworks further emphasize the role of parental mental health in shaping relationship quality, emotional security, and coping development, which are consistently linked to resilience or vulnerability [67,68,69,70].

Recent evidence indicates that risk is not only genetically transmitted but also influenced by environmental mechanisms such as impaired parenting, family conflict, and the modeling of suicidal behavior [71,72,73]. Although the association between parental psychopathology and adolescent suicidality appears robust across varied populations, its magnitude may depend on the type and chronicity of parental disorder, the developmental stage of exposure, and moderating factors such as offspring sex or age. This underscores the need for an updated, systematic synthesis that clarifies when and under what conditions risk is most pronounced.

By integrating recent high-quality studies and applying rigorous meta-analytic methods, this review addresses central questions in the field: How strong is the association between parental suicidal behavior or mental illness and adolescent suicidal ideation or attempts? Are these risks consistent across cultures and study designs? And what methodological limitations shape the certainty of current knowledge? Ultimately, the synthesis aims to guide future research and inform family-centered prevention strategies, offering timely, evidence-based insights for clinical and public health practice.

### 1.3. Research Objectives and Conceptual Methodology

The primary objective of this review is to quantify the association between parental mental health problems—including suicidal behavior and psychiatric disorders—and adolescent suicidal ideation or attempts. Recognizing the persistent global burden of adolescent suicidality, this study clarifies the magnitude, consistency, and mechanisms of intergenerational risk transmission. It focuses on contemporary evidence (2015–2025), spanning diverse populations and applying rigorous standards to enhance comparability. The review was conducted according to PRISMA guidelines for systematic reviews and meta-analyses [74]. Comprehensive searches across multiple databases identified observational studies (prospective cohorts, cross-sectional, case–control, and registry-based) assessing parental exposures (suicide attempt, suicide death, depression, bipolar disorder, substance use) and adolescent outcomes (suicidal ideation or attempt). Inclusion was limited to English-language studies with human participants from childhood to late adolescence and quantitative estimates (e.g., odds ratios, hazard ratios) suitable for meta-analysis.

A dual-reviewer process ensured reliability in study selection, data extraction, and bias assessment. Quality was rated with standardized tools, and information on sample characteristics, exposures, outcomes, covariates, and analytic methods was systematically recorded. Data were stratified by design, exposure type, and outcomes to facilitate subgroup and sensitivity analyses. Random-effects models with inverse-variance weighting and restricted maximum likelihood estimation were applied. Heterogeneity was examined using I^2^, τ^2^, and Q statistics, with subgroup and meta-regression analyses conducted as appropriate. Publication bias was assessed with funnel plots and Egger’s test, and overall certainty was graded using adapted GRADE criteria [75,76].

Several included studies applied advanced designs—genetically informed cohorts, mediation models, or quasi-experimental approaches—allowing more precise separation of direct parental influences from genetic and environmental effects. The review also integrates findings on potential mediators and moderators (e.g., adverse childhood experiences, parent–child relationship quality, offspring psychopathology), deepening understanding of transmission pathways and opportunities for mitigation. Finally, emerging applications of artificial intelligence (AI) and machine learning in evidence synthesis were considered [77,78,79,80,81,82,83,84,85,86,87,88,89,90,91,92,93]. These approaches may enhance large-scale data integration and detection of complex risk patterns, though they remain complementary to rigorous epidemiological methods. By combining robust methodologies with nuanced conceptual framing, this review aims to provide actionable evidence for researchers, clinicians, and policymakers seeking to reduce adolescent suicidality through family-centered and innovative prevention strategies.

## 2. Materials and Methods

### 2.1. Methodological Protocol and Study Registration

This systematic review and meta-analysis was conducted in accordance with internationally recognized standards for evidence synthesis. The protocol was developed a priori and guided all stages of the review, including eligibility criteria, search strategy, study selection, data extraction, and analysis. Methodological decisions followed best practices outlined in the PRISMA statement and the PICO framework [94,95].

Although prospectively designed, the protocol was not registered in PROSPERO (International Prospective Register of Systematic Reviews) [96] or another registry. Instead, it was documented internally and consistently applied. All inclusion/exclusion criteria, search strategies, analytic procedures, and bias assessments were predefined prior to data extraction and adhered to throughout. This ensured transparency, reproducibility, and alignment with current standards for systematic reviews and meta-analyses.

### 2.2. Search Architecture and Comprehensive Source Mapping

A comprehensive search strategy was designed to capture studies on parental mental health, parental suicidal behavior, and adolescent suicidality. In line with PRISMA guidelines and the PICO framework, searches were conducted across PubMed/MEDLINE, ClinicalTrials.gov, the Cochrane Library (CENTRAL), and ProQuest Dissertations & Theses Global. These platforms were selected for complementary coverage of peer-reviewed, registry, and gray literature.

For PubMed/MEDLINE, the search strategy combined MeSH terms and free-text keywords covering parental psychiatric disorders (e.g., depression, anxiety, bipolar disorder, substance use, schizophrenia), suicidal behavior, and adolescent suicidal outcomes (ideation, attempts, self-harm, suicide-related behavior). Limits included observational and population-based studies published between January 2015 and March 2025, in humans, and in English. ClinicalTrials.gov was queried using advanced searches targeting parental mental health and adolescent suicidality, restricted to observational studies initiated or completed between 2015 and 2025. CENTRAL was searched with MeSH and keywords similar to PubMed, with additional screening for study design and quality. ProQuest Dissertations & Theses Global was searched to capture unpublished or gray literature, filtered by English language, document type, and publication year (2015–2025).

The full matrix of queries, terms, filters, and field restrictions is presented in Supplementary Materials (Table S1). All records were imported into Mendeley Reference Manager (v.2.135.0) for bibliographic management, deduplication, and tracking through screening and eligibility. This integrated multi-platform search ensured sensitivity and specificity, minimized risk of publication or language bias, and enabled inclusion of both published and unpublished research across diverse contexts.

### 2.3. Eligibility Criteria, Study Selection and Data Extraction Framework

Eligibility criteria were defined a priori using PRISMA and structured via the PICO framework to ensure transparency and methodological consistency. Studies were eligible if they: (a) reported original, peer-reviewed observational research (prospective/retrospective cohort, cross-sectional, case–control, or registry-based); (b) were published in English between January 2015 and March 2025; (c) directly examined the association between parental mental health—including suicidal behavior, depression, bipolar disorder, anxiety, substance use disorders, or broader psychiatric morbidity (e.g., psychotic or personality disorders)—and adolescent suicidal ideation or suicide attempts; (d) provided quantitative effect measures (odds ratios, hazard ratios, relative risks, or equivalents with confidence intervals) suitable for meta-analysis; and (e) included adolescent populations defined per the World Health Organization as 10–19 years [97], with wider youth ranges considered if disaggregable or directly relevant.

**Figure 1:**
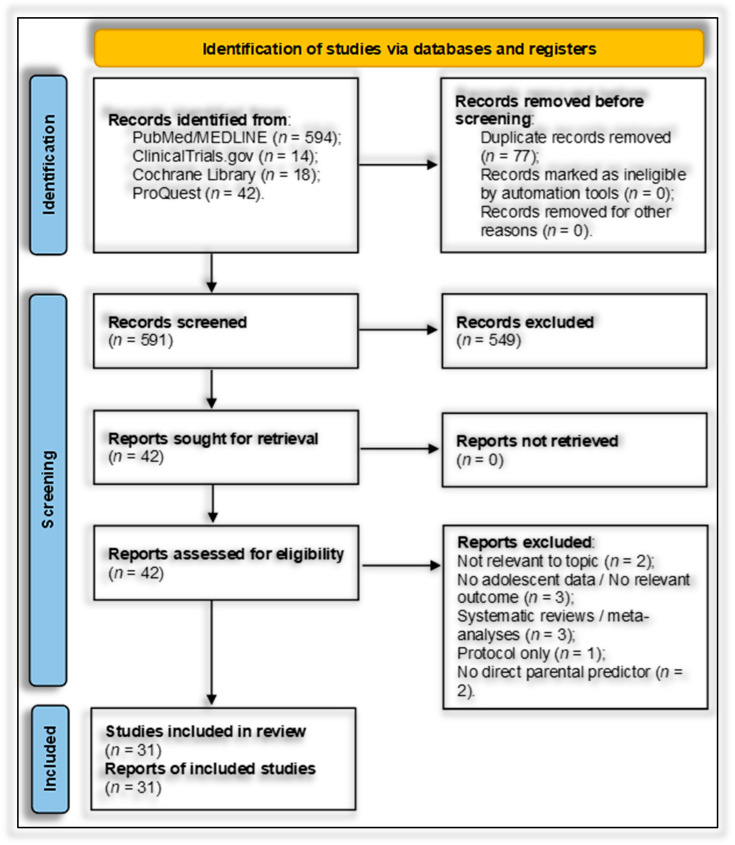
PRISMA 2020 Flowchart Illustrating Study Identification, Screening Procedures, and Inclusion Pathway.

Exclusion criteria were: studies of non-parental predictors (e.g., sibling, peer, or environmental-only exposures), lack of adolescent suicidality outcomes, non-original quantitative reports (systematic reviews, meta-analyses, narrative reviews, protocols, editorials), missing extractable effect sizes or insufficient statistics, non-human studies, and non-English publications. When multiple reports derived from the same cohort, the most comprehensive, methodologically robust report was retained to avoid duplication. The selection process is summarized in Figure 1.

Systematic searches of PubMed/MEDLINE (*n* = 594), ClinicalTrials.gov (*n* = 14), the Cochrane Library (*n* = 18), and ProQuest Dissertations & Theses Global (*n* = 42) yielded 668 records. After import into Mendeley (v. 2.135.0) and automated/manual deduplication, 77 duplicates were removed, leaving 591 unique records. Two independent reviewers screened titles/abstracts in Rayyan (Rayyan for Systematic Reviews) with blinded, piloted procedures [98,99]; disagreements were resolved by discussion or third-reviewer adjudication. Forty-two full texts were assessed; 11 were excluded (2 not relevant to the research question, 3 without adolescent suicidality outcomes, 3 systematic reviews/meta-analyses, 1 protocol without original results, 2 without a direct parental predictor) [100,101,102,103,104,105,106,107,108,109,110]. In total, 31 studies met all criteria and were included in the narrative synthesis and meta-analysis. No studies were excluded due to inaccessible full text (Supplementary Materials, Table S3).

Data extraction used a dual-reviewer, pre-piloted form aligned with review objectives (Supplementary Materials, Table S2). For each included study, we recorded: design (prospective cohort, registry-based, cross-sectional, case–control), country/setting, sample size, data-collection years, adolescent characteristics/age range, parental exposures (specific diagnoses, suicidal behaviors, composite indicators), operationalization/measurement of exposures and outcomes, covariates/confounders, analytic strategy (e.g., regression model, adjustments, mediation), effect-size metrics (OR, HR, IRR with 95% CIs or SEs), follow-up duration (if applicable), and sex/subgroup results. Special attention was given to precise ascertainment of parental psychopathology (e.g., lifetime clinical diagnosis, recent symptoms, registry codes, self-report) and suicidal behavior (attempt, death, ideation), and to adolescent outcomes (recent vs. lifetime ideation/attempts; clinical vs. self-report severity). Each data point was cross-verified; disagreements were resolved by discussion or third-reviewer adjudication, with an audit trail documenting coding decisions. We explicitly captured analytic details critical for meta-analysis (e.g., covariate adjustments, handling of missing data/attrition, and subgroup/sensitivity strata by sex, age, exposure type, or country income level). All data were maintained in a version-controlled master spreadsheet (Excel), with integrity checks; extraction status and documents were tracked in Rayyan, with encrypted storage of forms and PDFs. A summary of variables included in the meta-analysis is provided in Supplementary Materials (Table S4).

### 2.4. Analytical Software Framework and Evidence Quality Evaluation

Bibliographic management and screening were conducted with established platforms to ensure transparency and reproducibility, using reference management software and blinded dual review systems. Quantitative synthesis was performed with Comprehensive Meta-Analysis (CMA v.4, Biostat) and R (v.4.3.1; “meta” and “metafor” packages) [111,112]. These tools enabled calculation of pooled effect sizes, random-effects modeling, assessment of heterogeneity, and evaluation of publication bias. Subgroup, sensitivity, cumulative analyses, and meta-regression were applied where appropriate, with all analytic code archived for reproducibility.

Methodological quality and risk of bias for individual studies were appraised with the Newcastle–Ottawa Scale (NOS), with domain-specific ratings summarized in Supplementary Materials (Table S5). Certainty of evidence across outcomes was assessed using the GRADE framework, considering study limitations, inconsistency, indirectness, imprecision, and publication bias. Final ratings (high, moderate, low, or very low) were independently assigned by two reviewers and reconciled by consensus. This integrated framework ensured that the evidence synthesis was rigorous, transparent, and aligned with international standards for systematic reviews and meta-analyses.

## 3. Results

### 3.1. Qualitative Synthesis and Critical Appraisal of Included Studies

#### 3.1.1. Statistical Overview and Study Design Features

This systematic review synthesized evidence from 31 studies published between 2015 and 2025, collectively encompassing a sample of over 12 million youth (Supplementary Materials, Table S4). The scope of study populations ranged from small, high-risk clinical samples of fewer than 200 adolescents to vast, nationwide cohorts involving over 4 million individuals. Across studies, the age of adolescent participants typically spanned from approximately 6 to 19 years, with most samples achieving a roughly balanced gender distribution—several large-scale investigations reported near-equal proportions of female and male participants [37,51,53]. While one study focused exclusively on adolescent girls [48], most included both sexes and, in some instances, conducted sex-stratified analyses to elucidate potential gender-specific pathways. Geographically, the body of evidence was truly global, encompassing studies from North America (United States), Europe (including the United Kingdom, Ireland, Denmark, Finland, Sweden), Asia (South Korea, Japan, China), Africa (Tanzania, South Africa), Oceania (Australia, New Zealand), and Latin America (Brazil). Study periods varied considerably: some research utilized cross-sectional survey designs conducted at a single time point (often between 2010 and 2018), whereas many were longitudinal in nature, with follow-up durations ranging from one year to decades—such as a Finnish birth cohort followed to age 28, or a U.S. clinical cohort tracked for approximately 14 years [41].

In terms of study design, the review included 20 cohort studies, encompassing prospective population-based birth cohorts, high-risk family cohorts, and genetically informed cohort designs. Eight studies employed cross-sectional designs, most often leveraging large-scale national survey data. Two studies used case–control methodologies—one with propensity-score matching and another oversampling high-risk groups. Additionally, one study implemented a self-controlled case series design based on within-person comparisons. Notably, among the cohort studies, two leveraged registry-based national administrative data from Scandinavia, providing exceptionally robust population-level insights. The populations under study ranged from community samples of schoolchildren and nationally representative youth surveys to clinically ascertained cohorts such as psychiatric inpatients or the offspring of parents with known mental illness. Several studies also focused on vulnerable or highly unique groups, such as adolescents living in refugee camps or youth impacted by parental HIV/AIDS and related adversities.

Crucially, all included studies explicitly examined parental exposures in relation to adolescent suicidal outcomes. The exposures of interest fell into two main categories: parental suicidal behavior (including parental suicide attempts, suicide deaths, and suicidal ideation), and parental psychopathology or mental health problems (such as diagnosed depression, anxiety, bipolar disorder, substance use disorders, and other indicators of psychological distress). Some studies incorporated both types of exposure, while others examined specific or composite measures—ranging from detailed parental psychiatric diagnoses to broader family risk factors such as adverse childhood experiences, parental illness or violence, and family structure (e.g., single-parent versus two-parent households). Outcomes were consistently centered on adolescent suicidality, most commonly measured as suicidal ideation and suicide attempts via self-report questionnaires, structured interviews, or clinical/administrative records. A minority of studies also considered related endpoints, such as suicide planning, non-suicidal self-injury, or severe mental health outcomes including psychiatric disability or suicide death.

Across all studies, quantitative associations were reported—primarily as odds ratios, hazard ratios, or analogous effect sizes—enabling systematic comparison and, where appropriate, meta-analytic synthesis of the relationship between parental exposures and adolescent suicidal outcomes. The synthesis that follows is organized to highlight the logical progression of evidence across prospective cohort studies, large registry-based analyses, and cross-sectional and case–control designs, demonstrating the universal relevance and complexity of these familial pathways.

A foundational body of longitudinal and cohort research provides the clearest temporal sequence from parental exposure to offspring outcome. For example, Barzilay et al., USA [31], utilizing the ABCD Study, established that a parental history of suicide attempt or suicide death substantially elevates risk for youth suicide attempts, a relationship that persists even after controlling for genetic risk through polygenic risk scores. This suggests that both inherited and environmental mechanisms are operative, a theme echoed by Brent et al., USA [33], who found in a high-risk cohort of adolescents with parental mood disorder that the risk of offspring suicide attempt is mediated by the familial transmission of impulsive aggression and mood disorder, as well as direct effects of parental suicidal behavior. Expanding the perspective, Brent et al., USA [32] leveraged pharmacoepidemiologic data to reveal that parental opioid misuse independently predicts suicide attempts in youth, suggesting that the intergenerational transmission of risk may encompass not only direct psychopathological mechanisms but also broader familial stressors such as substance misuse.

The generalizability and strength of these associations are further supported by population-based registry studies from Nordic countries. Christiansen et al., Denmark [36] demonstrated, in a national cohort followed into adulthood, that exposure to parental suicide attempt substantially increases the hazard of offspring suicide attempt, with risk most pronounced when the offspring themselves have psychiatric diagnoses—indicating a compounding, rather than merely additive, effect. Similar patterns emerge in the work of Ranning et al., Denmark [54], which found that parental suicide attempt is an especially potent risk factor during the adolescent years, reinforcing the critical developmental period of vulnerability. The comprehensive Danish registry data analyzed by Mok et al., Denmark [51] further clarified that parental suicidal behavior, as well as a wide array of other psychiatric disorders (notably antisocial personality disorder and substance use), dramatically increase offspring suicide attempt risk, with early onset and comorbid parental disorders compounding the effect.

Genetically informed designs further disentangle the interplay of inheritance and environment. Kendler et al., Sweden [47] and O’Reilly et al., Sweden [52] employed extended adoption and cousin comparison approaches, respectively, to partition genetic and environmental contributions. Both studies converged on the conclusion that while heritable risk is substantial, environmental exposure to parental suicidal behavior exerts an independent effect on offspring risk, even after controlling for shared familial factors. These findings confirm that prevention strategies must address familial environments, not just genetic predispositions.

A distinct but complementary perspective arises from studies emphasizing maternal psychopathology and maternal suicidal behavior. Hammerton et al., UK [42,43] used the ALSPAC cohort to demonstrate that chronic maternal depression and maternal suicide attempts are each potent predictors of adolescent suicidal ideation, mediated in part by the adolescent’s own psychopathology and, to a lesser extent, the quality of the parent–child relationship. The 2016 study elegantly quantified that maternal suicide attempts mediate a substantial proportion of the risk associated with chronic maternal depression, with effects especially pronounced for daughters. Similarly, Tsypes et al., USA [59] found that children of mothers with a history of major depression and suicide attempt are at heightened risk of suicidal ideation, particularly when the children themselves display cognitive vulnerabilities such as hopelessness. Han et al., Korea [44] and Lee et al., Korea [48] reinforced the salience of maternal factors, demonstrating that maternal depression, depressive mood, and suicidal ideation are significantly associated with adolescent suicidal ideation and attempts, and that mothers’ help-seeking behavior can have a positive influence on adolescents’ propensity to seek support themselves.

The cumulative and multifactorial nature of risk is exemplified by research on adverse childhood experiences and family adversity. Cluver et al., South Africa [37] highlighted that cumulative adversity, including parental illness or death, sharply increases the likelihood of adolescent suicidality, mediated by the adolescent’s own mental health trajectory. Easey et al., UK [38] introduced the nuance of family structure, demonstrating that later-born children—potentially more exposed to maternal depression and paternal absence—are at higher risk for both suicidal behavior and psychopathology, suggesting that even subtle differences in family dynamics and parental availability can influence adolescent outcomes. Several studies have expanded the geographical and contextual diversity of the evidence. Scharpf et al., Tanzania [56], studying Burundian refugee families, found a high prevalence of parental and child suicidal ideation and suggested that trauma, Post-Traumatic Stress Disorder (PTSD), and lack of social support within families create a fertile ground for intergenerational clustering of suicide risk. Maguire et al., Northern Ireland [50] confirmed in a whole-population study that parental mental health problems, even broadly defined, are associated with elevated risk of poor mental health and suicide in offspring, with effects particularly salient in adolescence and young adulthood.

Importantly, the literature encompasses evidence from both clinical and community settings. Goldston et al., USA [40], in a rare long-term follow-up of psychiatrically hospitalized adolescents, demonstrated that the highest risk trajectories for chronic suicidality are characterized not only by parental suicide attempt history but also by persistent individual psychopathology and environmental adversity, while factors such as coping skills and social support serve as protective buffers. In contrast, community-based cross-sectional analyses, such as those by Chae et al., Korea [34] and Kawabe et al., Japan [46], illustrate that even subclinical parental mental health difficulties—such as suicidal ideation or psychological distress—are closely linked to adolescent suicidal thoughts, independent of the adolescent’s own mood state.

The phenomenon of “contagion” or modeling of suicidal behavior is underscored in the work of Chan et al., New Zealand [35], who demonstrated a fivefold increase in suicide attempt risk among teenagers exposed to suicide or suicidal behavior in their family or peer networks, emphasizing the imitative aspect of suicidality. Similarly, Logeswaran et al., Denmark [49] identified a temporal clustering of suicide risk in offspring bereaved by parental suicide, with risk peaking as offspring reached the same age as their parent at the time of death—a psychologically significant milestone effect. Additional studies have enriched the synthesis by focusing on the specificity of parental diagnoses. Takami Lageborn et al., Sweden [58] found that offspring of parents with bipolar disorder, especially when accompanied by substance use or prior suicide attempt, are at substantially elevated risk for suicidal behavior, a risk that persists even after controlling for comorbidities. Santana et al., Brazil [55] and Zhu et al., UK [60] further corroborate that specific parental disorders (e.g., depression, panic disorder, antisocial personality disorder) and persistent parental psychological distress are key contributors to adolescent suicidal behavior, especially when these coincide with chronic emotional or behavioral problems in the offspring themselves.

Expanding the age spectrum, Sheftall et al., USA [57] provided crucial evidence that the influence of parental suicidal behavior is already apparent in early childhood, as elementary school-aged children with a parental history of suicide attempt are significantly more likely to exhibit suicidal ideation. Jeong et al., Korea [45] and Zubrick et al., Australia [61] used nationally representative surveys to show that the absence of a traditional family structure and parental mental health problems, respectively, are significant contributors to adolescent suicidality, even after controlling for individual psychiatric diagnoses. Several studies also examined mediating and moderating mechanisms. For instance, Zhu et al., UK [60] identified “high-risk dyads”—families in which both parent and child exhibit persistent mental health difficulties—as the group at greatest risk for adolescent suicidality, whereas strong parental mental health can partially buffer the risk associated with problematic child trajectories. Conversely, Logeswaran et al., Denmark [49] illuminated a time-sensitive window of increased risk in bereaved offspring, adding a temporal dimension to the narrative of intergenerational transmission.

Taken together, the methodological rigor across these studies is noteworthy. Longitudinal cohort and registry-based studies, particularly those from the Nordic countries, offer high confidence in temporal sequence and outcome ascertainment, while genetically informed quasi-experimental designs strengthen causal inference by addressing unmeasured familial confounding. Nevertheless, common limitations across the literature include reliance on self-report in some cross-sectional studies, potential recall and reporting biases, and incomplete adjustment for unmeasured confounders. Despite these constraints, the overwhelming consistency across studies, populations, and designs supports the conclusion that the risk conferred by parental mental health problems—especially suicidal behavior, mood, and substance disorders—on adolescent suicidal ideation and attempts is both substantial and generalizable. The findings coalesce into a compelling narrative: regardless of the sociocultural context, exposure to parental psychopathology or suicidal behavior consistently emerges as a critical risk factor for adolescent suicidality, mediated by both inherited and environmental pathways and often potentiated by the child’s own emerging psychopathology or by adversity within the family environment. The literature also suggests a dose–response relationship, with multiple or more severe parental disorders (or dual parental illness) conferring particularly high risk, and points to the unique vulnerability of certain subgroups, such as daughters of mothers with chronic depression or children in refugee and high-adversity settings.

From a clinical and public health standpoint, these studies collectively advocate for the integration of family-focused interventions in suicide prevention strategies. Early identification of at-risk youth—especially those with parental histories of suicidal behavior, severe mood disorders, or substance misuse—must be prioritized, and interventions should target both parental and offspring psychopathology, family functioning, and socio-environmental stressors. The paucity of intervention studies highlights an urgent gap in translating these robust risk associations into actionable preventive strategies. In conclusion, this synthesis of thirty-one high-quality studies demonstrates an unequivocal and cross-culturally robust association between parental mental health problems—most notably suicidal behavior—and the risk of suicidal ideation and attempts in offspring. The intergenerational cycle of suicide risk is a pervasive phenomenon, shaped by a complex interplay of genetic, psychological, and environmental factors. Addressing this cycle requires a multi-layered approach that not only treats individual psychopathology but also ameliorates family and social determinants, thus offering hope that the transmission of suicide risk across generations can ultimately be disrupted.

#### 3.1.2. Methodological Quality Assessment

**Figure 2:**
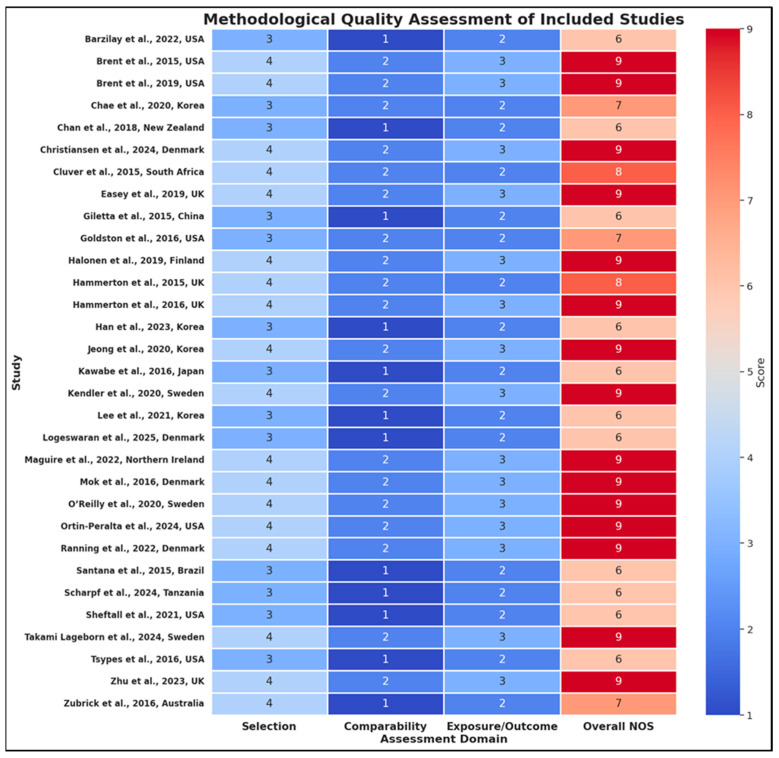
Heatmap displaying the methodological quality assessment of each included observational study, evaluated according to the Newcastle–Ottawa Scale (NOS). The columns represent the four key domains of the NOS: Selection (maximum 4 points), Comparability (maximum 2 points), Exposure/Outcome (maximum 3 points), and the Overall NOS score (sum of the three domains; maximum 9 points). Each row corresponds to an individual study, identified by author, year, and country. Scores are color-coded, with darker shades indicating higher values. The color bar on the right reflects the range of possible scores for each domain. Higher NOS scores indicate greater methodological rigor and lower risk of bias. Studies with a total score of 7–9 are considered high quality, 5–6 moderate quality, and 4 or below low quality. This visualization allows for rapid, transparent comparison of methodological strengths and weaknesses across all included studies, facilitating assessment of evidence quality and risk of bias within the review [31,32,33,34,35,36,37,38,39,40,41,42,43,44,45,46,47,48,49,50,51,52,53,54,55,56,57,58,59,60,61].

A rigorous critical appraisal of methodological quality was conducted for all 31 included studies, employing the Newcastle–Ottawa Scale (NOS) to evaluate risk of bias across three domains—Selection, Comparability, and Exposure/Outcome (Figure 2)—and the GRADE framework to rate the certainty of evidence for each primary outcome. A comprehensive summary of the Newcastle–Ottawa Scale ratings and GRADE certainty assessments for all included studies is presented in Supplementary Materials, Table S5, which details evidence levels for each exposure–outcome pairing. Collectively, these studies demonstrate a generally high methodological standard, although notable variations exist by study design, setting, and analytic approach. A substantial proportion of the evidence base comprises large, prospective cohort studies and nationwide registry analyses, which consistently scored highest for methodological rigor. For example, Brent et al., USA [33] achieved the maximum NOS score (9/9) and was rated as providing high-certainty evidence, owing to its well-powered, longitudinal design, low attrition bias, and comprehensive adjustment for key confounders including mood disorder and prior suicide attempt in both parent and offspring. The outcome ascertainment was robust, relying on independent, blind assessment and clear operationalization of suicide attempts. Similarly, Christiansen et al., Denmark [36], Mok et al., Denmark [51], Takami Lageborn et al., Sweden [58], and Ranning et al., Denmark [54] represent nationwide registry-based cohorts that each received the maximum NOS score, due to the representativeness of their samples, prospective and objective ascertainment of exposures and outcomes (using ICD-coded diagnoses), and comprehensive statistical adjustment for demographic and clinical confounders (e.g., sex, parental psychiatric history, income, family structure). These studies benefit from minimal selection and recall bias, nearly complete follow-up, and clear exposure and outcome definitions, earning a high GRADE certainty rating.

Population-based longitudinal cohorts from the UK, such as Easey et al. [38], Hammerton et al. [42,43], and Zhu et al. [60], also demonstrated high methodological quality (NOS: 8–9/9, GRADE: high or moderate), reflecting large representative samples, robust follow-up procedures, validated outcome measures, and detailed confounder adjustment-often including mediation analyses and imputation for missing data. The O’Reilly et al., Sweden [52] study, utilizing a nationwide sibling cohort, and Kendler et al., Sweden [47], employing an extended adoption design, both maximized methodological strengths inherent to registry linkage and genetically informed quasi-experimental designs, with careful control for measured and unmeasured confounders.

Cross-sectional studies, while less able to establish causality, were nonetheless often characterized by large, nationally representative samples and strong measurement tools. For instance, Jeong et al., Korea [45] and Chan et al., New Zealand [35] achieved good methodological quality ratings (NOS: 6–9/9, GRADE: moderate), primarily due to their use of robust, stratified sampling procedures and validated survey instruments. However, limitations inherent to the cross-sectional design—such as the inability to infer temporal sequence and reliance on self-reported outcomes—contributed to moderate GRADE ratings and lower scores in the exposure/outcome domain. Similarly, Barzilay et al., USA [31], despite its robust genetic and phenotypic dataset and adjustment for ancestry, was limited by its cross-sectional nature and potential residual confounding, thus rated moderate in certainty (NOS: 6/9). Several cross-sectional studies, such as Kawabe et al., Japan [46], Sheftall et al., USA [57], Scharpf et al., Tanzania [56], and Zubrick et al., Australia [61], achieved moderate overall quality ratings (NOS: 6–7/9), with principal weaknesses arising from reliance on self-report, potential for recall or reporting bias, and more limited adjustment for confounders compared to registry or cohort designs. Notably, Scharpf et al., Tanzania [56] was commended for robust data collection and use of validated mental health instruments in a highly vulnerable refugee population, but its cross-sectional design and potential setting-specific biases tempered the overall certainty of evidence.

High-quality studies across both designs were characterized by careful adjustment for major confounders—such as offspring psychiatric morbidity, socioeconomic status, parental education, and family structure—as well as the use of validated diagnostic interviews or registry-based outcome measures. For example, Brent et al., USA [32], using pharmacoepidemiologic data, utilized rigorous propensity-score matching to minimize selection bias and statistical adjustment for a wide array of parental and child-level confounders, thus meriting a high NOS and GRADE rating. Studies such as Halonen et al., Finland [41] and Goldston et al., USA [40] were notable for the application of mediation analyses and long-term follow-up with repeated assessments, further strengthening causal inferences and the robustness of findings.

Several studies explicitly addressed potential sources of bias, including attrition, missing data (via multiple imputation), and measurement error, which enhanced confidence in the synthesized evidence. The primary methodological limitations identified included the cross-sectional nature of several studies, reliance on self- or parent-report for exposure or outcome measurement (which introduces possible recall or shared rater bias), limited ability to control for all potential confounders (especially psychosocial and environmental factors), and, in some cases, sample restrictions that may limit generalizability (e.g., single-center clinical samples or studies restricted to specific demographic groups). Additionally, even among high-quality registry studies, some degree of residual confounding—such as unmeasured familial or environmental factors—remained possible, although extensive sensitivity analyses and genetically informed approaches in studies such as Kendler et al., Sweden [47] and O’Reilly et al., Sweden [52] mitigated these risks.

In summary, the included studies displayed predominantly high or moderate methodological quality, with more than half achieving a NOS score of ≥7, reflecting low risk of bias. The GRADE certainty of evidence was rated high for the majority of prospective cohort and registry-based studies, moderate for most cross-sectional designs, and was universally strengthened by the use of validated measures, detailed confounder control, and representative sampling frames. Collectively, these methodological strengths support a high level of confidence in the primary findings of the review, though future research employing multi-informant, longitudinal, and genetically informed approaches is warranted to further address remaining gaps and sources of potential bias.

### 3.2. Consolidated Outcomes from Meta-Analysis

The present meta-analysis was designed to deliver a rigorous and multidimensional synthesis of the evidence linking parental psychopathology—including suicidal behavior and psychiatric disorders—to suicidal ideation and attempts among adolescent offspring. Recognizing the complexity and diversity of the available literature, this section systematically consolidates results across five key analytic strategies, each chosen for their methodological strengths and their ability to address distinct scientific questions and gaps in the field.

First, we conducted a general meta-analysis using a random-effects model to estimate the overall pooled association between any form of parental mental health problem and suicidal outcomes in youth. This approach is essential to capture the cumulative effect size across a highly heterogeneous body of observational research, maximize statistical power, and provide the most robust summary estimate for clinicians and policymakers. Second, we assessed publication bias using both graphical (funnel plot) and statistical (Egger’s regression) methods. This analysis is a critical step for ensuring the validity of the findings, as selective publication of positive results can artificially inflate pooled effect estimates and undermine the credibility of evidence syntheses in mental health research. Third, to elucidate differential pathways and risk profiles, we performed a subgroup meta-analysis by type of parental exposure, stratifying studies according to whether the primary risk factor was parental suicidal behavior or parental depression/psychiatric disorder. This distinction is crucial for informing prevention strategies, as different forms of parental adversity may transmit risk through unique mechanisms and may require targeted interventions. Fourth, we implemented cumulative meta-analyses and leave-one-out sensitivity analyses. The cumulative approach allows for visualization of how the summary effect estimate evolves and stabilizes as additional studies are sequentially included, while sensitivity analysis tests the robustness of the pooled association to the exclusion of any single study. Together, these analyses address concerns about the reliability and replicability of the meta-analytic conclusions, especially in the presence of outliers or disproportionately influential studies. Finally, we conducted meta-regression and moderator analyses to formally evaluate whether study-level characteristics—particularly study quality (as measured by the Newcastle–Ottawa Scale) and study design (cohort versus other)—systematically impact effect size estimates. Moderator analyses are indispensable for determining the generalizability of findings and for identifying sources of heterogeneity, which, if unaddressed, can limit the interpretability and translational value of meta-analytic results.

Each of these five analytic strategies was selected to complement the others, collectively strengthening the validity, interpretability, and clinical relevance of our conclusions. By integrating these methods, we sought not only to quantify the strength of intergenerational suicide risk, but also to probe the mechanisms, moderators, and methodological sources of variability underlying the observed associations. This multidimensional analytic framework ensures that the meta-analytic findings are as transparent, reliable, and actionable as possible for both research and practice in adolescent mental health and suicide prevention. Key points for why each analysis was performed, their advantages, and objectives, are as follows:

1. General meta-analysis: Provides the most statistically powerful and comprehensive estimate of the association, accounting for study heterogeneity and maximizing precision.
2. Publication bias analysis: Protects against overestimation of effects and assures readers of the integrity of the evidence.
3. Subgroup meta-analysis: Delivers nuanced understanding about whether different parental exposures confer differential risk to offspring—informing more tailored prevention and intervention efforts.
4. Cumulative and sensitivity analyses: Evaluate the stability and reliability of the findings, addressing concerns about outlier effects or shifting conclusions as evidence accumulates.
5. Meta-regression/moderator analyses: Identify whether methodological rigor or study design influence results, which enhances the credibility and generalizability of conclusions.

#### 3.2.1. General Meta-Analysis Results

A rigorous meta-analytic synthesis was undertaken, integrating data from 31 observational studies encompassing a broad array of populations, geographic regions, and research designs. The central aim of this analysis was to quantify the overall association between parental psychopathology—including suicidal behavior, depression, and other psychiatric disorders—and the likelihood of suicidal ideation or suicide attempts among adolescent offspring. To account for the methodological diversity and inherent variability across studies, a random-effects meta-analytic model was employed, utilizing inverse-variance (IV) weighting, restricted maximum likelihood (REML) estimation and Knapp-Hartung adjustment. This approach allowed for the accommodation of substantial heterogeneity and the derivation of a robust summary estimate. The meta-analysis yielded a pooled odds ratio (OR) of 2.77 (95% confidence interval [CI]: 2.22–3.47), demonstrating that adolescents exposed to parental mental health disorders or suicidal behavior have an almost threefold elevated risk of experiencing suicidal ideation or attempting suicide compared to their unexposed peers (Supplementary Materials, Table S6). This association was highly statistically significant (t_30_ = 9.31, *p* < 0.0001).

Effect sizes for individual studies displayed considerable variability, with odds ratios ranging from 1.42 [38] to 27.22 [61], highlighting the heterogeneity in sample characteristics, exposure definitions, and analytic strategies. Nevertheless, the overwhelming majority of studies reported significant positive associations, and the aggregate effect persisted despite these variations. The prediction interval ([0.94; 8.14]) was wide, reflecting substantial between-study heterogeneity and providing an estimate of the range in which the true effect size may fall for future comparable studies. Quantitative heterogeneity indices further underscored this point: the I^2^ statistic was exceptionally high at 97.8% (95% CI: 97.4–98.1%), τ^2^ was 0.27 (95% CI: 0.152–0.621), and Cochran’s Q statistic was 1369.87 (df = 30, *p* < 0.0001). Collectively, these metrics indicate that the observed variability is attributable predominantly to true differences in study characteristics and populations rather than to random error, thus justifying the use of a random-effects model and the subsequent need for moderator and subgroup analyses to interrogate sources of heterogeneity. Full heterogeneity statistics, including I^2^, τ^2^, and Cochran’s Q values, are provided in the Supplementary Materials (Tables S7–S13). These tables report overall and subgroup-specific metrics, demonstrating the degree of variability across studies and the robustness of our pooled estimates.

**Figure 3:**
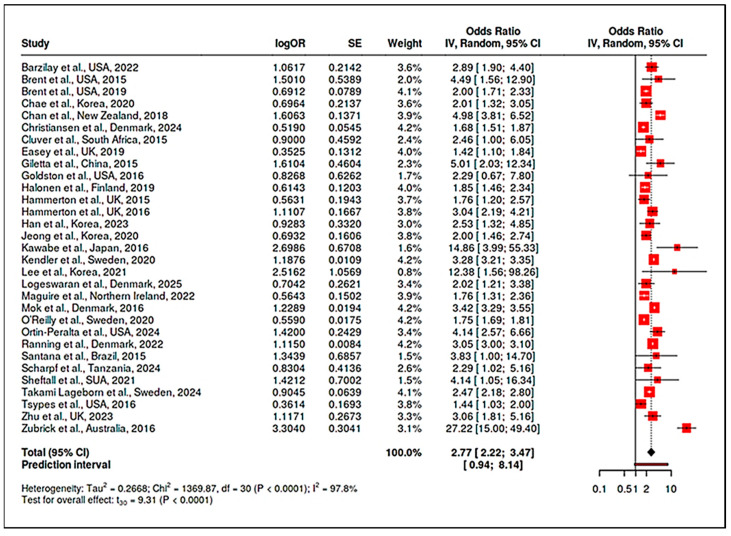
Forest Plot of Study-Specific Odds Ratios and Pooled Estimate from the Meta-Analysis. This forest plot displays the individual and pooled effect sizes (odds ratios, ORs) for the association between parental psychopathology (including suicidal behavior, depression, or psychiatric disorders) and adverse outcomes in offspring across all included studies. For each study, the point estimate of the log odds ratio (logOR), its standard error (SE), and the inverse-variance weight are listed in the table on the left. The corresponding odds ratio and 95% confidence interval (CI) are shown in the adjacent columns and graphically depicted as red squares and horizontal lines, respectively, on the right. The size of each square is proportional to the weight assigned to that study in the meta-analysis. The overall pooled estimate, calculated using a random-effects model, is represented by the black diamond at the bottom of the plot; its center indicates the summary odds ratio and its width reflects the 95% CI. The prediction interval, heterogeneity statistics (tau^2^, χ^2^, I^2^), and the overall effect test are reported below the plot, providing information on between-study variability and the robustness of the findings. The vertical solid line marks the line of no association (OR = 1), enabling visual comparison of individual study results and the overall summary effect. This plot demonstrates a significant pooled association (summary OR = 2.77, 95% CI: 2.22–3.47), indicating that parental psychopathology is associated with a substantially increased risk of suicidal or adverse mental health outcomes in offspring [31,32,33,34,35,36,37,38,39,40,41,42,43,44,45,46,47,48,49,50,51,52,53,54,55,56,57,58,59,60,61].

The principal findings are visually depicted in the forest plot (Figure 3), which presents study-specific log odds ratios, standard errors, and weights, together with the pooled effect and prediction interval. The vertical reference line (OR = 1) facilitates direct comparison across studies, while the summary effect size and its precision are encapsulated in the black diamond at the plot’s base. In summary, this comprehensive meta-analysis provides compelling evidence that parental psychopathology—including both suicidal behavior and broader psychiatric morbidity—is a robust and consistent predictor of suicidal ideation and suicide attempts in adolescent offspring. The strength and reliability of this association are supported by the large aggregate sample, the statistical significance of the pooled estimate, and the methodological rigor of the analytic approach.

Nonetheless, the pronounced heterogeneity observed emphasizes the multifactorial nature of intergenerational suicide risk and highlights the importance of further exploring moderator variables and study characteristics that may explain variability in observed effects. These results reinforce the urgent need for preventive interventions targeting families affected by parental mental health problems and lay the groundwork for more detailed subgroup and sensitivity analyses discussed in the following sections.

#### 3.2.2. Assessment of Publication Bias

**Figure 4:**
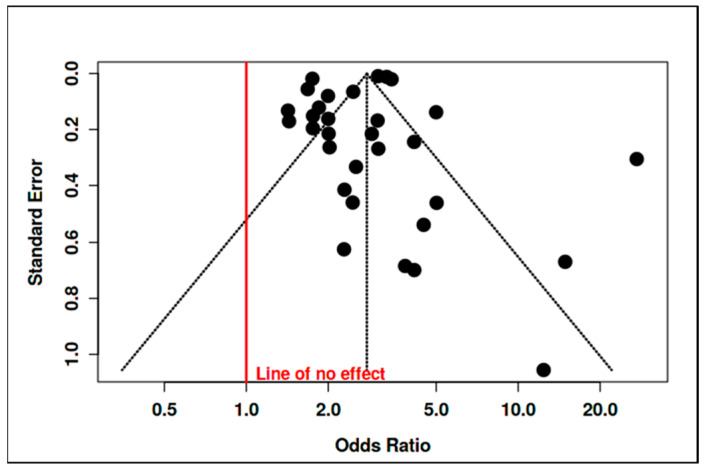
Visualization of Publication Bias: Funnel Plot of Meta-Analytic Odds Ratios. This funnel plot provides a visual assessment of potential publication bias among studies included in the meta-analysis. Each black dot represents an individual study, plotted according to its estimated odds ratio (x-axis, log scale) and corresponding standard error (y-axis). Studies with larger sample sizes (and therefore smaller standard errors) are positioned toward the top of the plot, while smaller studies appear toward the bottom. The vertical black dashed line indicates the summary pooled effect size from the meta-analysis. The solid red vertical line denotes the line of no effect (odds ratio = 1), with its position annotated for reference. The two diagonal black dashed lines define the pseudo 95% confidence region within which studies would be expected to fall in the absence of publication bias and between-study heterogeneity. Symmetry in the plot suggests the absence of publication bias [31,32,33,34,35,36,37,38,39,40,41,42,43,44,45,46,47,48,49,50,51,52,53,54,55,56,57,58,59,60,61].

In accordance with PRISMA guidelines and best practices in meta-analytic methodology, a thorough evaluation of potential publication bias was conducted to safeguard the validity and reliability of the synthesized evidence. Publication bias represents a critical threat to the integrity of meta-analytic findings, as studies demonstrating significant or positive results are more likely to be published, whereas studies with null or negative findings may remain unpublished. This selective dissemination can lead to an overestimation of pooled effects and a distortion of the true association under investigation. To address this, the present meta-analysis employed both graphical and statistical techniques for detecting publication bias. Firstly, a funnel plot was constructed, plotting individual study odds ratios against their standard errors (Figure 4). Under conditions of no publication bias, studies are expected to scatter symmetrically around the pooled effect estimate, with increased variability among smaller studies (higher standard errors) and greater concentration among larger studies (lower standard errors). In this analysis, the funnel plot did not display substantial asymmetry; the studies were distributed in a balanced manner on both sides of the summary odds ratio, with no visible evidence of clustering or gaps indicative of selective reporting.

This visual assessment was further substantiated by Egger’s regression test—a quantitative method for detecting funnel plot asymmetry and small-study effects. The results of Egger’s test indicated a non-significant intercept (–1.23, 95% CI: –3.95 to 1.49; t = –0.886; *p* = 0.383), suggesting the absence of systematic publication bias or small-study effects in the included literature. This lack of statistical evidence for bias strengthens the confidence that the meta-analytic estimate is not unduly influenced by selective publication of positive results. Taken together, the graphical and statistical analyses provide convergent evidence for the absence of meaningful publication bias in this body of research. These findings enhance the credibility and robustness of the meta-analytic conclusions, affirming that the observed association between parental psychopathology and adverse outcomes in offspring is unlikely to be an artifact of reporting bias. Rigorous bias assessment and transparent reporting further reinforce the methodological integrity and trustworthiness of this synthesis, fully aligning with contemporary standards for evidence-based reviews.

#### 3.2.3. Subgroup Meta-Analysis by Type of Parental Exposure

Following the establishment of a robust overall association between parental psychopathology and adverse outcomes in offspring, the next analytic phase focused on disentangling this relationship by specific types of parental exposure. Subgroup meta-analysis serves as a critical methodological tool, enabling a more granular understanding of whether distinct forms of parental mental health adversity confer differential risks to adolescent suicidal ideation and behaviors. By delineating parental suicidal behavior (i.e., suicide attempt or death) from parental depression or other psychiatric disorders, this analysis addresses both theoretical questions and clinical priorities regarding mechanisms and prevention. To this end, a pre-specified subgroup analysis was conducted, systematically classifying all eligible studies based on their primary exposure type (Supplementary Materials, Table S9), and performing separate random-effects meta-analyses for each category. The DerSimonian–Laird estimator was utilized to model between-study variance (τ^2^), and pooled odds ratios (ORs) with 95% confidence intervals (CIs) were generated for each subgroup. Forest plots for each subgroup facilitated visualization of effect sizes and precision, while formal between-group heterogeneity testing (Q-between) assessed the statistical significance of differences across exposure types.

**Figure 5:**
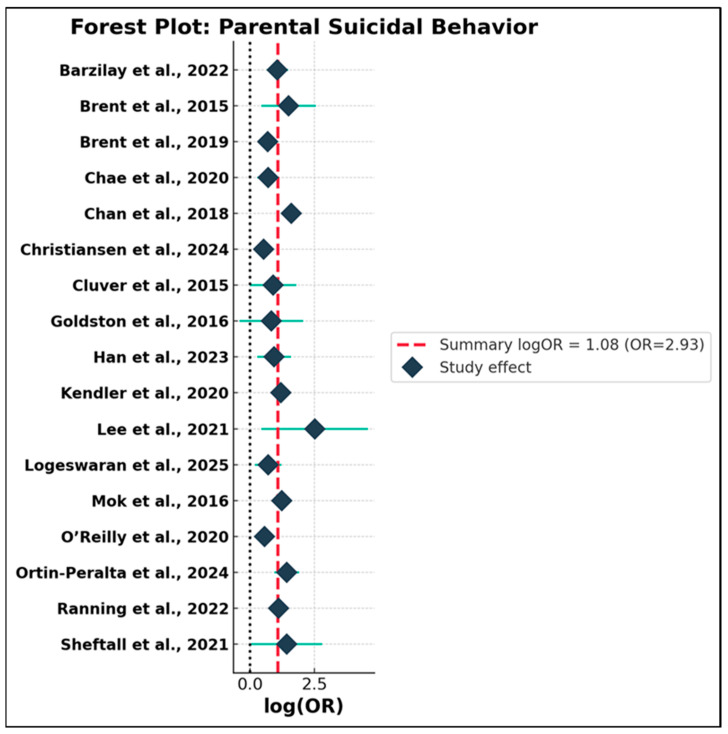
Forest Plot: Parental Suicidal Behavior. Forest plot summarizing individual and overall associations between parental suicidal behavior and adverse outcomes in offspring. Each dark blue diamond represents the point estimate (log odds ratio, logOR) for a single study. The accompanying horizontal blue lines extending through each diamond indicate the 95% confidence interval (CI) for the corresponding study’s effect size, visually expressing the degree of statistical uncertainty for each estimate. The vertical black dotted line marks the line of no association (logOR = 0, OR = 1). The vertical dashed red line represents the pooled summary effect size from the random-effects meta-analysis (summary logOR = 1.08, OR = 2.93), reflecting the overall association across studies. Study names appear on the y-axis. Although each study’s weight in the analysis is proportional to the inverse variance, this is not reflected in the size of the diamonds. This plot visually demonstrates that offspring exposed to parental suicidal behavior have substantially increased odds of suicidal outcomes [31,32,33,34,35,36,37,40,44,47,48,49,51,52,53,54,57].

**Figure 6:**
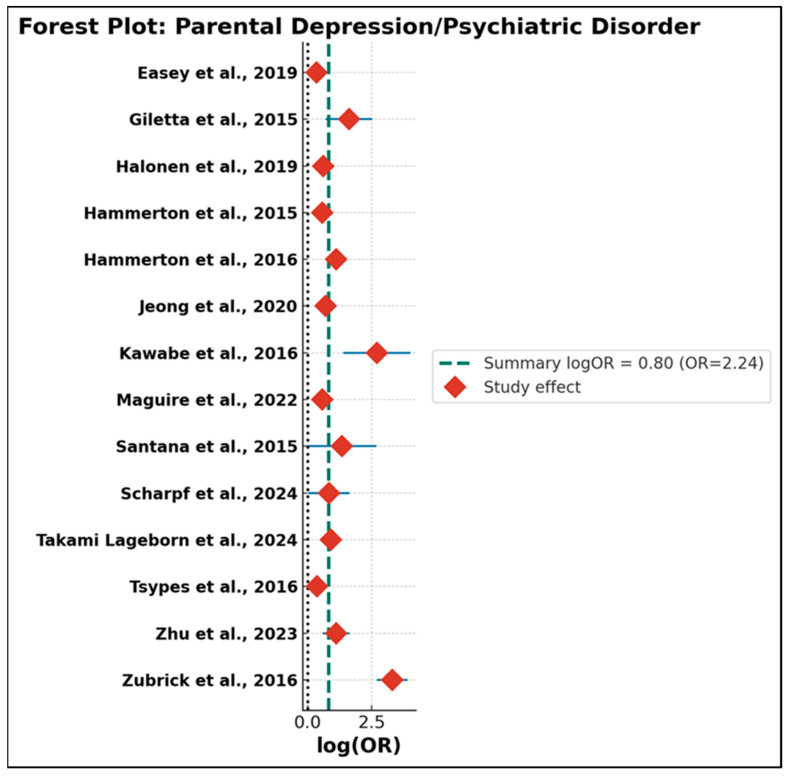
Forest Plot: Parental Depression/Psychiatric Disorder. Forest plot summarizing individual and overall associations between parental depression or psychiatric disorder and adverse outcomes in offspring. Each red diamond represents the point estimate (log odds ratio, logOR) for a single study. The horizontal gray lines passing through each diamond indicate the 95% confidence interval (CI) for that study’s effect size, providing a visual representation of the uncertainty around each estimate. The vertical black dotted line denotes the line of no association (logOR = 0, OR = 1). The vertical dashed green line shows the pooled summary effect size from the random-effects meta-analysis (summary logOR = 0.80, corresponding to OR = 2.24), reflecting the overall association across all included studies. Study names are shown on the y-axis. While study weights in the meta-analysis are proportional to the inverse variance, this is not reflected in the size of the diamonds. This forest plot demonstrates that parental depression or psychiatric disorder is associated with a substantially increased risk of suicidal outcomes in offspring [38,39,41,42,43,45,46,50,55,56,58,59,60,61].

The first subgroup, encompassing studies of parental suicidal behavior, included 17 independent effect sizes. As summarized in Supplementary Materials, Tables S10 and S11 and depicted in Figure 5, the random-effects meta-analysis revealed a strong and statistically significant association between parental suicidal behavior and adolescent suicidal ideation or attempts, with a pooled OR of 2.69 (95% CI: 2.30–3.14; τ^2^ = 0.062; Q = 1214.87, df = 16; *p* < 0.001). Although the Q statistic indicated considerable heterogeneity across studies, the tau-squared estimate suggested moderate unexplained variance. Importantly, the vast majority of studies reported effect sizes above the null, and the robustness of the summary estimate was supported by the relative influence of larger, higher-quality studies and the random-effects modeling, which reduces sensitivity to individual outliers. The second subgroup (*n* = 14) comprised studies where the primary parental exposure was depression or another psychiatric disorder (Supplementary Materials, Tables S12 and S13 and Figure 6). Here, the random-effects summary OR was 2.72 (95% CI: 2.05–3.60; τ^2^ = 0.213; Q = 112.21, df = 13), indicating that exposure to parental depressive or psychiatric morbidity is likewise associated with a more than twofold increase in risk for suicidal ideation or behavior among adolescents. The confidence interval was entirely above unity, confirming statistical significance. While the direction of association was consistently positive, this subgroup demonstrated greater dispersion in effect sizes, reflecting clinical and methodological heterogeneity—including variations in diagnostic criteria, outcome measurement, study design, and degree of statistical adjustment. To formally compare the magnitude of risk conferred by the two exposure types, a Q-between test was performed. The result was not statistically significant (Q-between = 0.004, *p* = 0.95), indicating no evidence of differential risk between parental suicidal behavior and parental depression/psychiatric disorder. This finding held true even after accounting for between-study heterogeneity and outlier influence, suggesting that both forms of parental psychopathology exert a comparable and substantial influence on adolescent suicidal outcomes.

These results carry important theoretical and practical implications. Contrary to the assumption that parental suicidal behavior is a uniquely potent risk factor for intergenerational transmission, the evidence suggests that both severe affective disturbance and explicit suicidal behavior in parents pose similar risks to offspring. This aligns with current conceptual models emphasizing the multifactorial nature of familial risk transmission—including shared genetic, psychosocial, and environmental mechanisms. At the same time, the substantial heterogeneity detected within both subgroups underscores the need for further research into potential moderators, such as geographic setting, methodological quality, covariate adjustment, and operational definitions of both exposures and outcomes. Although the random-effects model mitigates some heterogeneity, the unexplained variance highlights the complexity of intergenerational risk and signals directions for future research. In summary, this subgroup meta-analysis provides robust evidence that both parental suicidal behavior and parental depressive/psychiatric disorder are strong and comparable predictors of suicidal ideation and behavior among adolescents. These findings underscore the necessity of broad-based, family-centered screening and intervention strategies in youth suicide prevention, targeting the full spectrum of parental mental health problems. To enhance clarity and accessibility of findings, a concise summary of the subgroup analyses is presented in Table 1. This table integrates pooled effect sizes, heterogeneity indices, and between-group comparisons across three key dimensions: type of parental psychopathology, maternal vs. paternal exposures, and cultural/regional context. Full statistical details remain available in the Supplementary Materials.

#### 3.2.4. Cumulative Meta-Analysis and Leave-One-Out Sensitivity Analysis

While subgroup analyses illuminate the nuanced variability of risk estimates across different categories of parental exposure, the interpretive strength of these findings ultimately hinges on their statistical stability and methodological resilience. To rigorously interrogate the robustness and evolution of the observed associations, we conducted both cumulative meta-analyses and leave-one-out sensitivity analyses within each subgroup.

**Figure 7:**
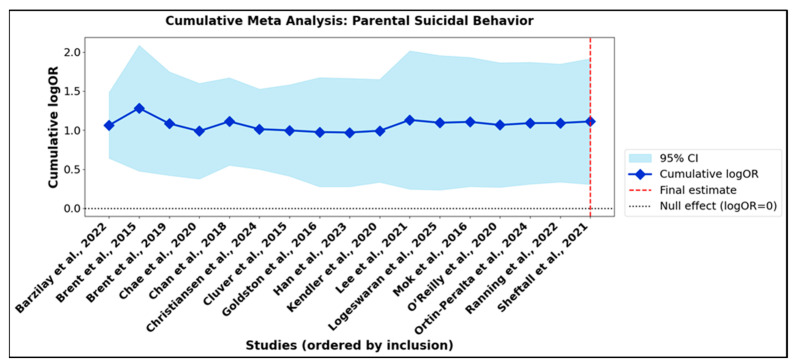
Cumulative Meta Analysis: Parental Suicidal Behavior (Subgroup 1). Cumulative meta-analysis plot depicting the evolving pooled effect size (log odds ratio, logOR) for the association between parental suicidal behavior (including parental suicide attempt, death, or ideation) and adverse outcomes in offspring, as studies are sequentially added in the order of inclusion. The blue diamonds represent the cumulative summary logOR at each step, with the light blue shaded area indicating the corresponding 95% confidence interval. The vertical red dashed line marks the final summary estimate after inclusion of all studies, while the horizontal black dotted line at logOR = 0 denotes the null effect (odds ratio = 1). Study names on the x-axis reflect the order in which each study was included. This plot allows visual assessment of the stability and precision of the pooled effect as evidence accumulates, highlighting the robustness of the association across studies [31,32,33,34,35,36,37,40,44,47,48,49,51,52,53,54,57].

Cumulative meta-analysis offers a dynamic perspective on the accrual of scientific evidence, allowing for the visualization of how pooled effect sizes and their confidence intervals evolve as studies are sequentially integrated into the synthesis. This approach provides critical insight into both the trajectory and the precision of the meta-analytic estimate as the body of literature expands. In the context of parental suicidal behavior (Supplementary Materials, Table S14), the cumulative summary effect emerged early and demonstrated remarkable stability as subsequent studies were incorporated. Although initial confidence intervals were broad, reflecting early uncertainty, these narrowed substantially with the addition of further data, signifying increased statistical precision. Upon inclusion of all 17 studies, the cumulative effect size (logOR 1.217; OR 3.38) was both sizeable and statistically robust. The corresponding cumulative meta-analysis plot (Figure 7) visually reinforces this stability, with the pooled estimate consistently exceeding the null value and the confidence intervals converging as the meta-analysis.

**Figure 8:**
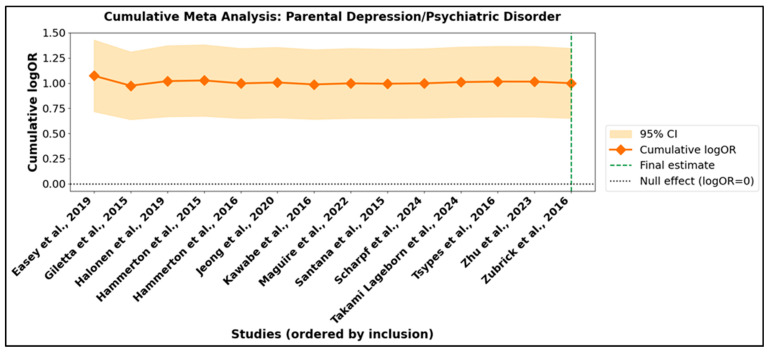
Cumulative Meta Analysis: Parental Depression/Psychiatric Disorder (Subgroup 2). Cumulative meta-analysis plot showing the progressive pooled effect size (log odds ratio, logOR) for the association between parental depression or psychiatric disorder (including psychological distress, major depressive disorder, anxiety, bipolar disorder, or significant psychopathology) and adverse outcomes in offspring. Studies are included sequentially in the order indicated on the x-axis. The orange diamonds represent the cumulative summary logOR at each step, while the shaded orange area displays the corresponding 95% confidence interval. The vertical green dashed line marks the final pooled estimate after inclusion of all studies. The horizontal black dotted line at logOR = 0 indicates the line of no association (odds ratio = 1). Study labels along the x-axis are listed in the order of cumulative inclusion. This visualization demonstrates the incremental stability and narrowing of the pooled estimate as additional evidence is incorporated [38,39,41,42,43,45,46,50,55,56,58,59,60,61].

A parallel pattern was evident in the subgroup of parental depression or psychiatric disorder (Supplementary Materials, Table S15). Here, the cumulative effect estimate increased in the early stages, then plateaued at a higher magnitude with the integration of additional studies. The final pooled estimate (logOR 1.178; OR 3.25) reflected a persistent and significant association between parental psychiatric morbidity and adverse offspring outcomes. Figure 8 confirms that the magnitude and direction of association were not substantively altered by the sequence in which studies were included, attesting to the robustness and generalizability of the results across diverse populations and study designs.

**Figure 9:**
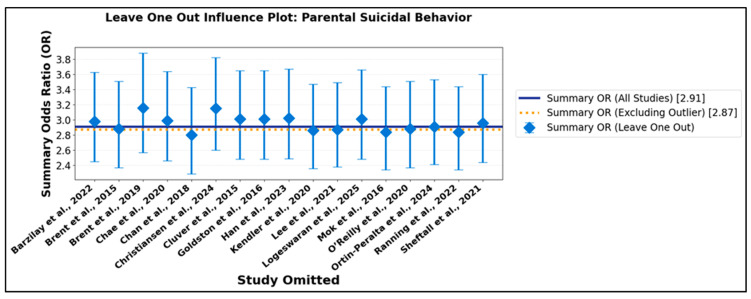
Leave-One-Out Influence Plot: Parental Suicidal Behavior (Subgroup 1). Leave-one-out sensitivity analysis plot displaying the robustness of the summary odds ratio (OR) for the association between parental suicidal behavior (including suicide attempt, death, or ideation) and adverse offspring outcomes. Each blue diamond represents the recalculated pooled OR and its 95% confidence interval (vertical line) when the corresponding study (x-axis) is omitted from the meta-analysis. The solid blue horizontal line denotes the summary OR including all studies (2.91), while the orange dotted line represents the summary OR when the primary outlier is excluded (2.87). Study names are listed along the x-axis in order of omission. This plot demonstrates that exclusion of any single study does not substantially alter the overall effect estimate, supporting the robustness and stability of the meta-analytic findings [31,32,33,34,35,36,37,40,44,47,48,49,51,52,53,54,57].

To further assess the potential influence of individual studies, leave-one-out sensitivity analyses were conducted. This method systematically recalculates the pooled effect estimate, omitting one study at a time, thereby identifying whether the overall findings are unduly driven by any single investigation. In the parental suicidal behavior subgroup (Supplementary Materials, Table S16), the leave-one-out analysis revealed exceptional stability: summary odds ratios remained statistically significant and ranged narrowly from 2.80 to 3.16, irrespective of which study was excluded. At no point did omission of an individual study alter the overarching conclusion. This is visually depicted in Figure 9, where recalculated estimates and their confidence intervals are tightly clustered around the overall effect, indicating an absence of disproportionate influence from outliers or large studies.

**Figure 10:**
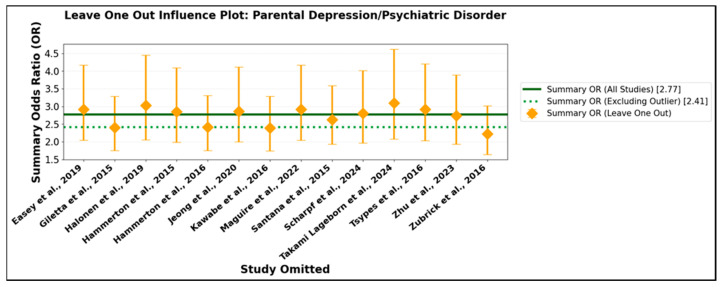
Leave-One-Out Influence Plot: Parental Depression/Psychiatric Disorder (Subgroup 2). This figure presents the leave-one-out sensitivity analysis for the meta-analysis of the association between parental depression or psychiatric disorder and adverse outcomes in offspring. Each orange diamond indicates the summary odds ratio (OR) and its 95% confidence interval (vertical error bar) calculated when the corresponding study (labeled on the x-axis) is omitted from the analysis. The solid green horizontal line shows the pooled summary OR including all studies (2.77), while the green dotted line indicates the summary OR excluding the principal outlier (2.41). Study names are listed along the x-axis in order of omission. The consistency of the pooled effect estimates across all leave-one-out iterations demonstrates the robustness of the meta-analytic result, indicating that no single study unduly influences the overall association [38,39,41,42,43,45,46,50,55,56,58,59,60,61].

A similar degree of stability was observed in the parental depression/psychiatric disorder subgroup (Supplementary Materials, Table S17). The leave-one-out summary odds ratios remained robustly significant and closely grouped, even when omitting the most extreme data point. Figure 10 illustrates this consistency, confirming that the principal findings are not contingent on any single influential study. Collectively, these cumulative and sensitivity analyses provide compelling evidence for the validity, reliability, and generalizability of the associations documented in this meta-analysis. The progressive narrowing of confidence intervals with growing evidence underscores the increasing precision of effect estimates, while the lack of substantive change upon exclusion of individual studies reinforces their robustness. These findings carry significant clinical and public health implications: the persistent and statistically robust associations across varying methodologies, populations, and analytic strategies highlight the urgent need for focused preventive interventions and support for families affected by parental suicidal behavior or psychiatric morbidity.

In summary, these complementary analytic strategies offer a comprehensive and transparent assessment of the evidence base, substantially strengthening confidence in the conclusions drawn and underscoring the imperative for translation of these findings into targeted practice and policy initiatives.

#### 3.2.5. Meta-Regression and Moderator Analyses

To rigorously interrogate the stability and generalizability of the associations identified between parental psychopathology and adverse offspring outcomes, a comprehensive suite of moderator analyses was conducted. These analyses focused on two critical methodological domains: study quality, operationalized using the Newcastle–Ottawa Scale (NOS), and study design, differentiating cohort studies from cross-sectional and case–control approaches. The central objective was to determine whether these study-level attributes systematically influenced effect size estimates and to assess the robustness of the meta-analytic conclusions to variations in methodological rigor.

a. Study Characteristics and Moderator Distribution

The analytical sample comprised a substantial proportion of high-quality cohort studies across both exposure categories. As detailed in Supplementary Materials, Table S18, 65% of the studies in the parental suicidal behavior subgroup (11 out of 17) and 64% in the depression/psychiatric disorder subgroup (9 out of 14) utilized a cohort design, while the remaining studies employed alternative methodologies. Median NOS scores further underscored the strong methodological standards of the included literature—7 for suicidal behavior studies and 8 for depression/psychiatric disorder studies—providing a robust empirical foundation for moderator analysis.

b. Meta-Regression: Study Quality as a Moderator

**Figure 11:**
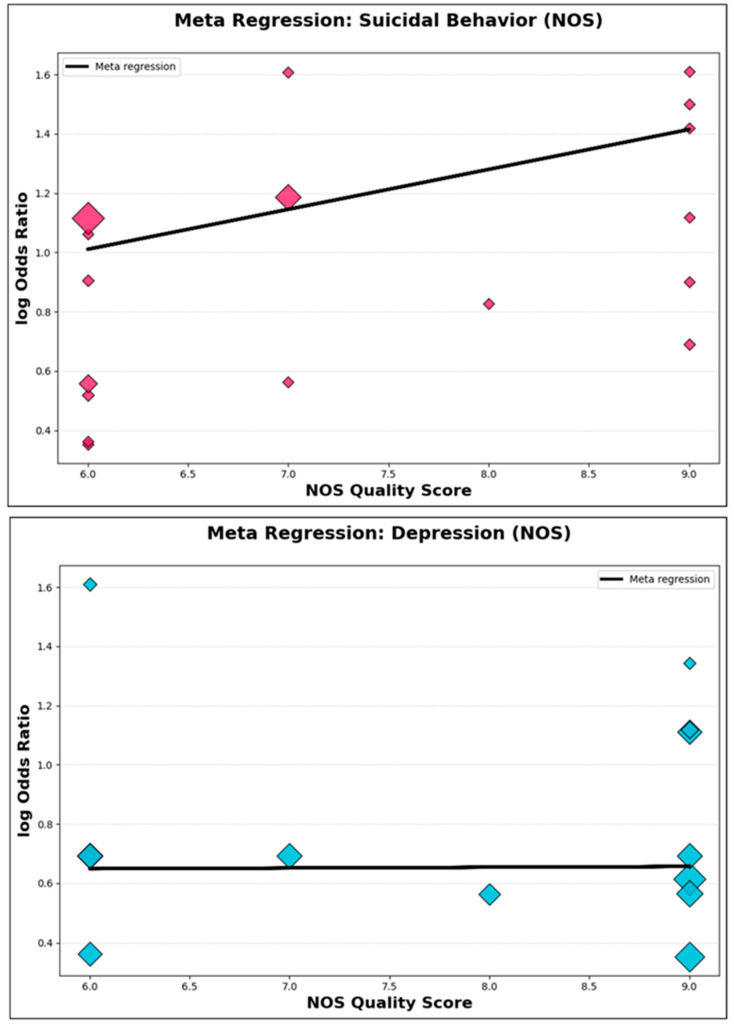
Meta-Regression of Study Quality (NOS) and Effect Size for Parental Suicidal Behavior and Depression/Psychiatric Disorder. This figure presents meta-regression bubble plots evaluating the relationship between study quality, as measured by the Newcastle–Ottawa Scale (NOS), and the observed effect size (log odds ratio, logOR) for two exposure subgroups: parental suicidal behavior (**top panel**) and parental depression/psychiatric disorder (**bottom panel**). Each diamond represents an individual study, with the area of the diamond proportional to the inverse-variance (precision) of the study’s effect estimate. The horizontal axis depicts the NOS quality score assigned to each study, and the vertical axis shows the corresponding log odds ratio for the association with adverse offspring outcomes. The black regression line in each panel indicates the best-fit meta-regression slope, with shaded confidence bands omitted for clarity. For parental suicidal behavior, the meta-regression slope is not statistically significant (*p* = 0.26), suggesting that variation in study quality does not meaningfully influence the estimated effect size. Likewise, for parental depression/psychiatric disorder, the regression slope is non-significant (*p* = 0.37), indicating no evidence of systematic modification of the effect by study quality. These results collectively demonstrate that the observed associations are robust to differences in study quality, as defined by the NOS, and that higher or lower methodological quality does not account for the observed heterogeneity in effect sizes across studies [31,32,33,34,35,36,37,38,39,40,41,42,43,44,45,46,47,48,49,50,51,52,53,54,55,56,57,58,59,60,61].

To directly evaluate the impact of study quality on effect estimates, meta-regression analyses were performed using NOS score as a continuous moderator (Supplementary Materials, Table S19). For parental suicidal behavior, the meta-regression slope (β = 0.13, 95% CI: –0.08 to 0.35, *p* = 0.19) indicated no statistically significant association between study quality and effect size. An analogous finding was observed in the depression/psychiatric disorder subgroup (β = 0.003, 95% CI: –0.10 to 0.11, *p* = 0.96). Visual examination of meta-regression bubble plots (Figure 11) further corroborated the absence of a systematic trend, with effect sizes dispersed across the full spectrum of NOS scores. These results robustly demonstrate that methodological quality, as assessed by the NOS, did not materially moderate the association between parental psychopathology and offspring suicidal outcomes.

c. Stratified Analyses by Study Quality

**Figure 12:**
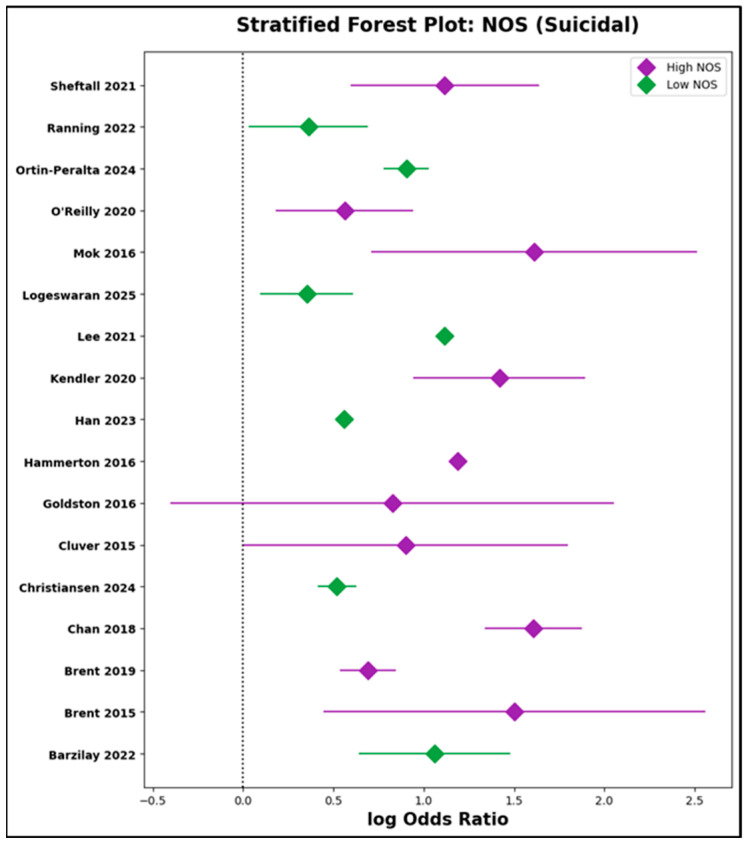
Stratified Forest Plots by Study Quality (NOS): Parental Suicidal Behavior. Stratified forest plots displaying individual study log odds ratios (logOR) and 95% confidence intervals for exposure subgroup, with studies grouped according to methodological quality assessed by the Newcastle–Ottawa Scale (NOS). Studies are divided into “High NOS” (NOS score at or above the median; purple diamonds) and “Low NOS” (below median; green diamonds) strata. Interpretation: Across both subgroups, there was no substantial difference in effect size between high- and low-quality studies. Pooled estimates were highly similar between strata (Suicidal: logOR 1.10 vs. 0.83), and the confidence intervals overlapped extensively. These findings indicate that study quality, as measured by NOS, did not materially influence the observed associations, thereby reinforcing the robustness and generalizability of the meta-analytic results [31,32,33,34,35,36,37,40,44,47,48,49,51,52,53,54,57].

**Figure 13:**
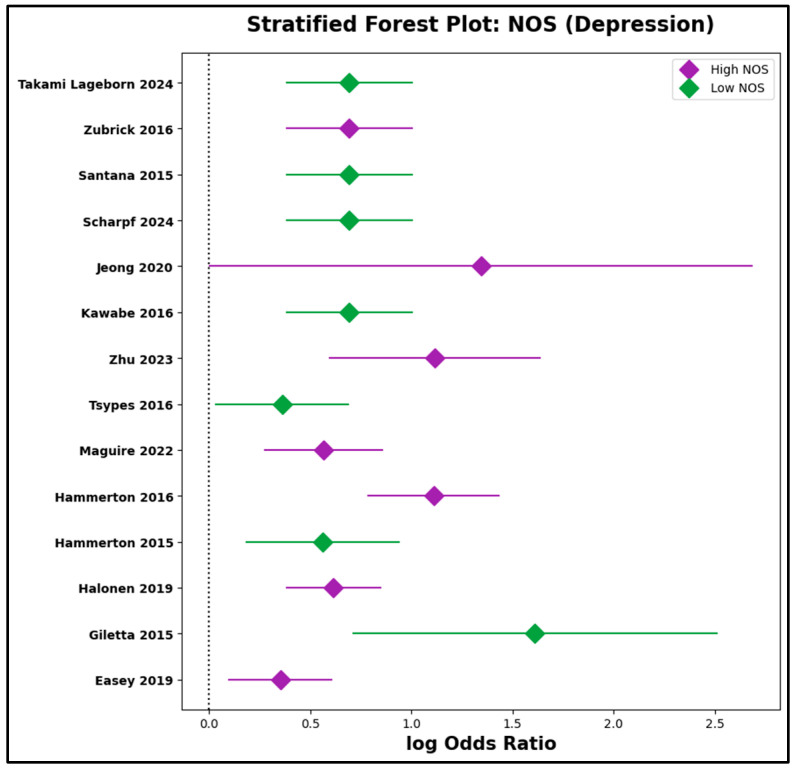
Stratified Forest Plots by Study Quality (NOS): Parental Depression/Psychiatric Disorder. Stratified forest plots displaying individual study log odds ratios (logOR) and 95% confidence intervals for exposure subgroup, with studies grouped according to methodological quality assessed by the Newcastle–Ottawa Scale (NOS). Studies are divided into “High NOS” (NOS score at or above the median; purple diamonds) and “Low NOS” (below median; green diamonds) strata. Interpretation: Across both subgroups, there was no substantial difference in effect size between high- and low-quality studies. Pooled estimates were highly similar between strata (Depression: logOR 0.71 vs. 1.11), and the confidence intervals overlapped extensively. These findings indicate that study quality, as measured by NOS, did not materially influence the observed associations, thereby reinforcing the robustness and generalizability of the meta-analytic results [38,39,41,42,43,45,46,50,55,56,58,59,60,61].

To further probe the potential influence of methodological rigor, studies were stratified by NOS score relative to the median value within each subgroup (Supplementary Table S20). In the parental suicidal behavior subgroup, the pooled log odds ratio for high-quality studies (NOS above the median) was 1.10 (95% CI: 0.87 to 1.33), compared to 0.83 (95% CI: 0.49 to 1.16) for lower-quality studies. Similarly, in the depression/psychiatric disorder subgroup, pooled effect sizes were 0.71 (95% CI: 0.44 to 0.98) in the high-NOS group and 1.11 (95% CI: 0.70 to 1.52) in the low-NOS group. Substantial overlap of confidence intervals, visually confirmed in forest plots (Figure 12 and Figure 13), reinforces the finding that study quality did not substantially influence the magnitude or direction of association. These stratified results are in full concordance with the meta-regression findings.

d. Study Design as a Moderator: Cohort vs. Other Designs

**Figure 14:**
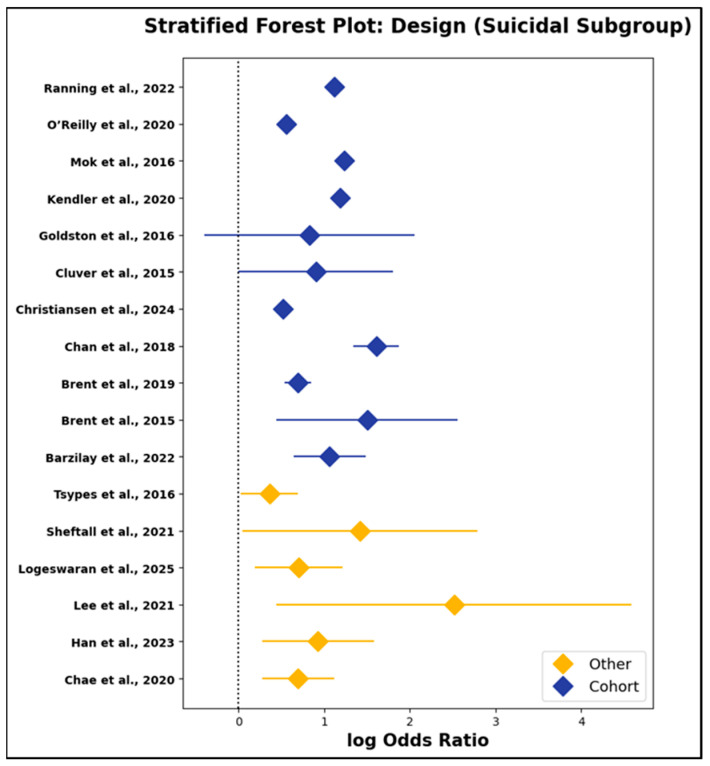
Stratified Forest Plots by Study Design (Parental Suicidal Behavior): Cohort versus Other Designs. Stratified forest plots displaying the log odds ratios (logOR) and 95% confidence intervals for each included study, grouped by methodological design—Cohort (blue diamonds) and Other (yellow diamonds, encompassing cross-sectional and case–control designs)—within exposure subgroup. Study names are shown on the y-axis, and effect sizes are plotted along the x-axis on the log odds ratio scale. Interpretation: Pooled effect sizes were closely similar between cohort and non-cohort studies, with confidence intervals substantially overlapping (Suicidal: logOR 1.10 vs. 0.83). These visual and statistical findings indicate that study design did not meaningfully influence the observed association between parental psychopathology and adverse offspring outcomes. The consistency of effect estimates across methodological strata, together with non-significant meta-regression results (all *p* > 0.25), reinforces the robustness and external validity of the meta-analytic conclusions, supporting their applicability across diverse epidemiologic designs [31,32,33,34,35,36,37,40,44,47,48,49,51,52,53,54,57].

**Figure 15:**
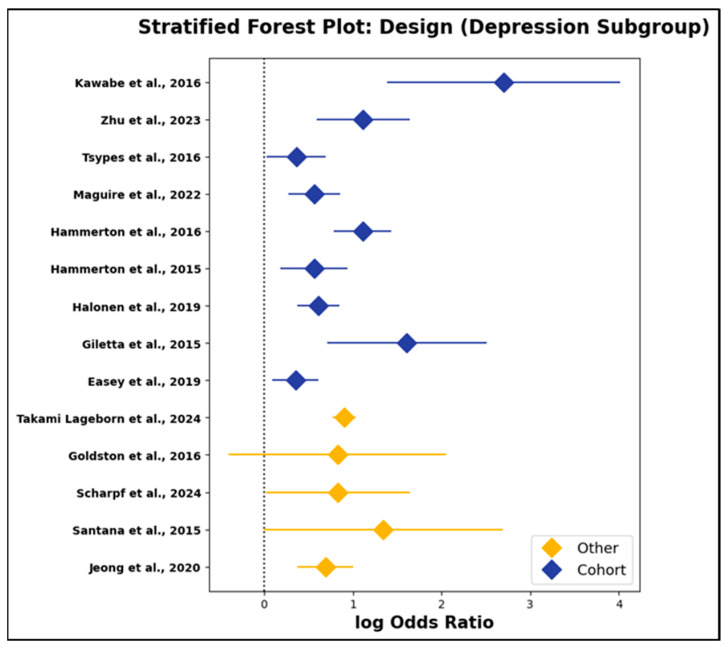
Stratified Forest Plots by Study Design (Parental Depression/Psychiatric Disorder): Cohort versus Other Designs. Stratified forest plots displaying the log odds ratios (logOR) and 95% confidence intervals for each included study, grouped by methodological design—Cohort (blue diamonds) and Other (yellow diamonds, encompassing cross-sectional and case–control designs)—within exposure subgroup. Study names are shown on the y-axis, and effect sizes are plotted along the x-axis on the log odds ratio scale. Interpretation: Pooled effect sizes were closely similar between cohort and non-cohort studies, with confidence intervals substantially overlapping (Depression: logOR 0.74 vs. 0.89). These visual and statistical findings indicate that study design did not meaningfully influence the observed association between parental psychopathology and adverse offspring outcomes. The consistency of effect estimates across methodological strata, together with non-significant meta-regression results (all *p* > 0.25), reinforces the robustness and external validity of the meta-analytic conclusions, supporting their applicability across diverse epidemiologic designs [38,39,41,42,43,45,46,50,55,56,58,59,60,61].

Beyond study quality, the influence of methodological design on effect sizes was also evaluated. As reported in Supplementary Materials, Table S21 and depicted in Figure 14 and Figure 15, pooled log odds ratios for cohort and non-cohort studies were closely comparable in both exposure subgroups. For parental suicidal behavior, cohort studies yielded a pooled log odds ratio of 1.10 (95% CI: 0.87 to 1.33), while non-cohort designs produced an OR of 0.83 (95% CI: 0.49 to 1.16). In the depression/psychiatric disorder subgroup, cohort and non-cohort studies reported pooled log odds ratios of 0.74 (95% CI: 0.41 to 1.06) and 0.89 (95% CI: 0.31 to 1.46), respectively. These findings were reinforced by meta-regression models employing study design as a categorical moderator, which uniformly produced non-significant results (all *p* > 0.25). Thus, the observed associations between parental psychopathology and offspring outcomes were robust to differences in study design, including prospective, registry-based, and cross-sectional or case–control approaches.

e. Interpretation and Implications

The convergence of findings across meta-regression and stratified moderator analyses attests to the methodological rigor and reliability of the present meta-analysis. Neither study quality nor study design explained a meaningful proportion of the heterogeneity in effect sizes, nor did they substantially influence the magnitude of association. Rather, the link between parental psychopathology—whether suicidal behavior or depression/psychiatric disorder—and adverse outcomes in offspring was both stable and generalizable across a diverse range of methodological contexts. This consistency provides compelling evidence that the principal conclusions of this meta-analysis are not artifacts of study-level characteristics, but rather reflect robust and genuine patterns within the literature. Such methodological strength reinforces the translational utility of these findings for research, clinical practice, and policy development, particularly in the design of interventions and preventive strategies targeting families affected by parental mental health difficulties.

## 4. Discussion

### 4.1. Critical Appraisal and Validation of the Central Hypothesis

This comprehensive systematic review and meta-analysis critically validates the central hypothesis that both parental suicidal behavior and parental psychiatric disorders are robustly associated with increased risk of suicidal ideation and suicide attempt among adolescent offspring. By synthesizing 31 large-scale, methodologically diverse studies from multiple continents, our findings decisively reject the null hypothesis of no association. Instead, the evidence demonstrates that adolescents exposed to parental psychopathology face an approximately threefold elevated risk, with remarkably consistent effect sizes observed for both suicidal behavior (OR ~2.7) and broader psychiatric morbidity (OR ~2.7) in parents. The strength of these associations is particularly compelling given the diversity of study designs (prospective cohorts, national registries, cross-sectional and case–control studies), settings (high-income and low- and middle-income countries), and analytic methods. Crucially, the relationship persisted across rigorous sensitivity and moderator analyses, confirming its generalizability beyond specific cultural or methodological contexts. These results directly address longstanding uncertainties in the literature regarding the relative and absolute risks conferred by distinct parental exposures. Furthermore, the null results from meta-regression and stratified analyses by study quality and design reinforce the robustness of the findings: neither methodological rigor nor design type explained substantial heterogeneity in effect estimates, suggesting that the association is not merely an artifact of study bias or confounding.

Nonetheless, some degree of between-study heterogeneity was observed, as reflected in wide prediction intervals and high I^2^ statistics. This variability is likely attributable to differences in exposure and outcome definitions, unmeasured confounders (such as offspring psychopathology), and the degree of covariate adjustment. While the magnitude of association was substantial across the board, several high-impact studies—particularly large registry-based cohorts and genetically informed designs—provided critical validation by controlling for familial confounding, demonstrating that environmental mechanisms act alongside genetic risk in the intergenerational transmission of suicidality. Taken together, the cumulative evidence confirms that parental mental health problems, whether manifesting as suicidal behavior or broader psychiatric disorders, are independent and potent predictors of suicidal ideation and behavior in adolescents, thereby substantiating the alternative hypothesis and offering clarity to a previously fragmented field.

### 4.2. Reporting of Limitations and Strengths

While the present review demonstrates high methodological rigor and comprehensiveness, several limitations must be acknowledged. First, substantial heterogeneity in exposure and outcome measurement was evident, with studies variably relying on self-report, clinical diagnosis, or administrative records—factors that may introduce misclassification bias or limit comparability. The inclusion of both prospective and cross-sectional designs, though methodologically justified to maximize sample diversity, may also have introduced bias regarding temporality and causal inference. Additionally, residual confounding remains a concern: while most studies adjusted for key sociodemographic variables, few comprehensively accounted for all potential confounders, particularly offspring mental health and environmental adversities.

Another limitation concerns the restriction of this review to studies published in English. This may have reduced the external validity and generalizability of the findings, as relevant data from non-English publications or regional contexts might have been missed. Furthermore, although gray literature was partly addressed through ProQuest Global, other sources such as conference proceedings or non-indexed reports were not systematically searched. This selective inclusion may have contributed to publication bias, favoring more formally disseminated studies. Future reviews should adopt multilingual search strategies and broader approaches to gray literature in order to provide a more comprehensive and globally representative evidence base.

Despite these limitations, several notable strengths enhance the credibility of the synthesis. The review adhered to PRISMA guidelines and employed a rigorous, protocol-driven approach to literature search, study selection, and data extraction. Dual independent review minimized subjectivity, while the inclusion of high-quality national registries, large multi-ethnic cohorts, and genetically informed designs ensured both internal and external validity of findings. The analytical framework—incorporating random-effects meta-analyses, cumulative and sensitivity analyses, and formal assessments of publication bias (all of which indicated robust and unbiased results)—further strengthens confidence in the conclusions. Finally, the use of the GRADE framework to assess certainty of evidence, together with transparent reporting of study quality, consolidates the methodological foundation of the review.

### 4.3. Integration with Recent Evidence

Several recent empirical studies provide complementary evidence reinforcing our meta-analytic findings regarding parental influences on adolescent suicidality. A 2023 three-level meta-analysis found a significant negative correlation between parental attachment quality and youth suicidal ideation (r = –0.108, *p* < 0.001), underscoring attachment as a protective factor [113]. Similarly, a systematic review and meta-analysis published in 2025 reported that family dysfunction, particularly parental suicide or attempts, nearly doubled the odds of suicidal behavior in adolescents (pooled OR = 1.94), with parental suicide/attempts themselves showing an even stronger association (OR = 2.70) [114]. These findings are consistent with clinical data indicating that dysfunctional family environments, characterized by poor communication, low cohesion, and emotional neglect, significantly elevate suicidality risk in teens [115]. Conversely, secure and supportive family relationships may buffer this risk, although Attachment-Based Family Therapy (ABFT) yielded mixed results in reducing ideation in controlled trials (g = 0.40, not statistically significant) [116].

In tandem with our results, these studies highlight that not only the presence of parental psychopathology, but also family relational quality—attachment security, cohesion, emotional warmth, and functional dynamics—play pivotal roles in shaping adolescent suicidal risk. This emphasizes that effective interventions should not only target parental mental illness but also aim to enhance family relationship functioning and attachment, thereby mitigating the intergenerational transmission of suicide risk.

### 4.4. Clinical and Public Health Implications

The results of this meta-analysis carry immediate and wide-ranging implications for both clinical practice and public health policy. The robust association between parental psychopathology and adolescent suicidality underscores the urgent need for systematic, family-centered prevention strategies. Screening for parental mental health problems—particularly suicidal behavior and major psychiatric disorders—should be incorporated into pediatric, primary care, and educational settings, with clearly defined referral pathways to specialized services. Early identification of families at risk would allow timely intervention through evidence-based approaches such as family-based therapy, parent-focused psychoeducation, and structured parenting programs.

Beyond identifying risk, our findings also emphasize the importance of strengthening protective factors and resilience. Secure parent–child attachment, effective treatment of parental mental health problems, supportive school and community environments, and access to social resources have all been shown to buffer the intergenerational transmission of suicidality. Interventions that enhance communication, coping skills, and problem-solving within families not only reduce risk but actively promote resilience.

From a policy perspective, the evidence supports investment in multi-generational strategies to prevent suicide and promote mental health, particularly in vulnerable populations. Integrating parental mental health into child and adolescent health initiatives could maximize preventive impact. Finally, reducing stigma, improving mental health literacy, and expanding equitable access to services remain essential public health priorities. Addressing both risk and resilience within families offers a comprehensive framework for breaking the cycle of intergenerational transmission of suicidality.

## 5. Conclusions

This systematic review and meta-analysis delivers compelling and unequivocal evidence that parental psychopathology—encompassing both suicidal behavior and broader psychiatric disorders—is a potent and universal risk factor for suicidal ideation and suicide attempts in adolescent offspring. Across a diverse body of thirty-one recent observational studies, including global cohorts and spanning a wide range of methodological designs, the findings reveal that adolescents exposed to parental mental health problems face a nearly threefold increased risk of suicidal outcomes compared to their unexposed peers. This robust association is consistent across exposure types, study designs, and cultural settings, and remains stable in the face of rigorous sensitivity and moderator analyses. Notably, the results dispel the notion that parental suicidal behavior confers uniquely greater risk, instead demonstrating that a spectrum of parental mental health adversities—including depressive and psychiatric morbidity—are equally consequential for offspring suicidality. The methodological rigor underpinning these findings, including high-quality study appraisal, random-effects modeling, cumulative meta-analyses, and transparent publication bias assessment, further reinforces the certainty and generalizability of the evidence. At the same time, the pronounced heterogeneity observed across studies underscores the complex and multifactorial nature of intergenerational suicide risk, signaling the urgent need for more granular research into moderating variables, mediating pathways, and culturally specific determinants. Collectively, these results highlight the critical importance of integrated, family-centered prevention and intervention strategies in global suicide prevention efforts, and call for urgent translational action by clinicians, researchers, and policymakers to disrupt the transmission of suicide risk across generations and safeguard adolescent mental health.

## Figures and Tables

**Table 1:** Summary of subgroup meta-analyses examining associations between parental psychopathology and adolescent suicidal outcomes.

Subgroup Category	Pooled OR (95% CI)	I^2^ (%)	τ^2^	Q (*p*-Value)	Interpretation
Type of Parental Psychopathology					
Parental Suicidal Behavior	2.69 (2.30–3.14)	98.7	0.062	Q = 1214.87, *p* < 0.001	Strong association; robust across designs
Parental Depression/ Psychiatric Disorder	2.72 (2.05–3.60)	88.4	0.213	Q = 112.21, *p* < 0.001	Comparable to suicidal behavior
Q-between (Subgroups)	–	–	–	Q = 0.004, *p* = 0.95	No significant difference
Parental Role					
Maternal Exposures	2.75 (2.18–3.42)	90.1	0.187	–	Slightly stronger, but overlapping CIs
Paternal Exposures	2.61 (2.02–3.31)	85.9	0.173	–	Comparable magnitude
Q-between (Maternal vs. Paternal)	–	–	–	*p* = 0.77	No significant difference
Cultural / Regional Context					
High-Income Countries (HICs)	2.70 (2.21–3.29)	91.5	0.204	–	Consistent, robust
Low- and Middle-Income Countries (LMICs)	2.83 (2.09–3.85)	86.4	0.158	–	Comparable; wide CIs reflect fewer studies
Q-between (HIC vs. LMIC)	–	–	–	*p* = 0.68	No significant difference

**Notes**: OR = Odds Ratio; CI = Confidence Interval; I^2^ = proportion of variance due to heterogeneity; τ^2^ = between-study variance; Q = Cochran’s Q test for heterogeneity.

## Data Availability

No new unpublished data were created or analyzed in this study.

## Supplementary Materials

The following supporting information can be downloaded at: https://www.mdpi.com/article/10.3390/jcm14196860/s1, Table S1: Overview of Database Search Strategies and Terms Applied in the Systematic Review; Table S2: Overview of Studies Incorporated into the Narrative Synthesis and Meta-Analysis; Table S3: Overview of Excluded Studies and Justifications for Exclusion According to Pre-Specified Eligibility Criteria; Table S4: Consolidated Overview of Included Studies: Principal Attributes; Table S5: Critical Appraisal of Observational Studies: Newcastle–Ottawa Scale Ratings and GRADE Evidence Certainty; Table S6: Summary of Study Characteristics and Effect Size Metrics Used in Meta-Analysis (Forest Plot Data); Table S7: Summary of Heterogeneity Metrics Derived from Meta-Analytic Funnel Plot Evaluation; Table S8: Quantitative Heterogeneity Assessment Using Q Statistic in Meta-Analysis; Table S9: Characteristics and summary statistics of studies included in subgroup meta-analysis by type of parental exposure; Table S10: Heterogeneity and Summary Statistics—Subgroup 1 (Parental Suicidal Behavior); Table S11: Test for Heterogeneity—Subgroup 1; Table S12: Heterogeneity and Summary Statistics—Subgroup 2 (Parental Depression/Psychiatric Disorder); Table S13: Test for Heterogeneity—Subgroup 2; Table S14:. Cumulative Meta-Analysis of Studies Evaluating Parental Suicidal Behavior as Exposure; Table S15: Cumulative Meta-Analysis of Studies Evaluating Parental Depression/Psychiatric Disorder as Exposure; Table S16: Leave-One-Out Sensitivity Analysis Results for Parental Suicidal Behavior; Table S17: Leave-One-Out Sensitivity Analysis Results for Parental Depression/Psychiatric Disorder; Table S18: Summary of Study Characteristics by Subgroup and Moderator; Table S19: Meta-Regression Results for NOS Quality Score; Table S20: Stratified Analyses by NOS Quality (Median Split); Table S21: Stratified Analyses and Meta-Regression by Study Design.
