# Supplementary material for: Parental Mental Health and Suicidal Behavior as Predictors of Adolescent Suicidal Ideation and Attempts: A Systematic Review and Meta-Analysis
Source: J Clin Med. 2025 Sep 28;14(19):6860. doi: 10.3390/jcm14196860 (PMC12525029; doi:10.3390/jcm14196860)
Supplement: Supplementary file 1 [file jcm-14-06860-s001.zip › jcm-3863425-supplementary.pdf]

Review

Parental Mental Health and Suicidal Behavior as Predictors of Adolescent Suicidal Ideation and Attempts: A Systematic Review and Meta−Analysis

SUPPLEMENTARY MATERIALS

Table S1. Overview of Database Search Strategies and Terms Applied in the Systematic Review.

Query	Platform	Database	Source Type	Search Terms and Filters
**1**	NCBI PubMed	MEDLINE (via PubMed)	Peer−reviewed biomedical studies	Combined MeSH and free−text search incorporating parental mental health (e.g., "parental mental health," "parental depression," "parental anxiety," "parental psychopathology," "parental bipolar disorder," "parental substance use," "parental schizophrenia"), parental suicidal behavior ("parental suicide," "parental suicide attempt," "parental suicide ideation"), and adolescent suicidality ("adolescent suicidal ideation," "adolescent suicide attempt," "adolescent suicidal behavior," "adolescent self−harm," "youth suicide"). Search was limited to studies published between 2015 and 2025, conducted in humans, in English, and designed as observational, cohort, cross−sectional, or population−based studies.
**2**	ClinicalTrials.gov	ClinicalTrials.gov Registry	Registry of clinical and population−based observational studies	Advanced search conducted using condition and study type fields. Keywords targeted parental mental health or suicidality in combination with adolescent suicidal ideation and behaviors. Study types were limited to observational, cohort, prospective, retrospective, and population−based research. Additional filters included English language, start date 2015–2025, and study status as ongoing or completed.
**3**	Cochrane Library	CENTRAL (Cochrane Central Register of Controlled Trials)	Systematic reviews, protocols, observational and cohort studies	Searches combined MeSH and keyword terms for parental mental health (as above), parental suicidal behavior, and adolescent suicidal outcomes. Studies were filtered for publication between 2015 and

Query	Platform	Database	Source Type	Search Terms and Filters
				2025, English language, studies in humans, and included observational, cohort, cross−sectional studies, and systematic reviews.
**4**	ProQuest Dissertations & Theses Global	ProQuest	Dissertations, theses, unpublished scholarly literature	Searches relied on keywords covering both parental mental health and adolescent suicidality (e.g., "parental mental health" AND "adolescent suicidal ideation", "parental psychopathology" AND "adolescent suicide attempt", "parental depression" AND "adolescent self−harm", "parental suicide attempt" AND "adolescent suicidal behavior"). Filters applied included English language, years 2015–2025, and document types restricted to dissertations, theses, or peer−reviewed journal articles.

References: Complete citations are included in the main reference list of the manuscript.

Table S2. Overview of Studies Incorporated into the Narrative Synthesis and Meta−Analysis.

No.	Study Identifier	Study Design	Included in Narrative Synthesis	Included in Meta−Analysis	Reason for Inclusion
**1**	Barzilay et al., 2022, USA	Prospective cohort study from the ABCD Study	Yes	Yes	The study met all eligibility criteria. Population: 5,214 non−related U.S. adolescents (ages 8.9–13.8 years) from African and European ancestries. Intervention: Observational study of genetic and familial risk factors. Comparison: Adolescents with vs. without parental

No.	Study Identifier	Study Design	Included in Narrative Synthesis	Included in Meta−Analysis	Reason for Inclusion
					history of suicide attempt/death. Outcomes: Self−reported suicide attempt assessed longitudinally over three study waves. Analytical approach involved stratified regression models and meta−analysis by ancestry.
**2**	Brent et al., 2015, USA	Prospective Longitudinal Cohort	Yes	Yes	The study met all eligibility criteria. Population: 701 offspring (mean age=17.7) of 334 parents with mood disorders, 57.2% with a history of suicide attempt. Intervention: Not applicable–observational study. Comparison: Offspring of suicide attempters vs non−attempters. Outcomes: Suicide attempt during follow−up, assessed via clinical interviews and standardized instruments. Analytical approach included multivariate logistic regression and structural path modeling.
**3**	Brent et al., 2019, USA	Pharmacoepidemiologic cohort study using claims data (MarketScan)	Yes	Yes	The study met all eligibility criteria. Population: 332,537 children aged 10–19 years linked to 242,612 parents (opioid users and non−users) in the United States, with follow−up from 2010 to 2016. Intervention: Parental prescription opioid use (>365 days). Comparison: Children of opioid−using parents vs. non−using parents. Outcomes: Suicide attempt identified via medical claims. Analyses included generalized estimating equations with logit link and multiple sensitivity analyses.
**4**	Chae et al., 2020, Korea	Cross−sectional national survey study (KNHANES 2007–2013, 2015)	Yes	Yes	The study met all eligibility criteria. Population: 2,324 adolescents aged 12–18 years and both parents. Intervention: Exposure to parental suicidal ideation (past 12 months). Comparison: Adolescents with vs. without parental suicidal ideation. Outcomes: Suicidal ideation in adolescents, depressive symptoms, stress level, and other covariates analyzed using multivariable logistic regression.
**5**	Chan et al., 2018, New Zealand	Cross−sectional national survey study (Youth’12)	Yes	Yes	The study met all eligibility criteria. Population: 8,500 high school students (ages 13–18) from 91 randomly selected schools across New Zealand. Intervention: Exposure to suicidal behavior (attempt or death) by family or friends. Comparison: Students exposed vs. not

No.	Study Identifier	Study Design	Included in Narrative Synthesis	Included in Meta−Analysis	Reason for Inclusion
					exposed to suicidal behavior. Outcomes: Suicide attempts and repeated non−suicidal self−injury (NSSI) in past 12 months.
**6**	Christiansen et al., 2024, Denmark	Nationwide registry−based cohort study (1983–1989 Danish birth cohorts, follow−up to age 35)	Yes	Yes	The study meets all eligibility criteria. Population: 384,569 Danish individuals born 1983–1989. Intervention: Observational study of parental suicide attempt and offspring mental illness (individually and combined) as predictors. Comparison: Offspring exposed to parental suicide attempt and/or own mental illness vs. unexposed. Outcomes: First suicide attempt and premature death in offspring, using registry linkage. Analytical approach: Cox regression and multistate modeling, with adjustment for confounders (sex, income, parental mental illness, age). Provides both relative (HR) and absolute risk estimates for suicide attempt and mortality.
**7**	Cluver et al., 2015, South Africa	Prospective longitudinal cohort study	Yes	Yes	The study met all eligibility criteria. Population: 3,515 adolescents aged 10–18 years from urban and rural households in Mpumalanga and Western Cape. Design: door−to−door household sampling with 1 adolescent per household. Exposure: cumulative Adverse Childhood Experiences (ACEs) including parental AIDS illness/death, homicide, abuse, domestic and community violence, food insecurity. Outcomes: past−month suicidal ideation, planning, and attempts assessed at 1−year follow−up using MINI−KID suicidality scale. Analysis: logistic regression and multiple mediation models using PROCESS macro. Mediation confirmed for mental health, not for substance misuse. High retention (96.8%).
**8**	Easey et al., 2019, UK	Population−based Longitudinal Study (ALSPAC Cohort)	Yes	Yes	The study met all eligibility criteria. Population: adolescents aged 15–16 years from the Avon Longitudinal Study of Parents and Children (ALSPAC), UK (n = 2,571, with complete data on exposures and outcomes). Exposure: birth order (firstborn, second−born, third+ born). Outcomes: suicide attempts (self−reported through adapted Child and Adolescent Self−harm in Europe questionnaires) and psychiatric disorders (Development and Well−being Assessment,

No.	Study Identifier	Study Design	Included in Narrative Synthesis	Included in Meta–Analysis	Reason for Inclusion
					DAWBA, using ICD–10 and DSM–IV criteria). Mediators: number of maternal depressive episodes (Edinburgh Postnatal Depression Scale, EPDS) and father absence (categorical: present, absent before age 5, absent age 5 or later). Statistical methods: multivariable logistic regression, mediation analyses using Mplus version 7, adjusted for confounders (maternal age, social class, income, gestational age, maternal alcohol and tobacco use in pregnancy). Results: higher birth order associated with increased risk of suicide attempts (OR = 1.42, 95% CI = 1.10–1.84) and psychiatric disorders (OR = 1.29, 95% CI = 0.99–1.69); mediation analyses showed small indirect effects via maternal depression and father absence, accounting for 8%–12% of the total association. The study reported robust findings suitable for inclusion in meta–analysis.
**9**	Giletta et al., 2015, China	Multiwave Prospective Longitudinal Study	Yes	Yes	The study met all eligibility criteria. Population: 565 tenth–grade adolescents (Mage = 16.03 years, SD = 0.52), 48.3% male, from urban (Changsha) and rural (Liuyang) schools in Hunan Province, China; 82.8% lived with both parents. Measures: Peer victimization (Revised Peer Experiences Questionnaire), depressive symptoms (CES–D), friendship characteristics (friendless, friends with NSSI, friends without NSSI), suicide ideation (Suicidal Ideation Questionnaire), nonsuicidal self–injury (Deliberate Self–Harm Inventory), suicide attempts (single binary item). Eight waves of data collection over two years. Statistical methods: Latent growth trajectory models, joint trajectory analyses, multinomial logistic regression, generalized estimating equations, Bayesian Information Criterion (BIC), Lo–Mendell–Rubin Ratio Likelihood Test. Results: High–high trajectory group (n = 52, 9.2%) showed ~5× greater risk of future suicide attempts (OR = 5.09, 95% CI = 1.19–21.82, p = .029); peer victimization significantly predicted membership in high–high trajectory (OR = 1.25, 95% CI = 1.02–1.52, p = .03).

No.	Study Identifier	Study Design	Included in Narrative Synthesis	Included in Meta−Analysis	Reason for Inclusion
**10**	Goldston et al., 2016, USA	Prospective Naturalistic Longitudinal Study with Bayesian Growth Mixture Modeling	Yes	Yes	The study met all eligibility criteria. Population: 180 adolescents (ages 12–18, 51% female, 80% European American) consecutively hospitalized for psychiatric reasons, recruited 1991–1995, followed on average 13.6 years (up to 19.3 years), total 2,273 assessments, avg. 12.6 assessments per person (range = 2–26). Measures: suicidal thoughts and behaviors (ordinal 1–5 scale, coded at each follow−up), psychiatric diagnoses (DSM−IV via ISCA, FISA), hopelessness (Beck Hopelessness Scale), trait anxiety (STAI), aggression (Aggression Questionnaire), impulsivity (BIS−11), history of abuse (sexual, physical), parental suicide attempt, social support (Social Support Questionnaire), coping beliefs (Reasons for Living Inventory), adult functioning (SAS−SR, CAFAS). Results: Bayesian mixture models identified four STB trajectory classes—Increasing Risk (11%, n = 20), Highest Overall Risk (12%, n = 22), Decreasing Risk (33%, n = 60), Low Risk (44%, n = 78). Highest Overall Risk class: most severe STBs over time, highest MDD/GAD duration, hopelessness, anxiety, sexual abuse; Increasing Risk class: elevated adult aggression (b = 6.23, SE = 2.88, p < .05), impulsivity (b = 10.02, SE = 3.29, p < .01), role performance impairment (b = 1.08, SE = 0.52, p < .05), behavioral appropriateness impairment (b = 0.94, SE = 0.43, p < .05); protective factors: coping beliefs (b = –0.79, SE = 0.25, p < .01), social support.
**11**	Halonen et al., 2019, Finland	Nationwide Population−Based Prospective Cohort Study with Counterfactual Mediation Analysis	Yes	Yes	The study met all eligibility criteria. Population: 52,182 individuals from the 1987 Finnish Birth Cohort (50.9% male, 49.1% female), followed until age 28. Exposure: parental mental disorders (12%); identified through inpatient/outpatient psychiatric care, psychiatric disability pension, diagnoses ICD−9 291–319, ICD−10 F10–F99. Outcomes: offspring psychiatric work disability between ages 19–28 due to depressive/anxiety disorders (ICD−10 F32–34, F40–48); n = 446 (0.9%). Mediators: adolescent mental disorders (n = 4,094, 8%), adolescent social disadvantage (n = 14,052, 27%; defined by low

No.	Study Identifier	Study Design	Included in Narrative Synthesis	Included in Meta−Analysis	Reason for Inclusion
					parental education, social assistance, low GPA, or parental unemployment). Total effect: OR = 1.85 (95% CI = 1.46–2.34); mediated by adolescent mental disorders: OR = 1.19 (95% CI = 1.11–1.28, 35% mediation); mediated by social disadvantage: OR = 1.15 (95% CI = 1.06–1.24, 28% mediation). Sex−specific mediation: mental disorders: women 55%, men 22%; social disadvantage: women 65%, men 11%. Region−specific effects stronger in Southern/Western Finland (OR = 2.14, 95% CI = 1.61–2.84) compared to Northern/Eastern (OR = 1.36, 95% CI = 1.00–1.85). Sensitivity analysis using educational attainment at age 21 consistent with main findings.
**12**	Hammerton et al., 2015, UK	Population−Based Longitudinal Cohort Study with Structural Equation Modeling (ALSPAC)	Yes	Yes	The study met all eligibility criteria. Population: 10,559 mother−child pairs from ALSPAC; 4,588 offspring provided data on suicidal ideation and suicide attempt at age 16 (50% female, 96% White). Maternal depression measured across 10 time points (antenatal to age 11) using the Edinburgh Postnatal Depression Scale; latent classes: minimal (40%, n ≈ 4,224), moderate (55%, n ≈ 5,807), chronic−severe (5%, n ≈ 528). Offspring suicidal ideation at age 16: 15.3% (95% CI: 14–17%); lifetime suicide attempt: 7.7% (95% CI: 7–9%). Psychopathology mediators (measured at age 15): MDD (15.4%, SD = 2.7 symptoms), GAD (11.3%, SD = 2.3), DBD (9.8%, SD = 2.1), ADHD (8.1%, SD = 1.9), alcohol abuse (6.5%, SD = 2.0). Multiple imputation sample (n = 10,559) showed direct effect of chronic maternal depression on offspring ideation: B = 0.36, 95% CI: 0.17–0.55, $p < .001$; indirect effects: MDD B = 0.10 (95% CI: 0.06–0.15), GAD B = 0.06 (95% CI: 0.03–0.09), DBD B = 0.11 (95% CI: 0.06–0.16), ADHD B = –0.02 (95% CI: –0.06–0.02), alcohol abuse B = 0.02 (95% CI: 0.00–0.04). For suicide attempt, direct effect: B = 0.31, 95% CI: 0.10–0.52, $p = .003$; indirect effects: MDD B = 0.11 (95% CI: 0.06–0.16), GAD B = 0.07 (95% CI: 0.03–0.11), DBD B = 0.11 (95% CI: 0.06–0.16), ADHD B = 0.06 (95% CI: 0.01–0.12), alcohol abuse B = 0.02 (95% CI: –0.00–0.03). Wald χ^2 tests (df = 1): MDD vs. ADHD χ^2 = 7.21,

No.	Study Identifier	Study Design	Included in Narrative Synthesis	Included in Meta−Analysis	Reason for Inclusion
					p = .007; MDD vs. alcohol abuse $\chi^2 = 9.84$, p = .002. Sensitivity analyses (complete case, n = 2,445) yielded slightly attenuated indirect effects but consistent directions.
**13**	Hammerton et al., 2016, UK	Population−Based Longitudinal Cohort Study with Structural Equation Modeling (ALSPAC)	Yes	Yes	The study met all eligibility criteria. Population: 10,559 mother−offspring pairs from ALSPAC, UK; suicidal ideation data at age 16 available for 4,588 adolescents (15% past−year ideation, 95% CI: 14–17%; 11% males, 20% females); 2% of mothers reported suicide attempts during child's first 11 years. Maternal depression trajectories across 10 waves (antenatal to age 11) categorized as minimal (40%), moderate (55%), chronic−severe (5%) using Edinburgh Postnatal Depression Scale. Mediators: maternal suicide attempt, offspring psychiatric disorder at age 15 (9%, any DSM−IV/ICD−10 disorder via DAWBA), parent−child relationship quality (age 9; mean score minimal: 2.94, moderate: 3.46, chronic−severe: 4.15). Chronic−severe depression vs. minimal: offspring ideation OR = 3.04 (95% CI: 2.19–4.21), offspring disorder OR = 5.51 (95% CI: 3.92–7.74), maternal suicide attempt OR = 36.26 (95% CI: 20.12–65.37), parent−child relationship $\beta = 0.26$ (95% CI: 0.15–0.37), p < .001. Total effect B = 0.71 (95% CI: 0.49–0.93); 46% mediated via maternal suicide attempt, 45% via offspring disorder, 6% via parent−child relationship, 1% via both parent−child relationship and offspring disorder. Adjusted indirect effects: maternal suicide attempt B = 0.26 (95% CI: 0.07–0.45), offspring disorder B = 0.27 (95% CI: 0.12–0.42), parent−child relationship B = 0.04 (95% CI: 0.02–0.07), both B = 0.01 (95% CI: 0.004–0.02). Gender comparison: stronger mediation via offspring disorder in females (p = .015).
**14**	Han et al., 2023, Korea	Case−Control Study with Propensity Score Matching (PSM) and	Yes	Yes	The study met all eligibility criteria. Population: weighted national sample ~3,911,090 Korean adolescents aged 12–18; analytic sample n = 6,512 (SI group n = 428, weighted ~261,645; MC group n = 421, weighted ~243,622); within SI, HS group n = 69, weighted ~36,934;

No.	Study Identifier	Study Design	Included in Narrative Synthesis	Included in Meta−Analysis	Reason for Inclusion
		Complex Sample Logistic Regression			N−HS group n = 331, weighted ~224,711. Maternal factors (raw n, weighted %): diagnosed depression 70 (2.3%), depressive mood 107 (3.4%), suicidal ideation 46 (1.5%), alcohol use 242 (7.5%), help−seeking 27 (0.9%). Adolescent factors: depressive mood 1,869 (28.7%), smoking 1,000 (15.3%), help−seeking 1,200 (18.4%). Model 3 results (full adjustment): SI vs MC — maternal diagnosed depression OR 2.109 (95% CI 1.023–4.350, p = .043), maternal depressive mood OR 2.155 (1.224–3.793, p = .008), maternal suicidal ideation OR 2.532 (1.322–4.851, p = .005); adolescent depressive mood OR 6.759 (4.315–10.586, p < .001), adolescent smoking OR 3.501 (1.859–6.593, p < .001), adolescent help−seeking OR 4.847 (2.547–9.222, p < .001). HS vs N−HS (within SI group): maternal depressive mood OR 4.486 (1.312–15.34, p = .017), maternal suicidal ideation OR 0.150 (0.031–0.721, p = .018), maternal help−seeking OR 17.495 (1.812–168.877, p = .014); adolescent depressive mood OR 9.417 (2.775–31.944, p < .001), adolescent smoking OR 3.772 (1.125–12.648, p = .031). Interaction effects: maternal depressive mood stronger for HS subgroup (OR 2.911, 95% CI 1.473–5.755); maternal suicidal ideation negative effect on adolescent help−seeking (OR 0.150, 95% CI 0.031–0.721).
**15**	Jeong et al., 2020, Korea	Cross−sectional, retrospective secondary data analysis using 11 years of national survey data (KYRBS), stratified multistage clustered sampling, complex sample weighting, and multivariate logistic regression.	Yes	Yes	The study met all eligibility criteria. Population: 788,411 Korean adolescents (aged 13–18), surveyed between 2007–2017. Data source: Korean Youth Risk Behavior Web−based Survey (KYRBS). Exposures: demographic, dietary, behavioral, and psychological factors, including depression, stress, happiness, smoking, alcohol use, breakfast habits, academic achievement, residential type. Outcomes: suicidal ideation and suicide attempts in the past 12 months. Statistical models provided adjusted odds ratios (ORs) and 95% confidence intervals (CIs), suitable for meta−analytic inclusion.

No.	Study Identifier	Study Design	Included in Narrative Synthesis	Included in Meta−Analysis	Reason for Inclusion
**16**	Kawabe et al., 2016, Japan	Population−based cross−sectional survey of junior high school students (aged 12–15) and their caregivers in Kumakogen Town, Japan; mental health assessed using GHQ−30 and POMS; multivariate logistic regression analysis to identify predictors of adolescent suicidal ideation, focusing on caregiver mental health.	Yes	Yes	The study met all eligibility criteria. Population: 185 junior high school students and their caregivers, Japan. Instruments: General Health Questionnaire (GHQ−30), Profile of Mood States (POMS). Outcome: Suicidal ideation assessed via GHQ item 30 (>2 points). Key finding: caregiver suicidal depression (GHQ subscale) had the highest adjusted odds ratio (OR) for predicting adolescent suicidal ideation. Statistical results included adjusted odds ratios and confidence intervals, making the study suitable for inclusion in meta−analytic synthesis.
**17**	Kendler et al., 2020, Sweden	Population−wide register−based observational cohort study using an extended adoption design across four family types (intact families, not−lived−with father families, stepfather families, adoptive families), assessing genetic and rearing contributions to intergenerational transmission of suicide attempt and suicide	Yes	Yes	The study met all eligibility criteria. Population: Swedish national birth cohorts (offspring born 1960–1990), covering 2,417,104 offspring and their parents. Data sources: Swedish Multi−Generation Register, Population and Housing Censuses, National Patient Register, Outpatient Care Register, Mortality Register. Outcomes: suicide attempt and suicide death identified via ICD codes. The study utilized tetrachoric correlations and logistic regression to estimate parent−child transmission across genetic and rearing pathways, with effect sizes and confidence intervals suitable for meta−analytic synthesis.

No.	Study Identifier	Study Design	Included in Narrative Synthesis	Included in Meta−Analysis	Reason for Inclusion
		death via tetrachoric correlations and logistic regression.			
**18**	Lee et al., 2021, Korea	Cross−sectional, population−based study using data from 2015–2018 Korean National Health and Nutrition Examination Survey (KNHANES); hierarchical logistic regression to assess parental factors associated with suicide attempts among adolescent girls.	Yes	Yes	The study met all eligibility criteria. Population: 890 adolescent girls aged 12–18 years and their 1,500 parents (645 fathers, 855 mothers) from South Korea. Data source: KNHANES. Outcome: adolescent girls' suicide attempts in the past year. Exposure: parental sociodemographic and mental health factors including maternal and paternal suicidal ideation, plans, and attempts. Statistical model: hierarchical logistic regression with adjusted odds ratios (OR) and 95% confidence intervals (CI), suitable for meta−analysis.
**19**	Logeswaran et al., 2025, Denmark	Self−controlled case series (SCCS), population−based registry study	Yes	Yes	The study met all eligibility criteria. Population: National Danish registry cohort of offspring bereaved by parental suicide (n=188 with self−harm/suicide outcome) and parental non−suicide death (n=734), observed from 1980–2016. Exposure: 2−year period centered on reaching the age at which the parent died by suicide, compared to 15−year flanking periods. Comparison: Offspring bereaved by suicide vs. by other causes. Outcomes: Medically severe self−harm or suicide (secondary−care contact or death). Analytical approach: Fixed−effects conditional Poisson regression in SCCS design, adjusted for age, marital status, and income. Study provides effect estimates (adjusted IRR) for the association between parental suicide and offspring suicidality at a critical age, fulfilling criteria for both narrative synthesis and meta−analysis.

No.	Study Identifier	Study Design	Included in Narrative Synthesis	Included in Meta−Analysis	Reason for Inclusion
**20**	Maguire et al., 2022, Northern Ireland	Population−wide data−linkage cohort study linking 2011 Northern Ireland Census to mortality records (2011–2016); logistic regression models estimating association between parental poor mental health (MH) and offspring MH and suicide risk; stratified analyses by offspring age.	Yes	Yes	The study met all eligibility criteria. Population: 618,970 offspring living with parents in Northern Ireland at the 2011 Census. Exposure: parent(s) self−reporting poor MH (emotional, psychological, or MH condition ≥12 months). Outcomes: offspring poor MH and suicide death. Analytic models: logistic regression reporting adjusted odds ratios (OR) with 95% confidence intervals (CI), accounting for clustering within households. Study suitable for meta−analytic inclusion.
**21**	Mok et al., 2016, Denmark	Population−based cohort study	Yes	Yes	The study met all eligibility criteria. Population: 1,743,525 individuals born in Denmark (1967–1997), followed from age 15 until first adverse outcome, death, emigration, or December 31, 2012. Intervention: Not applicable – observational study of parental psychiatric disorders, including suicide attempt, as risk factors. Comparison: Offspring with vs. without parental psychiatric diagnosis (including suicide attempt). Outcomes: First suicide attempt in offspring, ascertained via national registry linkage; reported as incidence rate ratios (IRRs) stratified by parent diagnosis, sex, and whether one or both parents affected. Analytical approach involved log−linear Poisson regression models with adjustment for offspring age, sex, calendar year, socioeconomic status, and relevant interactions. The study provides robust population−level evidence for the elevated risk of suicide attempt in offspring of parents with a range of psychiatric disorders, especially antisocial personality disorder, cannabis misuse, and suicide attempt.

No.	Study Identifier	Study Design	Included in Narrative Synthesis	Included in Meta−Analysis	Reason for Inclusion
**22**	O'Reilly et al., 2020, Sweden	Nationwide population cohort; genetically informed offspring−of−siblings design	Yes	Yes	Study met all inclusion criteria. Population: 2,762,883 Swedish offspring (born 1973–2001), age ≥12, both sexes, quasi−experimental cousin comparison. Exposure: Parental suicidal behavior before age 18. Outcomes: Offspring suicide attempt or death. Analytical methods: Structural equation modeling for heritability and environmental effect, Cox fixed−effects for cousin pairs. Adjusted for major psychiatric comorbidities and demographic covariates. Shows robust independent intergenerational association.
**23**	Ortin−Peralta et al., 2024, USA	Longitudinal Cohort Study (ABCD Study)	Yes	Yes	The study met all eligibility criteria. Population: 9,194 children aged 9−10 from the Adolescent Brain Cognitive Development (ABCD) study, followed for 2 years. Exposure: parental suicide attempt or suicide death reported by caregiver at baseline. Outcome: suicidal ideation and suicide attempt at baseline and first−time endorsement at follow−up, measured with K−SADS−PL DSM−5. The study used logistic regression, structural equation modeling, and mediation models. Coefficients reported include adjusted odds ratios, confidence intervals, and mediation indirect effects.
**24**	Ranning et al., 2022, Denmark	Prospective Cohort Study	Yes	Yes	The study met all eligibility criteria. Population: 4,419,651 individuals aged ≥10 years followed from 1980–2016. Exposure: parental suicide attempt registered in national health registers. Outcome: child's first suicide attempt measured via hospitalization records, analyzed separately for adolescent age (13–17 years). Analyses used Poisson regression and Cox proportional hazards models adjusted for demographic, socioeconomic, and psychiatric covariates. Coefficients reported included incidence rate ratios, confidence intervals, and cumulative hazards.
**25**	Santana et al., 2015, Brazil	Cross−sectional Retrospective Study (Sao Paulo Megacity Mental Health Survey)	Yes	Yes	The study met all eligibility criteria. Population: n = 2,942 adults reporting on parental psychopathology and own lifetime suicidal behaviors. Exposure: parental depression, panic disorder, GAD, substance abuse, antisocial personality. Outcome: suicidal ideation

No.	Study Identifier	Study Design	Included in Narrative Synthesis	Included in Meta−Analysis	Reason for Inclusion
					and attempt reported separately for adolescence (13–19 years). Survival models with ORs adjusted for demographics and offspring psychopathology.
**26**	Scharpf et al., 2024, Tanzania	Cross−sectional Study (Community−based in 3 refugee camps)	Yes	Yes	The study met all eligibility criteria. Population: n = 230 families (children aged 7–15, parents). Exposure: parental psychiatric disorders, parental suicidal behavior. Outcome: suicidal ideation, suicide plans, suicide attempts measured separately for children and parents using MINI−KID and MINI. Multinomial logistic regression provided adjusted odds ratios with confidence intervals for adolescent suicide risk.
**27**	Sheftall et al., 2021, USA	Cross−sectional, case−control study	Yes	Yes	The study met all eligibility criteria. Population: 146 U.S. children (ages 6–9), oversampled for parental suicide attempt (PH+). Exposure: Parental history of suicide attempt (self−report). Comparison: Children with (PH+) vs. without (PH−) parental suicide attempt history. Outcome: Child self−reported suicidal ideation (past month). Analytical approach: Multivariate logistic regression controlling for child psychiatric symptoms. Included because it directly assessed the association between parental suicide attempt and child suicidal ideation using standardized outcome measures.
**28**	Takami Lageborn et al., 2024, Sweden	Population−based Cohort Study (Swedish National Registers).	Yes	Yes	The study met all eligibility criteria. Population: n = 24,788 offspring of one parent with bipolar disorder; n = 247,880 matched controls. Exposure: parental bipolar disorder (type 1/type 2), parental psychiatric comorbidity, parental sex. Outcome: suicide attempt, suicide before age 18. Statistical methods: Cox proportional hazards models adjusted for parental education, psychiatric comorbidity, and parental suicide attempt. Stratified by bipolar subtype, maternal/paternal exposure, one vs. two parents affected.
**29**	Tsypes et al., 2016, USA	Prospective Longitudinal Study	Yes	Yes	The study met all eligibility criteria. Population: 209 mother−child pairs (children aged 8–14 years; mothers with vs. without history of MDD). Exposure: maternal depression, maternal suicide attempt.

No.	Study Identifier	Study Design	Included in Narrative Synthesis	Included in Meta−Analysis	Reason for Inclusion
					Outcome: child suicidal ideation (baseline & new onset over 2−year follow−up), assessed via K−SADS−PL. Predictors: cognitive vulnerabilities (hopelessness, global self−worth, brooding rumination).
30	Zhu et al., 2023, UK	Prospective, longitudinal population−based cohort (MCS, UK)	Yes	Yes	Studiul îndeplinește toate criteriile de eligibilitate. Populație: 12.520 adolescenți urmăriți 14 ani, cohortă reprezentativă național UK. Expunere: traiectorii longitudinale de distress psihologic parental și probleme internalizante/externalizante la copil. Comparație: 4 grupuri LCGA. Outcome: autovătămare și tentativă de suicid auto−raportate la adolescenți. Analiză: modelare traiectorii LCGA, BCH, Wald test, ajustare date lipsă. Importanță: demonstrează rolul combinat al sănătății mentale parentale și problemelor la copil în predicția comportamentului suicidar adolescentin, susținând intervenții “two−generation” adaptate.
31	Zubrick et al., 2016, Australia	National cross−sectional, population−based survey	Yes	Yes	The study met all eligibility criteria. Population: Australian adolescents aged 12–17 years, nationally representative sample (n = 2,653). Exposure: parental and adolescent mental disorders, family context. Comparison: adolescents with vs. without mental disorders. Outcomes: suicidal ideation, suicide plan, suicide attempt (12−month prevalence and risk estimates). Robust survey methodology, weighted national estimates, and multivariate logistic regression. Directly relevant for both qualitative synthesis and quantitative meta−analysis.

References: Complete citations are included in the main reference list of the manuscript.

Table S3. Overview of Excluded Studies and Justifications for Exclusion According to Pre−Specified Eligibility Criteria.

No.	Authors, Year, Country	Study Type	Inclusion Status	Exclusion Justification

1	Alvarez–Subiela et al., 2022, Spain	Case–Control Study	Excluded	While family factors (including parental mental health and suicidal behavior) were assessed, the study did not directly and quantitatively analyze parental psychiatric disorder or suicidal behavior as primary predictors of adolescent suicidal ideation or behavior. The main analytical focus is on family attachment/bonding, functioning, and stressful life events. No specific risk estimates (OR/RR) for parental psychiatric disorder as an isolated predictor are reported.
2	Boyda et al., 2018, UK	Cross–sectional analysis using data from the National Comorbidity Survey Replication (NCS–R)	Excluded	The study assessed the mediating role of adult attachment styles between parental psychopathology and suicidality, but the sample consisted exclusively of adults aged 18+. It did not examine adolescent suicidal ideation or behavior. As such, it did not meet the inclusion criteria, which specify adolescent populations as the focus.
**3**	Calderaro et al., 2022, Germany	Systematic review and meta–analysis of population–based studies	Excluded	This study is a systematic review and meta–analysis, not a primary research study. According to the predefined inclusion criteria, only original, primary studies (e.g., cohort, case–control, cross–sectional) reporting associations between parental mental health/suicidal behavior and adolescent suicidal ideation/behavior are eligible. Meta–analyses and systematic reviews are not included unless the review is explicitly designed as an umbrella review.
**4**	Carballo et al., 2020, Spain	Systematic Review	Excluded	This is a systematic review of 44 studies on psychosocial risk factors for suicidality in children and adolescents. Only primary studies are eligible for inclusion in our review and meta–analysis. Systematic reviews and meta–analyses are excluded unless the current review is of umbrella type, which is not the case.
**5**	Goodday et al., 2019, Canada	Systematic Review	Excluded	Systematic review of existing literature; does not report primary or aggregate data suitable for inclusion in a systematic review/meta–analysis of primary studies; not an umbrella review.
**6**	Kushal et al., 2020, Bangladesh	Cross–sectional, secondary analysis of GSHS, 52 countries	Excluded	Did not directly assess parental psychiatric disorders or parental suicidal behavior as predictors of adolescent suicidal ideation or behavior; focus was on the quality of the parent–adolescent relationship.
**7**	Miklowitz et al., 2020, USA	Randomized Controlled Trial	Excluded	Although parental psychiatric disorder was present (≥1 first– or second–degree relative with bipolar I/II disorder), the study did not directly assess psychiatric disorders in parents as predictors of adolescent suicidal ideation or behavior. The study evaluated the efficacy of Family–Focused

				Therapy versus Enhanced Care in preventing mood episode recurrence and conversion to bipolar disorder, with no analysis focused on suicidal ideation or behavior. Therefore, it did not meet the inclusion criteria, which specified direct assessment of parental psychiatric disorders predicting adolescent suicidal ideation or behavior.
**8**	Rukundo et al., 2018, Uganda	Protocol for a systematic review (not an original study or completed review)	Excluded	The article is a protocol only, not an actual systematic review or meta–analysis with extracted or pooled results. No original data, effect estimates, or analysis provided. Not eligible for inclusion unless conducting an umbrella review.
**9**	Sanchez–Gistau et al., 2015, Spain	Controlled Comparative Cohort Study	Excluded	Although the study assessed psychiatric disorders in adolescent offspring of parents with schizophrenia or bipolar disorder, it did not directly measure adolescent suicidal ideation or suicidal behavior. The outcomes were limited to DSM–IV Axis I psychiatric diagnoses without specific assessment of suicidality. Therefore, it did not meet the inclusion criteria, which specified direct measurement of adolescent suicidal ideation or behavior.
**10**	Thorup et al., 2018, Denmark	Population–based Register Cohort Study	Excluded	The study investigated incidence of any child and adolescent mental disorder (age 0–17) in offspring of parents with severe mental illness (schizophrenia, bipolar disorder, major depression) but did not assess adolescent suicidal ideation or behavior as an outcome. Therefore, it did not meet inclusion criteria focusing on adolescent suicidal ideation or behavior as primary outcome.
**11**	Zamora–Kapoor et al., 2016, USA	Retrospective Cohort Study (Add Health dataset)	Excluded	The study examined exposure to suicide attempt among family members and friends as predictors of adolescent suicidal ideation, but did not directly assess parental psychiatric disorders or parental suicidal behavior as predictors. Therefore, it did not meet the inclusion criteria requiring parental–specific predictors.

References: Complete citations are included in the main reference list of the manuscript.

Table S4. Consolidated Overview of Included Studies: Principal Attributes.

No.	Citation	Study Type	Participant Details	Exposure / Intervention Assessed	Group Allocation	Outcomes Evaluated	Analysis Approach	Major Findings
**1**	Barzilay et al., 2022, USA	Prospective cohort study (ABCD Study)	Adolescents (n = 5,214) aged 8.9–13.8 years (European and African ancestry).	Observational – Genetic risk and parental history for suicide attempts	Adolescents with vs. without parental suicide attempt/death history	Primary Outcomes (meta−analyzed sample):<br>Parental suicide attempt/death: OR = 2.88, 95% CI [1.87, 4.42], $p < .001$;<br>Suicide attempt PRS: OR = 1.29, 95% CI [1.10, 1.52], p = .002;<br>Female sex: OR = 1.12, 95% CI [0.83, 1.52], p = .454 (not significant);<br>Stratified by ancestry:<br>European ancestry: Addition of PRS−SA to parental history model improved Nagelkerke's R^2 from 1.9% to 2.6% (ΔR^2 = 0.7%, p = .009);<br>African ancestry: Addition of PRS−SA model did not significantly improve prediction (ΔR^2 not significant).	Binary logistic regression; Stratified analysis by ancestry; Meta−analysis combining ancestry−specifi c models; Nagelkerke's R^2 for model comparison; Permutation testing for PRS threshold selection.	Parental history of suicide attempt/death strongly predicts adolescent suicide attempt. PRS−SA offers a small but statistically significant improvement in prediction in European ancestry youth. Integration of genetic risk scores may augment suicide risk classification efforts, but requires further validation, particularly in African ancestry populations.
**2**	Brent et al., 2015, USA	Longitudinal cohort study (mean follow−up 5.6 years)	Offspring (n = 701) of parents (n = 334) with mood disorders (57.2% with parental suicide. attempt history); mean age 17.7 years.	Observational – familial transmission of suicide risk model.	Offspring of suicide attempters vs. non−attempters.	Primary Outcomes:<br>Proband lifetime suicide attempt: OR = 4.79 (95% CI [1.75, 13.07], p = .002);<br>Offspring baseline mood disorder: OR = 4.20 (95% CI [1.37, 12.86], p = .01);<br>Prior suicide attempt in	Multivariate logistic regression (backward stepwise); Cox proportional hazards modeling; Path	Parental suicide attempt increases offspring suicide attempt risk nearly fivefold. Mood disorders and impulsive aggression are additional significant predictors. Early intervention

No.	Citation	Study Type	Participant Details	Exposure / Intervention Assessed	Group Allocation	Outcomes Evaluated	Analysis Approach	Major Findings
						offspring: OR = 5.69 (95% CI [1.94, 16.74], p = .002);<br>Mood disorder at proximal time point: OR = 11.32 (95% CI [2.29, 56.00], p = .004).<br>Secondary Findings:<br>Cox regression: Parental attempt predicting offspring attempt: HR = 4.13 (95% CI [1.45, 11.77], p = .008);<br>Path analysis: Proband attempt → offspring attempt, standardized β = 0.52 (SE = 0.20, p = .008).	analysis (Mplus 7.11); Multiple imputation (STATA ICE); False Discovery Rate (FDR) correction (Benjamini–Yekutieli procedure).	targeting emotional regulation and impulsivity could reduce future suicide attempts in high−risk adolescents.
**3**	Brent et al., 2019, USA	Pharmacoepidemiologic cohort study (MarketScan, 2010–2016)	Children (n= 332,537) aged 10–19 years linked to parents (n= 242,612).	Observational – Parental long−term prescription opioid use (>365 days).	Children of opioid−using parents vs. children of non−using parents.	Primary Analysis:<br>Suicide attempt: OR = 1.99, 95% CI [1.71, 2.33], p < .001.<br>Adjusted for child age and sex: OR = 1.85, 95% CI [1.58, 2.17], p < .001.<br>Adjusted for psychiatric comorbidities (parental depression, SUD):<br>OR = 1.46, 95% CI [1.24, 1.72], p < .001.<br>Adjusted additionally for parental history of suicide attempt:<br>OR = 1.45, 95% CI [1.23, 1.71], p < .001.	Propensity Score Matching (greedy matching, caliper 0.2 SD); Generalized Estimating Equations (GEE) with logit link; Adjustments for demographic and psychiatric variables; Sensitivity analyses for overdose	Long−term parental opioid use is associated with a near twofold increase in risk of suicide attempts among offspring. This association persists after controlling for multiple demographic and psychiatric confounders. Intervention strategies should prioritize suicide risk screening in families affected by chronic opioid exposure.

No.	Citation	Study Type	Participant Details	Exposure / Intervention Assessed	Group Allocation	Outcomes Evaluated	Analysis Approach	Major Findings
						Sensitivity Analyses: Geographic adjustment (high opioid use areas): OR = 2.00, 95% CI [1.71, 2.34], p < .001. Excluding overdose−related suicide attempts: OR = 2.02, 95% CI [1.64, 2.46], p < .001. One parent opioid use vs. none: OR = 1.44, 95% CI [1.24, 1.71], p < .001. Both parents opioid use vs. none: OR = 1.51, 95% CI [1.12, 2.04], p = .007.	exclusion and geographical variations; Use of ICD−9 and ICD−10 diagnostic codes for suicide attempt ascertainment.	
**4**	Chae et al., 2020, Korea	Cross−sectional national survey (KNHANES 2007–2013, 2015)	Adolescents (n = 2,324, aged 12–18) with both parents.	Observational (exposure: parental suicidal ideation)	Adolescents with vs. without parental suicidal ideation.	Primary Outcome: Suicidal ideation in adolescents. Main model: AOR = 2.01, 95% CI [1.32–3.05], p = .001; Depressive symptoms: AOR = 5.43, 95% CI [3.66–8.04], p < .001; High stress level: AOR = 15.51, 95% CI [6.14–39.19], p < .001; Subgroup without depressive symptoms: AOR = 1.92, 95% CI [1.20–3.08], p = .006; Subgroup with depressive symptoms: AOR = 4.05, 95% CI [0.83–19.75], p = .084; By sex: Boys: AOR = 0.91, 95% CI [0.41–2.01], p = .811; Girls:	Chi−square tests; multivariable logistic regression adjusted for child sex, grade, depressive symptoms, stress level, health status, physical activity, parental income/education/occupation/alcohol/smoking.	Parental suicidal ideation significantly predicts adolescent suicidal ideation, especially among girls, those with high stress, and where fathers had suicidal ideation. Prevention efforts should target these high−risk family configurations.

No.	Citation	Study Type	Participant Details	Exposure / Intervention Assessed	Group Allocation	Outcomes Evaluated	Analysis Approach	Major Findings
						AOR = 3.20, 95% CI [1.83–5.57], p < .001;<br>By grade: Elementary: AOR = 1.43, 95% CI [0.14–14.36], p = .552; Middle: AOR = 1.64, 95% CI [0.90–2.98], p = .107; High: AOR = 3.26, 95% CI [1.49–7.12], p = .003;<br>By parental source: Fathers only: AOR = 3.11, 95% CI [1.08–8.97]; Both parents: AOR = 1.27, 95% CI [0.30–5.53]; Mothers only: reference.		
**5**	Chan et al., 2018, New Zealand	Cross−sectional national survey (Youth'12)	Secondary school students (n = 8,500, ages 13–18) from 91 randomly selected schools; response rate 68%.	None – naturalistic exposure to suicidal behavior (attempt/death) in family/friends/school.	Exposed vs. not exposed to suicide attempt or death of a close contact.	Primary Outcome – Suicide Attempt (past 12 months):<br>Family Attempt >1 yr: AOR = 2.06 (95% CI [1.46–2.90]);<br>Family Attempt <1 yr: AOR = 4.98 (95% CI [3.81–6.52]);<br>Family Death >1 yr: AOR = 1.48 (95% CI [1.05–2.09]);<br>Family Death <1 yr: AOR = 3.12 (95% CI [2.03–4.81]);<br>Friend Attempt >1 yr: AOR = 1.94 (95% CI [1.38–2.71]);<br>Friend Attempt <1 yr: AOR = 3.89 (95% CI [3.01–5.04]).<br>Friend Death >1 yr: AOR = 1.59 (95% CI [1.06–2.37]);<br>Friend Death <1 yr: AOR = 2.81	Multivariable logistic regression with complex survey weights; Generalized Linear Mixed Models (GLMM); Chi−square tests; Adjusted for sex, age, ethnicity, deprivation, and mood.	Recent exposure to suicide attempts or deaths in family/friends was significantly associated with increased suicide attempts and NSSI. School−level exposure was not significantly associated. Results underscore importance of proximity and timing in suicide contagion effects.

No.	Citation	Study Type	Participant Details	Exposure / Intervention Assessed	Group Allocation	Outcomes Evaluated	Analysis Approach	Major Findings
						(95% CI [2.01–3.95]); School Suicide Death: AOR = 0.91 (95% CI [0.67–1.24]). Secondary Outcome – Repeated NSSI (≥3 times): Family Attempt <1 yr: AOR = 4.68 (95% CI [3.55–6.17]); Friend Attempt <1 yr: AOR = 3.41 (95% CI [2.76–4.22]); Family Death <1 yr: AOR = 2.41 (95% CI [1.66–3.49]); Friend Death <1 yr: AOR = 2.18 (95% CI [1.57–3.03]); School Suicide: AOR = 0.97 (95% CI [0.73–1.28]).		
**6**	Christiansen et al., 2024, Denmark	Nationwide cohort	384,569 adolescents (birth cohort 1983–1989, followed from age 10 to 35)	Parental suicide attempt (without own mental illness)	Adolescents with vs. without parental suicide attempt	Suicide attempt in offspring	Cox regression (time−dependen t covariates), multistate modeling	**Adjusted OR = 1.68 (95% CI: 1.51–1.87)** for suicide attempt in offspring exposed to parental suicide attempt only (no own mental illness), compared to unexposed
**7**	Cluver et al., 2015, South Africa	Prospective longitudinal cohort study	Adolescents aged 10–18 years (n = 3,515, 56% female), from urban and rural communities in two South African provinces.	None – observational. Exposure to cumulative ACEs (e.g., parental AIDS−related death/illness, homicide,	Stratified by ACE score: 0, 1–2, 3–4, ≥5 ACEs.	Suicide Attempt: OR = 2.46, 95% CI [1.00–6.05]; Suicide Planning: OR = 4.40, 95% CI [2.08–9.29]; Suicide Ideation: OR = 2.99, 95% CI [1.68–5.53]; Mental health significantly mediated these associations; substance misuse did not.	MINI−KID suicidality scale. Logistic regression controlling for sociodemograph ics and baseline suicidality.	Cumulative ACEs strongly predicted suicidality after 1 year. Mental health mediated effects. No mediation by substance use. Authors recommend scalable mental health services

No.	Citation	Study Type	Participant Details	Exposure / Intervention Assessed	Group Allocation	Outcomes Evaluated	Analysis Approach	Major Findings
			Baseline 2009–2010, follow−up after 1 year. Retention: 96.8%.	physical/emotional/sexual abuse, domestic violence, community violence, food insecurity).			Mediation tested with PROCESS macro (1,000 bootstraps). Psychological measures: CDI, RCMAS, PTSD Checklist, substance use index.	and adversity prevention strategies in LMICs.
**8**	Easey et al., 2019, UK	Population−based Longitudinal Study (ALSPAC Cohort)	Adolescents aged 15–16 years (n = 2,571); after exclusion of only−children and cases with infant death; complete data available in n = 2,206.	Exposure: birth order (firstborn, second−born, third+ born); mediators: number of maternal depressive episodes (EPDS across 18 and 32 weeks gestation, 8 weeks, 8, 21, 33, 61 months postpartum), father absence (categorized: present, absent before age 5, absent after age	Birth order groups compared on suicide attempts and psychiatric disorders; mediation effects tested separately and jointly for maternal depression and father absence.	Suicide attempts: firstborn 5% (44/924), second−born 7% (59/838), third+ born 7% (25/316). Psychiatric disorders: firstborn 4% (42/926), second−born 6% (51/846), third+ born 7% (23/318). Adjusted odds ratios (OR) and 95% confidence intervals (CI): linear trend for suicide attempts OR = 1.42 (95% CI = 1.10–1.84, p = 0.006); second−born OR = 1.56 (95% CI = 1.05–2.31), third+ born OR = 1.97 (95% CI = 1.17–3.34). Psychiatric disorders: linear trend OR = 1.29 (95% CI = 0.99–1.69, p = 0.056); second−born OR = 1.21 (95%	Multivariable logistic regression using Stata version 14.1; mediation analysis using Mplus version 7; multiple imputation by chained equations (MICE) with 100 imputed datasets; adjustments for maternal age, social class, income, gestational age,	Later−born adolescents showed significantly higher odds of suicide attempts and psychiatric disorders compared to firstborns. Maternal depression and father absence partially mediated the associations (8%–12% explained). Findings suggest importance of addressing family dynamics, maternal mental health, and paternal presence when designing preventive interventions for adolescent suicidality. Future research

No.	Citation	Study Type	Participant Details	Exposure / Intervention Assessed	Group Allocation	Outcomes Evaluated	Analysis Approach	Major Findings
				5). No clinical intervention tested.		CI = 0.81–1.80), third+ born OR = 1.72 (95% CI = 1.01–2.94). Mediation analysis (fully adjusted): suicide attempts, total effect β = 0.120 (SE = 0.042, p = 0.005), maternal depressive episodes indirect effect β = 0.006 (SE = 0.003, percentage mediated = 6.5%), father absence indirect effect β = 0.006 (SE = 0.003, percentage mediated = 6.25%), combined indirect effect β = 0.010 (SE = 0.016, percentage mediated = 8%). Psychiatric disorders, total effect β = 0.090 (SE = 0.044, p = 0.039), maternal depressive episodes indirect effect β = 0.008 (SE = 0.004, percentage mediated = 9%), father absence indirect effect β = 0.005 (SE = 0.003, percentage mediated = 6%), combined indirect effect β = 0.011 (SE = 0.005, percentage mediated = 12%).	alcohol and tobacco use during pregnancy; Wald tests for interaction; path analyses using probit regression coefficients for categorical outcomes.	recommended to explore alternative pathways such as sibling bullying and socioeconomic stressors.
**9**	Giletta et al., 2015, China	Multiwave Prospective Longitudinal Study	Tenth–grade adolescents (n = 565, Mage = 16.03, SD = 0.52); 48.3%	Not an intervention study; exposure variables: peer	Trajectory groups (low, moderate, high) for suicide ideation and	Suicide ideation measured by SIQ (mean total scores at baseline: M = 15.22, SD = 12.56); NSSI measured by	Latent class growth analysis using GBTM; joint trajectory	Adolescents experiencing high peer victimization and depressive symptoms were more likely to

No.	Citation	Study Type	Participant Details	Exposure / Intervention Assessed	Group Allocation	Outcomes Evaluated	Analysis Approach	Major Findings
			male; urban Changsha and rural Liuyang, Hunan Province, China; 82.8% living with both parents.	victimization, depressive symptoms, friendship characteristics.	NSSI; high–high group vs. others; peer victimization predictors.	DSHI (mean at baseline: M = 1.46, SD = 2.91); suicide attempts (binary) reported at each wave (e.g., 3 months: 6.1%; 6 months: 3.0%; 12 months: 1.5%; 24 months: 0.5%). High–high trajectory group (~9.2% of sample) showed significantly higher risk of future suicide attempts: OR = 5.09, 95% CI = 1.19–21.82, p = .029. Peer victimization predicted high–high membership: OR = 1.25, 95% CI = 1.02–1.52, p = .03. Depressive symptoms predicted SI/NSSI trajectories: OR = 1.04, 95% CI = 1.01–1.07, p = .008. No significant sex differences (p = .85).	modeling; multinomial logistic regression; GEE models accounting for repeated measures; Bayesian Information Criterion (BIC), Lo−Mendell−Rubin Ratio Likelihood Test (LMR−LRT) used for model selection; missing data handled via multiple imputation; software: Mplus 6.0, SPSS 21.	follow high−risk joint trajectories of suicide ideation and NSSI. Membership in the high–high group significantly increased future suicide attempt risk (fivefold). Findings highlight peer victimization as a critical predictor and suggest need for preventive school−based interventions focusing on bullying and peer relationships.
**10**	Goldston et al., 2016, USA	Prospective Naturalistic Longitudinal Study with Bayesian Growth	Adolescents (n = 180) (ages 12–18; 51% female; 80% European American), consecutively hospitalized in	No intervention; longitudinal tracking of psychiatric, psychosocial, functional	Four latent STB trajectory classes: Increasing Risk (11%, n = 20), Highest Overall Risk (12%, n = 22), Decreasing Risk	STBs over time (ordinal 1–5 scale per assessment). Highest Overall Risk class: greatest post−hospitalization suicide attempt rates, MDD/GAD duration, hopelessness (BHS M = 10.42, SD = 6.29, p < .01),	Bayesian finite mixture models using Monte Carlo Markov Chain (MCMC) simulations; quadratic	STB developmental trajectories among psychiatrically hospitalized youth show strong heterogeneity: Highest Overall Risk group marked by

No.	Citation	Study Type	Participant Details	Exposure / Intervention Assessed	Group Allocation	Outcomes Evaluated	Analysis Approach	Major Findings
		Mixture Modeling	psychiatric care, followed up to 19.3 years (avg. 13.6 years, SD = 4.5), total 2,273 assessments (range 2–26 per participant; avg. 12.6, SD = 5.1). Pre–hospitalization: 42% had attempted suicide, 33% ideation; 38% MDD, 17% GAD, 15% ADHD, 10% CD, 10% ODD, 12% panic disorder, 13% substance abuse, 13% parental suicide attempt.	predictors and STB trajectories.	(33%, n = 60), Low Risk (44%, n = 78).	trait anxiety (STAI M = 47.16, SD = 11.74, $p < .01$), sexual abuse history (47%). Increasing Risk class: higher adult aggression (b = 6.23, SE = 2.88, $p < .05$), impulsivity (BIS–11 b = 10.02, SE = 3.29, $p < .01$), role performance impairment (CAFAS b = 1.08, SE = 0.52, $p < .05$), behavioral appropriateness impairment (CAFAS b = 0.94, SE = 0.43, $p < .05$). Protective factors: coping beliefs (Reasons for Living b = –0.79, SE = 0.25, $p < .01$), social support (Social Support Questionnaire M = 4.25, SD = 0.78). No significant class differences in school dropout ($p = .32$), incarceration ($p = .45$). Functional outcomes: social adjustment impairment (SAS–SR Highest Overall Risk b = 11.07, SE = 3.62, $p < .01$), role impairment, behavioral appropriateness.	growth mixture modeling; logistic and linear regressions for covariate–outcome associations; repeated measures analyzed semi–parametrically; interrater reliability κ = 0.92 for STB coding; psychiatric diagnoses (DSM–IV) assessed via ISCA and FISA interviews; psychosocial predictors measured repeatedly (BHS, STAI, BIS–11, Social Support Questionnaire, Reasons for	enduring hopelessness, anxiety, sexual abuse history; Increasing Risk group associated with adult aggression, impulsivity, functional impairments. Protective factors (coping beliefs, social support) mitigate risk. Findings emphasize need for long–term, tailored interventions addressing psychiatric and psychosocial predictors. Future research should refine predictive models and inform individualized treatment pathways.

No.	Citation	Study Type	Participant Details	Exposure / Intervention Assessed	Group Allocation	Outcomes Evaluated	Analysis Approach	Major Findings
							Living Inventory).	
**11**	Halonen et al., 2019, Finland	Nationwide Population−Based Prospective Cohort Study with Counterfactual Mediation Analysis	Individuals (n = 52,182) (50.9% male, n = 26,582; 49.1% female, n = 25,600), born in 1987 in Finland, followed through nationwide registers; exclusions: early death, intellectual disability, permanent disability allowance, emigration. Parental mental disorders: 12% (ICD−9 291–319, ICD−10 F10–F99); adolescent mental disorders: 8% (ICD−10 F00–F99); social disadvantage: 27% (low education, social assistance,	Observational study; no intervention; mediation analysis of how adolescent mental disorders and social disadvantage transmit parental mental disorder risk to offspring psychiatric work disability.	Exposed (parental mental disorder) vs. unexposed groups; mediation via adolescent−level factors.	Total effect: OR = 1.85 (95% CI = 1.46–2.34, $p < .001$); mediation via adolescent mental disorders: OR = 1.19 (95% CI = 1.11–1.28, $p < .001$), 35% mediation; mediation via adolescent social disadvantage: OR = 1.15 (95% CI = 1.06–1.24, $p < .001$), 28% mediation. Path coefficients (probit models): parental mental disorders → adolescent mental disorders $\beta = 0.366$ (95% CI = 0.323–0.408, $p < .001$); parental mental disorders → social disadvantage $\beta = 0.456$ (95% CI = 0.423–0.490, $p < .001$); adolescent mental disorders → work disability $\beta = 0.421$ (95% CI = 0.379–0.463, $p < .001$); social disadvantage → work disability $\beta = 0.080$ (95% CI = 0.035–0.124, $p < .001$). Sex−stratified mediation: women – adolescent mental disorders 55%, social disadvantage 65%; men –	Counterfactual mediation analysis using SAS macro by Valeri & VanderWeele; probit regression models estimated in R (lavaan package); logistic regression for odds ratios; subgroup analyses by sex and geographical region; data linkage via Finnish national registers; no missing data after exclusions. Sensitivity analyses included	The intergenerational transmission of psychiatric work disability is partly mediated by adolescent mental disorders and social disadvantage, highlighting adolescence as a critical window for intervention. Sex differences suggest stronger mediation among women, particularly via social disadvantage, whereas effects among men are more modest. Geographical variation underscores the influence of regional socioeconomic contexts. Preventive strategies should target adolescent mental health, educational attainment, and family support systems to mitigate future psychiatric disability. The study provides robust

No.	Citation	Study Type	Participant Details	Exposure / Intervention Assessed	Group Allocation	Outcomes Evaluated	Analysis Approach	Major Findings
			parental unemployment, low GPA).			adolescent mental disorders 22%, social disadvantage 11%. Regional stratification: Southern/Western Finland OR = 2.14 (95% CI = 1.61–2.84); Northern/Eastern Finland OR = 1.36 (95% CI = 1.00–1.85). Sensitivity analyses (education cutoff at age 21) yielded comparable estimates.	alternative definitions of educational attainment. Robust standard errors reported; full−model path diagrams presented.	evidence from a nationwide cohort, though findings should be cautiously generalized outside Nordic welfare contexts.
**12**	Hammerton et al., 2015, UK	Population−Based Longitudinal Cohort Study with Structural Equation Modeling (ALSPAC)	Mother−offspring pairs (n = 10,559) (40% minimal maternal depression, 55% moderate, 5% chronic−severe); 4,588 with suicide−related data; 2,445 with complete psychopathology data; offspring assessed at age 15 for psychopathology (MDD, GAD, DBD, ADHD, alcohol abuse), at age 16 for suicidal	Observational; no intervention; focus on individual mediators (the child); analysis of how offspring psychopathology mediates the relationship between maternal depression and offspring suicidal outcomes.	Chronic maternal depression vs. minimal/moderate depression; mediation via adolescent psychopathology symptoms.	Suicidal ideation (past year, age 16): prevalence 15.3% (95% CI: 14–17%); suicide attempt (lifetime, age 16): prevalence 7.7% (95% CI: 7–9%). Multiple imputation indirect effects for ideation: MDD B = 0.10 (95% CI: 0.06–0.15), GAD B = 0.06 (95% CI: 0.03–0.09), DBD B = 0.11 (95% CI: 0.06–0.16), ADHD B = –0.02 (95% CI: –0.06–0.02), alcohol abuse B = 0.02 (95% CI: 0.00–0.04); direct effect B = 0.36 (95% CI: 0.17–0.55, $p < .001$). Suicide attempt indirect effects: MDD B = 0.11 (95% CI: 0.06–0.16), GAD B = 0.07 (95% CI: 0.03–0.11), DBD B = 0.11 (95% CI: 0.06–0.16), ADHD B = 0.06 (95% CI: 0.01–	Structural equation modeling (SEM) using Mplus; probit regression; multiple mediation models; bias−corrected bootstrapping (500 replications); multiple imputation by chained equations; Wald χ^2 post−hoc tests; complete case and	Maternal depression influences adolescent suicidal ideation and attempt both directly and through indirect pathways mediated by adolescent psychopathology. MDD, GAD, and DBD mediate risk for both ideation and attempt, while ADHD uniquely mediates the transition from ideation to attempt. Alcohol abuse plays a minimal mediating role. Clinical interventions should account for multiple psychopathology pathways, focusing on

No.	Citation	Study Type	Participant Details	Exposure / Intervention Assessed	Group Allocation	Outcomes Evaluated	Analysis Approach	Major Findings
			ideation and attempts.			0.12), alcohol abuse B = 0.02 (95% CI: –0.00–0.03); direct effect B = 0.31 (95% CI: 0.10–0.52, p = .003). Wald χ^2 tests showed indirect effects via MDD, GAD, DBD stronger than ADHD, alcohol abuse ($p \le .037$). Sensitivity analyses (complete case n = 2,445) produced slightly attenuated indirect effects but same directional patterns. Subgroup analysis (ideators only): ADHD specifically predicted transition from ideation to attempt (B = 0.17, 95% CI: 0.05–0.42).	imputed sensitivity analyses; adjustment for confounders (housing tenure, marital status, maternal education, psychiatric history, maternal smoking, family depression history); subgroup analysis on ideators.	both internalizing and externalizing symptoms. Results underline the importance of monitoring adolescents exposed to chronic maternal depression for early signs of diverse psychopathologies, not just depression. Future research should explore additional non−psychopathological mediators.
**13**	Hammerton et al., 2016, UK	Population−Based Longitudinal Cohort Study with Structural Equation Modeling (ALSPAC)	Mother−offspring pairs (n = 10,559) (40% minimal depression, 55% moderate, 5% chronic−severe); suicidal ideation at age 16 assessed in 4,588 adolescents (15%, 11% males, 20% females);	Observational study; no intervention; expanded focus on individual + relational (family) mediators; mediation analysis of maternal depression pathways to	Minimal vs. moderate vs. chronic−severe maternal depression; mediation via offspring psychiatric disorder, parent−child relationship, maternal suicide attempt.	Chronic−severe vs. minimal: offspring suicidal ideation OR = 3.04 (95% CI: 2.19–4.21); offspring psychiatric disorder OR = 5.51 (95% CI: 3.92–7.74); maternal suicide attempt OR = 36.26 (95% CI: 20.12–65.37); parent−child relationship β = 0.26 (95% CI: 0.15–0.37), $p < .001$. Total effect B = 0.71 (95% CI: 0.49–0.93); 46% mediated via maternal suicide attempt B	Structural equation modeling (SEM) using Mplus v7; weighted least squares estimator (WLSMV); bias−corrected bootstrapping (500 replications);	Maternal depression increases adolescent suicidal ideation risk through multiple indirect pathways, particularly maternal suicide attempt and offspring psychiatric disorder; parent−child relationship adds an independent, albeit smaller, mediating effect. Chronic−severe maternal

No.	Citation	Study Type	Participant Details	Exposure / Intervention Assessed	Group Allocation	Outcomes Evaluated	Analysis Approach	Major Findings
			maternal suicide attempt (2%) across 10 waves; psychiatric disorders at age 15 (9%) via DAWBA. Parent–child relationship score (age 9). mean: minimal 2.94, moderate 3.46, chronic–severe 4.15.	offspring suicidal ideation.		= 0.26 (95% CI: 0.07–0.45), 45% via offspring disorder B = 0.27 (95% CI: 0.12–0.42), 6% via parent–child relationship B = 0.04 (95% CI: 0.02–0.07), 1% via both B = 0.01 (95% CI: 0.004–0.02). Gender stratification: indirect effect via psychiatric disorder stronger in females ($p = .015$). Sensitivity analyses across imputation models (full n = 10,559; imputed subsamples n = 8,475 and n = 4,588; complete cases n = 2,842) showed robust findings; slightly attenuated indirect effects in complete case analyses. Model fit indices: RMSEA = 0.003, CFI = 1.000. Post–hoc Wald χ^2 tests showed stronger indirect effects for chronic–severe compared to moderate depression ($p < .037$).	probit regression for categorical outcomes; full multiple imputation by chained equations; adjustment for confounders (gender, housing, marital status, education, smoking, psychiatric history, family depression history); sensitivity analyses across imputation strategies; subgroup analyses by gender; post–hoc Wald χ^2 tests for equality constraints;	depression produces stronger mediation effects than moderate depression. Gender analyses highlight stronger indirect effects via offspring psychiatric disorder in females. Clinical interventions should target maternal suicidal behavior, adolescent psychiatric screening, and family relationship improvement to effectively reduce intergenerational suicide risk. Results emphasize the value of addressing both clinical and relational mechanisms in prevention strategies.

No.	Citation	Study Type	Participant Details	Exposure / Intervention Assessed	Group Allocation	Outcomes Evaluated	Analysis Approach	Major Findings
							model fit indices reported (RMSEA, CFI).	
**14**	Han et al., 2023, Korea	Case−Control Study with PSM and Complex Sample Logistic Regression	National Korean adolescents aged 12–18, weighted total ~3.91 million; analytic sample n = 6,512 (SI group n = 428, weighted ~261,645; MC group n = 421, weighted ~243,622); within SI: HS n = 69, weighted ~36,934; N−HS n = 331, weighted ~224,711. Maternal diagnosed depression 2.3%, maternal depressive mood 3.4%, maternal suicidal ideation 1.5%, maternal alcohol use 7.5%, maternal	None (observational study).	SI vs MC; HS vs N−HS within SI group; interaction models.	SI vs MC (Model 3): maternal diagnosed depression OR 2.109 (1.023–4.350, p = .043), maternal depressive mood OR 2.155 (1.224–3.793, p = .008), maternal suicidal ideation OR 2.532 (1.322–4.851, p = .005), adolescent depressive mood OR 6.759 (4.315–10.586, p < .001), adolescent smoking OR 3.501 (1.859–6.593, p < .001), adolescent help−seeking OR 4.847 (2.547–9.222, p < .001). HS vs N−HS (Model 3): maternal depressive mood OR 4.486 (1.312–15.34, p = .017), maternal suicidal ideation OR 0.150 (0.031–0.721, p = .018), maternal help−seeking OR 17.495 (1.812–168.877, p = .014), adolescent depressive mood OR 9.417 (2.775–31.944, p < .001), adolescent smoking OR 3.772 (1.125–12.648, p = .031). Interaction: maternal depressive mood stronger for HS subgroup OR 2.911 (1.473–	Propensity Score Matching using R 4.2.3; complex sample logistic regression using IBM SPSS 27.0; three−step progressive modeling: Model 1 (unadjusted), Model 2 (adjusted for demographics), Model 3 (fully adjusted with all covariates); interaction effect analysis using R effects package; p−values and ORs with 95% CIs reported; weighting for national representativene	Acute maternal depressive mood and suicidal ideation significantly increase adolescent suicidal ideation risk; however, maternal suicidal ideation reduces adolescent help−seeking, while maternal depressive mood increases it. Chronic maternal depression shows weaker association. Adolescent depressive mood and smoking strongly predict suicidal ideation and help−seeking patterns. Study highlights nuanced, differential effects of maternal psychopathology on adolescent suicidality and the need for family−centered suicide prevention strategies in Korean adolescents.

No.	Citation	Study Type	Participant Details	Exposure / Intervention Assessed	Group Allocation	Outcomes Evaluated	Analysis Approach	Major Findings
			help−seeking 0.9%. Adolescent depressive mood 28.7%, adolescent smoking 15.3%, adolescent help−seeking 18.4%.			5.755), maternal suicidal ideation reduces help−seeking OR 0.150 (0.031–0.721). Full descriptive breakdown of each factor across groups provided in weighted frequencies.	ss; significance set at p < .05. Ethical approvals secured.	
**15**	Jeong et al., 2020, Korea	Cross−sectional, retrospective secondary data analysis using 11 years of KYRBS.	Korean adolescents (n = 788,411) (aged 13–18) from middle and high schools, 2007–2017; representative national sample.	Not an intervention; observational epidemiological analysis of predictors of suicidal ideation and suicide attempts. Category: Epidemiological Observational Studies.	Adolescents with vs. without suicidal ideation and suicide attempts; comparisons across demographic, behavioral, psychological, and socioeconomic subgroups.	Primary outcomes: Suicidal ideation and suicide attempts (past 12 months). Results (Model 3, Adjusted Odds Ratios [aOR], 95% Confidence Intervals [CI]):<br>Suicidal ideation — Female OR 1.38 (1.31–1.41); 1st year middle school OR 1.82 (1.65–1.93); Low SES OR 1.18 (1.02–1.21); Living without parents OR 1.53 (1.31–1.67); Breakfast <2x/week OR 1.04 (1.01–1.18); Smoking OR 1.28 (1.21–1.31); Alcohol use OR 1.17 (1.11–1.23); Problem drinking OR 1.24 (1.18–1.29); Frequent stress OR 5.51 (4.15–5.75); Unhappy OR 5.49 (5.14–5.82); Depression OR 5.78 (5.64–5.88).<br>Suicide attempts — Female OR	Complex sample−weighted multivariate logistic regression (SPSS v21); Model 1 adjusted for sex and grade; Model 2 added socioeconomic and behavioral factors (residential type, SES, academic achievement, breakfast skipping, smoking, alcohol use, problem drinking);	Depression was the strongest predictor of both suicidal ideation (OR 5.78) and suicide attempts (OR 5.56), followed by frequent stress and perceived unhappiness. Significant demographic and behavioral predictors included female sex, younger age (especially first−year middle school), living without parents, low SES, breakfast skipping, smoking, alcohol use, and problem drinking. Despite observed decreasing trends in prevalence over the 11−year period, the high rates of suicidal

No.	Citation	Study Type	Participant Details	Exposure / Intervention Assessed	Group Allocation	Outcomes Evaluated	Analysis Approach	Major Findings
						1.64 (1.54–1.71); 1st year middle school OR 2.91 (2.73–3.12); Low SES OR 1.22 (1.09–1.31); Living without parents OR 1.95 (1.46–2.74); Breakfast <2x/week OR 1.14 (1.11–1.19); Smoking OR 1.27 (1.21–1.32); Alcohol use OR 1.21 (1.17–1.27); Problem drinking OR 1.25 (1.20–1.31); Frequent stress OR 5.32 (4.51–5.92); Unhappy OR 5.12 (4.48–5.67); Depression OR 5.56 (5.22–5.83).	Model 3 further adjusted for psychological variables (subjective health, happiness, stress, depressive symptoms). Statistical significance set at $p < 0.05$. Stratified analyses reported.	behaviors among Korean adolescents highlight the urgent need for early mental health interventions and targeted suicide prevention efforts, particularly addressing depressive symptoms and stress.
**16**	Kawabe et al., 2016, Japan	Population−based cross−sectional study using GHQ−30 and POMS questionnaires..	Junior high school students (n = 185) (aged 12–15) and their caregivers (154 mothers, 25 fathers, 6 others) from Kumakogen Town, Ehime Prefecture, Japan; 90 males, 95 females; survey conducted in September 2012.	Not an intervention; observational assessment of adolescent and caregiver mental health to identify predictors of suicidal ideation. Category: Epidemiological Observational Studies.	Adolescents with vs. without suicidal ideation; comparisons across GHQ−30 and POMS subscales for both adolescents and caregivers; multivariate regression adjusting for demographic, psychological,	Primary outcome: adolescent suicidal ideation (GHQ item 30 score >2).<br>Results (Adjusted Odds Ratios [OR], 95% Confidence Intervals [CI]):<br>Student factors — GHQ somatic symptoms OR 8.58 (1.39–53.03); POMS confusion–bewilderment OR 15.49 (3.79–63.26).<br>Caregiver factors — caregiver mother vs. other OR 7.29 (1.71–31.11); caregiver GHQ	Multivariate logistic regression (SPSS v22, stepwise backward selection) adjusting for demographic (sex, grade), psychological (GHQ and POMS subscales) variables; GHQ	The study demonstrated that caregiver mental health, particularly caregiver suicidal depression (OR 72.13), was the most important factor associated with adolescent suicidal ideation, even after adjusting for adolescent mental health and behavioral factors. Findings highlight the need for suicide

No.	Citation	Study Type	Participant Details	Exposure / Intervention Assessed	Group Allocation	Outcomes Evaluated	Analysis Approach	Major Findings
					and behavioral variables.	suicidal depression OR 72.13 (12.60–412.96), the strongest predictor overall. Other significant univariate predictors included caregiver POMS fatigue–inertia (crude OR 3.02, 95% CI 1.04–8.78) and caregiver POMS confusion–bewilderment (crude OR 3.45, 95% CI 1.24–9.65).	suicidal depression excluded from student variables to avoid collinearity; cut−off thresholds applied (GHQ: ≥3 or ≥4 per subscale, POMS: T−score ≥60). Significance set at $p < 0.05$. Analyses included descriptive, Mann–Whitney U, Wilcoxon signed−rank tests, and confidence intervals.	prevention strategies that target both adolescents and their caregivers, emphasizing family−based interventions. The study also suggests that routine assessment of caregiver mental health can provide early warning indicators of risk in adolescents. Despite limitations (cross−sectional design, small sample, limited generalizability), the findings provide novel evidence supporting the inclusion of caregiver assessments in adolescent suicide prevention programs.
**17**	Kendler et al., 2020, Sweden	Population−wide observational cohort study using Swedish	Offspring born 1960–1990 and their biological or adoptive parents in Sweden (n = 2,417,104).	Not an intervention; observational analysis of genetic and environmental	Offspring of parents with vs. without history of suicide attempt and/or suicide death;	Primary outcomes: suicide attempt, suicide death in offspring. Results (Tetrachoric Correlations [TC], 95% CI): Suicide attempt → suicide attempt: Mother−offspring	Tetrachoric correlations computed separately for mother−child and father−child	The study demonstrated that suicide attempt is transmitted across generations via both genetic and rearing mechanisms, with

No.	Citation	Study Type	Participant Details	Exposure / Intervention Assessed	Group Allocation	Outcomes Evaluated	Analysis Approach	Major Findings
		national registers.	Subsamples: intact families (n = 2,175,259), not−lived−with father families (n = 152,436), stepfather families (n = 73,785), adoptive families (n = 15,624).	transmission of suicide−related outcomes. Category: Epidemiological Genetic Observational Studies.	comparisons across genes+rearing, genes−only, rearing−only pathways; parent−child pairings stratified by parent/child sex and relationship type.	genes+rearing: TC=0.22 (0.21–0.23); Father−offspring genes+rearing: TC=0.24 (0.24–0.25); Mother−offspring genes−only: TC=0.14 (0.09–0.19); Father−offspring genes−only: TC=0.13 (0.11–0.15). Mother−offspring rearing−only: TC=0.10 (0.03–0.17); Father−offspring rearing−only: TC=0.14 (0.11–0.17). Odds Ratios for suicide attempt (parallel to TC): Genes+rearing: OR=3.28 (3.21–3.35); Genes−only: OR=1.76 (1.66–1.86); Rearing−only: OR=1.89 (1.67–2.13). Sex−specific transmission (TC): − Genes+rearing mother−daughter: TC=0.23 (0.22–0.24); mother−son: TC=0.26 (0.25–0.27); father−daughter: TC=0.21 (0.20–0.22); father−son: TC=0.28 (0.27–0.29). Genes−only mother−daughter: TC=0.12 (0.04–0.19); mother−son: TC=0.16 (0.09–	pairings; logistic regression models adjusted for parental psychiatric disorders (major depression, anxiety, substance use disorders, bipolar, psychosis); linear probability models estimated attenuation of genetic transmission by psychiatric disorders (40% reduction in genes−only pathway; no reduction in rearing−only); meta−analysis using Olkin−Pratt method;	transmission stronger to sons than daughters in genes+rearing and genes−only pathways. Parental psychiatric illness accounted for ~40% of genetic transmission but did not attenuate rearing effects. Suicide death showed modest genetic transmission (TC=0.07) and no rearing effect. Suicide attempt and suicide death were highly genetically correlated (r=0.84) but represented distinct liabilities, rejecting a single continuum model. Findings highlight the importance of considering both genetic and environmental pathways in suicide prevention across generations.

No.	Citation	Study Type	Participant Details	Exposure / Intervention Assessed	Group Allocation	Outcomes Evaluated	Analysis Approach	Major Findings
						0.23); father−daughter: TC=0.14 (0.11–0.16); father−son: TC=0.20 (0.18–0.22). Rearing−only mother−daughter: TC=0.04 (–0.08–0.15); mother−son: TC=0.15 (0.05–0.25); father−daughter: TC=0.12 (0.09–0.15); father−son: TC=0.16 (0.13–0.20). Suicide death → suicide death (TC): Genes+rearing: TC=0.16 (0.15–0.18); Genes−only: TC=0.07 (0.02–0.12); Rearing−only: TC=–0.05 (–0.17–0.07). Cross−transmission attempt → death: genes+rearing: TC=0.11 (0.10–0.12); genes−only: TC=0.08 (0.04–0.12); rearing−only: TC=0.06 (0.02–0.09).	heterogeneity tests across family types; stratified analyses by parent and offspring sex. Statistical software: SAS 9.4, R 3.6.1; significance p<0.05.	
**18**	Lee et al., 2021, Korea	Cross−sectional, population−based study using Korea National Health and	Adolescent girls (n = 890), aged 12–18 years and parents (n = 1,500), 645 fathers, and 855 mothers, nationally	Not an intervention; observational analysis of parental risk factors associated with adolescent	Adolescent girls with vs. without suicide attempt in the past year; parental factors stratified by maternal and	Primary outcome: adolescent girls' suicide attempt in past year. Results (Hierarchical Logistic Regression, Adjusted Odds Ratios [OR], 95% Confidence Intervals [CI], p−values):	Hierarchical logistic regression performed using SPSS v27; Step 1 included adolescent	The study identified maternal suicidal ideation (OR=292.92), maternal suicide attempt (OR=12.38), and maternal suicidal plan (OR=6.39) as significant predictors of

No.	Citation	Study Type	Participant Details	Exposure / Intervention Assessed	Group Allocation	Outcomes Evaluated	Analysis Approach	Major Findings
		Nutrition Examination Survey (KNHANES, 2015–2018).	representative sample from South Korea.	girls’ suicide attempts. Category: Epidemiological Observational Studies.	paternal mental health and suicidal behaviors.	Step 1 (adolescent factors): Age: OR=0.89 (0.59–1.34), p=0.58; Household income: OR=0.87 (0.43–1.77), p=0.71; Sleep duration: OR=0.58 (0.27–1.24), p=0.16; Depressed mood: OR=4.87 (1.18–20.09), p=0.03; Suicidal ideation: OR=173.01 (10.68–2802.98), p<0.001; Suicidal plan: OR=8.37 (1.54–45.48), p=0.01.<br>Step 2 (adding maternal factors): Maternal stress: OR=1.12 (0.65–1.94), p=0.67; Maternal depressed mood: OR=1.48 (0.64–3.41), p=0.36; Maternal suicidal ideation: OR=292.92 (21.41–4062.14), p<0.001; Maternal suicidal plan: OR=6.39 (1.25–32.75), p=0.03; Maternal suicide attempt: OR=12.38 (1.56–98.26), p=0.02.<br>Paternal suicidal ideation, plan, and attempt were not statistically significant predictors in either step (p>0.05). Model fit: McFadden's pseudo−R^2=0.62; classification accuracy	factors (age, income, sleep duration, depressed mood, suicidal ideation, suicidal plan); Step 2 added maternal factors (stress, depressed mood, suicidal ideation, suicidal plan, suicide attempt). Variance inflation factors checked (VIF<5); Rao−Scott chi−square tests and weighted t−tests used for group comparisons; complex sample weights applied; significance threshold p<0.05; classification	adolescent girls' suicide attempts, independent of adolescents’ own suicidal ideation (OR=173.01). Paternal suicidal factors were not significant. These findings highlight the critical role of maternal mental health and suicidal behavior in adolescent suicide prevention. Interventions targeting maternal mental health may provide preventive effects. Limitations: cross−sectional design, reliance on self−reported measures, small number of adolescent suicide attempts (n=13), absence of longitudinal follow−up.

No.	Citation	Study Type	Participant Details	Exposure / Intervention Assessed	Group Allocation	Outcomes Evaluated	Analysis Approach	Major Findings
						increased by 8.9% with maternal factors.	accuracy and McFadden's pseudo−R^2 reported.	
**19**	Logeswaran et al., 2025, Denmark	Self−controlled case series (SCCS), population−based, national registry study	Offspring bereaved by parental suicide (n=188 with self−harm/suicide outcome), and by other causes (n=734 with outcome), Denmark, 1980−2016. Median age at bereavement: 14 (suicide), 18 (other causes). Both sexes included, all ages, national cohort.	Exposure: 2−year period centered on offspring's birthday marking the age at which their parent died by suicide (vs. 15−year flanking periods); Comparison group: offspring bereaved by parental non−suicide death	Bereaved offspring reaching the age at which their parent died (suicide vs. other causes)	Primary outcome: Any episode of medically severe self−harm or suicide (secondary−care hospital contact or death); measured during exposure and unexposed periods	Self−controlled case series using national registry linkage; Fixed−effects conditional Poisson regression; IRRs adjusted for age, marital status, income; Sensitivity and subgroup analyses	Reaching the age at which a parent died by suicide is associated with a significantly increased risk of self−harm or suicide among suicide−bereaved offspring (IRRadj=2.02, 95% CI 1.21–3.38); No increased risk for non−suicide bereaved; Elevated risk driven by the period prior to and around age correspondence; No significant effect observed after non−suicide parental death. Indicates need for targeted support approaching this age.
**20**	Maguire et al., 2022, Northern Ireland	Population−wide cohort study linking 2011 Census and mortality	Offspring residing with parents in Northern Ireland at Census 2011 (n = 618,970),	Not an intervention; observational analysis of association between parental	Offspring of parents with vs. without poor MH; stratified comparisons for no parent	Primary outcomes: offspring poor MH; offspring death by suicide. Results (Adjusted Odds Ratios [OR], 95% Confidence Intervals [CI], per model):	Logistic regression with cluster−robust standard errors (household clustering);	Parental poor MH significantly increased offspring poor MH (OR=2.79 for 1 parent, OR=5.25 for 2 parents). Offspring suicide risk

No.	Citation	Study Type	Participant Details	Exposure / Intervention Assessed	Group Allocation	Outcomes Evaluated	Analysis Approach	Major Findings
		data (2011–2016).	stratified by offspring age (≤23, ≥24 years); 11.6% living with ≥1 parent with poor MH.	poor MH and offspring MH and suicide. Category: Epidemiological Observational Studies.	affected, 1 parent affected, 2 parents affected; maternal vs. paternal; offspring age ≤23 vs. ≥24 years.	Offspring poor MH (any parent poor MH vs. none): Model 1: OR=2.99 (2.86–3.12); Model 2: OR=2.93 (2.80–3.06); Model 3: OR=2.89 (2.76–3.02); Model 4: OR=2.82 (2.69–2.95); Model 5: ORadj=2.79 (2.64–2.95).<br>Offspring poor MH (1 parent vs. none): Model 5: OR=2.79 (2.64–2.95).<br>Offspring poor MH (2 parents vs. none): Model 5: OR=5.25 (4.57–6.02).<br>Maternal poor MH (vs. none): Model 5: ORadj=2.84 (2.67–3.03).<br>Paternal poor MH (vs. none): Model 5: ORadj=2.63 (2.38–2.92).<br>Offspring suicide (any parent poor MH vs. none): Model 1: OR=1.94 (1.51–2.49); Model 2: OR=1.91 (1.49–2.46); Model 3: OR=1.88 (1.47–2.42); Model 4: OR=1.78 (1.36–2.32); Model 5: OR=1.76 (1.31–2.36).<br>Offspring suicide (1 parent vs. none): Model 5: ORadj=1.76 (1.31–2.36).	progressive adjustment across five models: Model 1 (offspring age, sex); Model 2 (adds religion, single−parent household); Model 3 (adds limiting long−term illness); Model 4 (adds housing tenure, house value, area deprivation quintile); Model 5 (adds offspring poor MH). Stratified analyses by offspring age (≤23, ≥24 years). Significance threshold p<0.05. Analysis performed in Stata 15.1. Unadjusted and	also elevated (OR=1.76 for 1 parent, OR=2.18 for 2 parents), particularly for offspring ≤23 years (OR=1.54 for 1 parent, OR=2.80 for 2 parents). Both maternal and paternal poor MH were associated with increased offspring suicide risk, with slightly higher risk for paternal poor MH. The association remained significant across multiple adjustment models, suggesting robust findings. Study supports integrated suicide prevention targeting parental and offspring MH, especially among younger offspring. Limitations include reliance on cross−sectional MH measurement, lack of temporality, and potential unmeasured confounding; strengths

No.	Citation	Study Type	Participant Details	Exposure / Intervention Assessed	Group Allocation	Outcomes Evaluated	Analysis Approach	Major Findings
						Offspring suicide (2 parents vs. none): Model 5: ORadj=2.18 (0.89–5.34).<br>Maternal poor MH → offspring suicide: Model 5: ORadj=1.69 (1.22–2.35).<br>Paternal poor MH → offspring suicide:<br>Model 5: OR=1.95 (1.13–3.35).<br>Offspring suicide age ≤23 years: 1 parent: ORadj=1.54 (1.06–2.25); 2 parents: OR=2.80 (1.02–7.65).<br>Offspring suicide age ≥24 years: 1 parent: OR=1.37 (0.83–2.26); 2 parents: OR=0.73 (0.10–5.24).	adjusted ORs reported with 95% CIs. Household clustering accounted in variance estimation.	include population−wide linkage and large sample.
**21**	Mok et al., 2016, Denmark	Population−based cohort study	All persons born in Denmark, 1967–1997 (n=1,743,525; 48.7% female); followed from age 15 until outcome, death, emigration, or Dec 31, 2012	Parental psychiatric diagnosis (substance use, schizophrenia, mood, anxiety, personality disorders, suicide attempt; national registry data)	Offspring with ≥1 parent with vs. without each psychiatric disorder or suicide attempt	**Suicide attempt in offspring:** Parental antisocial personality disorder: IRR = 3.96 (95% CI [3.72, 4.21]);<br>Parental cannabis misuse: IRR = 3.57 (95% CI [3.25, 3.92]);<br>Parental suicide attempt: IRR = 3.42 (95% CI [3.29, 3.55]);<br>Parental mood disorder: IRR = 2.04 (95% CI [1.97, 2.10]);<br>**If both parents affected:** IRR up to 4.75 (suicide attempt);<br>Risks consistent across sexes	Log−linear Poisson regression; adjusted for offspring age, sex, calendar year, SES, and interactions	Risks for suicide attempt and violent offending in offspring elevated across all parental psychiatric diagnoses; strongest for antisocial, cannabis misuse, suicide attempt. Early parental interventions may benefit offspring; findings suggest shared etiology for self−harm and violent behaviors.

No.	Citation	Study Type	Participant Details	Exposure / Intervention Assessed	Group Allocation	Outcomes Evaluated	Analysis Approach	Major Findings
						for suicide attempt, slightly higher for violent offending in females.		
**22**	O'Reilly et al., 2020, Sweden	Nationwide population cohort; offspring−of−siblings (genetically informed quasi−experimental design)	2,762,883 offspring born 1973–2001, Sweden. Age at risk: ≥12 years, both sexes; includes half− and full−siblings/cousins.	Parental suicidal behavior (suicide attempt or death before offspring age 18)	Exposed (offspring with parental suicidal behavior) vs. unexposed (without parental suicidal behavior); cousin−pair comparisons	Risk of suicidal behavior in offspring: General population: Maternal suicide attempt/death HR=2.74 (95% CI, 2.67–2.83); adjusted HR=1.75 (1.69–1.81). Cousin−pair (full−sibling) fixed effects: HR=1.62 (1.57–1.67); half−siblings: HR=1.57 (1.45–1.71). Quantitative behavior genetic modeling: 29.2% (95% CI, 5.29–53.11%) of association due to environmental factors, remainder genetic.	Quantitative behavior genetic models (ACE/structural equation modeling); Fixed−effects Cox proportional hazards models; Kaplan–Meier; multiple covariate adjustment	The intergenerational transmission of suicidal behavior is driven primarily by shared genetics, but ~15–30% is due to environmental factors specific to exposure. The risk persists after adjusting for psychiatric comorbidity and shared familial factors, especially with maternal exposure. Results robust across models.
**23**	Ortin−Peralta et al., 2024, USA	Longitudinal Cohort Study (ABCD Study)	Children (n = 9,194) (48.4% female; age 9–10 at baseline).	Parental suicide attempt or suicide death reported by caregiver via Family History Assessment Module Screener (FHAMS). Category: parental suicidal behavior.	Offspring with vs. without parental SA/SD	Lifetime SI at T1: OR=1.64 (1.23–2.19); Lifetime SA at T1: OR=4.14 (2.57–6.66); First−time SI at T2/T3: OR=1.45 (1.07–1.95); First−time SA at T2/T3: OR=1.47 (0.82–2.64). Mediation: parental SA/SD → first−time SI via negative urgency: OR=1.04 (1.01–1.08); via positive urgency: OR=1.03 (1.01–1.05); parental SA/SD → first−time SA via negative	Logistic regression adjusted for child's sex, race/ethnicity, internalizing problems, family structure, financial adversity; structural equation	Parental suicidal behavior significantly associated with offspring suicidal ideation and attempt. Negative and positive urgency mediated associations with small effect sizes. Intervention implications: focus on impulsivity−based pathways for suicide prevention in children of

No.	Citation	Study Type	Participant Details	Exposure / Intervention Assessed	Group Allocation	Outcomes Evaluated	Analysis Approach	Major Findings
						urgency: OR=1.14 (1.05–1.27); via positive urgency: OR=1.05 (1.01–1.11). Direct effects (c′ path): parental SA/SD → first−time SI: OR=1.32 (0.48–2.24); → first−time SA: OR=1.41 (0.44–2.31). Path a (parental SA/SD → UPPS−P facets): negative urgency: b=0.07 (0.03–0.14); positive urgency: b=0.10 (0.04–0.17); lack premeditation: b=0.04 (–0.01–0.10); lack perseverance: b=0.03 (–0.004–0.07); sensation seeking: b=0.04 (–0.02–0.11). Path b (UPPS facet → first−time SA): negative urgency: OR=6.30 (3.30–10.26); positive urgency: OR=1.59 (1.12–2.35); lack premeditation: OR=1.83 (1.24–2.59); lack perseverance: OR=2.16 (1.20–2.88); sensation seeking: OR=1.12 (0.76–1.85). Confirmatory Factor Analysis: RMSEA=0.030; CFI=0.946; SRMR=0.033. Factor loadings >0.40.	modeling (SEM) with bootstrapping (100 resamples); weights and cluster adjustments.	parents with suicidal behavior.

No.	Citation	Study Type	Participant Details	Exposure / Intervention Assessed	Group Allocation	Outcomes Evaluated	Analysis Approach	Major Findings
**24**	Ranning et al., 2022, Denmark	Prospective Cohort Study	Individuals (n = 4,419,651) aged ≥10 years (1980–2016).	Parental suicide attempt (ICD−8: E950–E959; ICD−10: X60–X84) registered in national health registers. Category: Parental Suicidal Behavior	Offspring with vs. without parental suicide attempt	Primary Outcome: First suicide attempt in offspring aged 10–19. Incidence Rate Ratios (IRR): Crude IRR (adolescent SA): 3.71 (95% CI: 3.53–3.89), p<0.001; Fully adjusted IRR: 2.12 (2.02–2.22), p<0.001. Stratified IRRs by age at parental SA exposure: 0–1 years: 4.71 (4.19–5.44), p<0.001; 2–5 years: 4.11 (3.81–4.44), p<0.001; 6–12 years: 3.62 (3.41–3.84), p<0.001; 13–17 years: 3.49 (3.29–3.70), p<0.001. Prenatal: 4.20 (3.95–4.46), p<0.001. Stratified IRRs by parent type: Maternal SA: 2.47 (2.36–2.58), p<0.001; Paternal SA: 2.12 (2.02–2.22), p<0.001; Step−mother SA: 2.20 (1.94–2.49), p<0.001; Step−father SA: 2.30 (2.17–2.45), p<0.001; Multiple parental SA: 4.10 (3.69–4.55), p<0.001. Interaction effects:	Poisson regression adjusted for child sex, age, parental psychiatric disorder, family structure, SES. Cox proportional hazards models for cumulative hazard. Clustered robust SEs. Sensitivity analyses by calendar period, parental comorbidities, family cohabitation.	Parental suicide attempt significantly increased the risk of adolescent suicide attempt, with stronger effects for maternal exposure and early−life exposure. Effect modification by child and parent sex. Supports early intervention targeting high−risk families.

No.	Citation	Study Type	Participant Details	Exposure / Intervention Assessed	Group Allocation	Outcomes Evaluated	Analysis Approach	Major Findings
						Parent sex × exposure: interaction p=0.002 (maternal stronger than paternal)<br>− Child sex × exposure: interaction p=0.014 (higher IRR for females).<br>Sensitivity analyses (excluding psychiatric parental comorbidities): adjusted IRR=1.98 (1.89–2.09).<br>Model fit: Deviance=456732.1; Pearson χ^2=458991.3; p<0.001.		
**25**	Santana et al., 2015, Brazil	Cross−sectional Retrospective Study	Offspring (n = 2,942) (Sao Paulo Megacity Mental Health Survey).	Parental psychiatric disorders (depression, panic disorder, GAD, substance abuse, antisocial personality).	Offspring exposed vs. unexposed to parental disorder.	Adolescence (13–19):<br>Suicidal ideation:<br>Depression OR=5.1 (1.9–13.7).<br>Antisocial personality OR=3.2 (1.3–8.0).<br>Suicidal attempt:<br>Depression OR=3.2 (1.3–8.0).<br>Panic disorder OR=3.8 (1.0–14.7).<br>GAD OR=3.3 (1.1–9.9).<br>Substance abuse OR=1.7 (1.0–3.0).<br>Number of disorders:<br>2 disorders OR ideation=2.7 (1.8–4.1), OR attempt=3.1 (2.0–4.8).<br>3+ disorders OR ideation=2.7 (1.7–4.3).	Discrete−time survival models; adjusted for sex, age, time−varying education, time−varying marital status, offspring psychopathology; significant interaction terms included; SUDAAN software; Wald χ^2; p<0.05	Parental depression and antisocial personality strongest predictors for adolescent suicidal ideation; depression, panic disorder, GAD, substance abuse significant for adolescent suicide attempts. Effects independent of offspring psychopathology. Recommends family−based prevention.

No.	Citation	Study Type	Participant Details	Exposure / Intervention Assessed	Group Allocation	Outcomes Evaluated	Analysis Approach	Major Findings
**26**	Scharpf et al., 2024, Tanzania	Cross−sectional Study	Family dyads (n = 230) recruited from three refugee camps in Tanzania (Nyarugusu, Nduta, Mtendeli), each consisting of one child aged 7–15 years (M=11.2, SD=2.6) and their primary caregiver (85% mothers, 15% fathers/other caregivers). Families were refugees from Democratic Republic of Congo and Burundi, living in camps between 1–20 years.	Parental psychopathology, parental suicidal behavior, exposure to war trauma, community violence.	Offspring with parental exposure vs without exposure.	Adolescents (7–15 yrs): Past−month suicidal ideation: 11.3%; suicide plan: 0.9%; suicide attempt: 0.9%; lifetime ideation: 24.7%; lifetime attempt: 4.3%. OR (adjusted odds ratio) for current suicide risk: Age: ORlower=2.20 (1.38–3.51); ORmod/high=3.03 (1.15–7.99). Externalizing problems: ORlower=1.56 (1.06–2.31); ORmod/high=3.03 (1.42–6.49). PTSD symptoms: ORlower=1.64 (1.05–2.57); ORmod/high=2.30 (1.02–5.16). Internalizing problems: ORmod/high=2.88 (1.33–6.26).	Multinomial logistic regression adjusting for sex, orphan status, school attendance, household size, income, social support, exposure to maltreatment and war trauma; Nagelkerke R^2=0.41; χ^2=83.24, p<0.001.	Adolescent suicidal ideation and attempts were significantly associated with older age, externalizing/internalizing problems, and PTSD symptoms. Parental psychopathology and suicidal behavior were associated with higher adolescent suicide risk indirectly via psychopathology. Prevention should target child mental health and family−level stressors.
**27**	Sheftall et al., 2021, USA	Cross−sectional, case−control analysis	Children aged 6–9 years (N = 146; PH+ n = 37, PH− n = 109), recruited from a large US metropolitan area; mean age 7.6	Parental history of suicide attempt (parent self−report)	Children with (PH+) vs. without (PH−) parental history of suicide attempt	Primary outcome: Child suicidal ideation (SI) (past month, child report); Secondary outcomes: Child depressive symptoms, anxiety, ADHD symptoms	Multivariate logistic regression models adjusted for child anxiety, ADHD, and other	Parental history of suicide attempt significantly increased risk for child suicidal ideation (OR = 4.14, 95% CI: 1.05–16.34, p < .05), even after controlling for child

No.	Citation	Study Type	Participant Details	Exposure / Intervention Assessed	Group Allocation	Outcomes Evaluated	Analysis Approach	Major Findings
			years; 56.8% male; oversampled for parental suicide attempt (PH+)				covariates; Odds Ratios (OR) with 95% CI reported	psychiatric symptoms. No significant association with depressive symptoms or ADHD in multivariate models. Findings underscore the need for early screening and intervention among children with parental suicide attempt history.
**28**	Takami Lageborn et al., 2024, Sweden	Population−based Cohort Study	Offspring (n = 24,788) of one parent with bipolar disorder; n = 247,880 matched controls from Swedish National Registers (1990–2013). Offspring followed until 18 years.	Parental bipolar disorder (ICD−8/9/10 codes), subtype (type 1/type 2), parental psychiatric comorbidity, parental suicide attempt, parental sex.	Offspring of parent(s) with bipolar disorder vs. offspring of parents without bipolar disorder	Suicide attempt: HR=2.47 (95% CI 2.18–2.80) → attenuated to HR=1.32 (95% CI 1.13–1.53) after adjustment for parental comorbidity & suicide attempt. Suicide: HR=3.75 (95% CI 1.65–8.51) → non−significant after adjustment: HR=1.34 (95% CI 0.55–3.27).<br>Additional outcomes: psychiatric diagnoses, psychotropic medication, accidents, victimization, low school grades, criminal behavior (full coefficient table S2/S3).	Cox proportional hazards models; logistic regression; adjustment for parental education, psychiatric comorbidity, parental suicide attempt; stratified by parental sex, bipolar subtype, dual vs. single parental diagnosis; Benjamini−Hochberg correction	Offspring of parents with bipolar disorder had increased risk of suicide attempt before 18 even after adjusting for parental psychiatric comorbidity and suicide attempt. Risk for suicide did not remain significant after adjustments. Highest risks observed in offspring of mothers with bipolar disorder, bipolar type 2, or both parents affected. Clinical implication: targeted early intervention needed for high−risk families.

No.	Citation	Study Type	Participant Details	Exposure / Intervention Assessed	Group Allocation	Outcomes Evaluated	Analysis Approach	Major Findings
							(α=0.05); cluster robust variance estimator; SAS 9.4.	
**29**	Tsypes et al., 2016, USA	Prospective Longitudinal Study	Mother–child pairs (n = 209) recruited from the community; children aged 8–14 years (M=10.9, SD=1.93); 87.6% Caucasian; mothers with MDD (n = 115) or no mood disorder (n = 94).	Maternal MDD diagnosis; maternal suicide attempt history (SBQ–R); cognitive vulnerabilities (hopelessness, global self–worth, rumination).	Children of depressed mothers vs. children of nondepressed mothers.	Suicidal ideation:<br>Baseline prevalence: Mom MDD/Child SI=37 (32.2%); Mom MDD/No Child SI=78 (67.8%); Controls=0%.<br>Incidence new SI over 2 years: 27 new cases.<br>Cox model predictors of first onset SI:<br>Hopelessness: Wald=5.69, OR=1.48 (95% CI 1.03–2.00), p=0.01.<br>Global Self–Worth: Wald=5.28, OR=0.45 (95% CI 0.22–0.95), p=0.01.<br>Effects remained significant after adjusting for child depression and MDD onset (HSC: OR=1.47, p=0.02; SPPC–GSW: OR=0.47, p=0.02).<br>Brooding rumination not predictive longitudinally (p=0.67).	Cox proportional hazards regression; adjusted for child depressive symptoms, MDD onset, maternal suicide attempt; Kaplan–Meier survival curves; ORs with 95% CIs; p<.05 threshold.	Hopelessness and low global self–worth significantly predicted first onset of suicidal ideation in children of depressed mothers over 2–year follow–up, independent of child depressive symptoms, maternal suicide attempt history. Brooding rumination was elevated cross–sectionally but not predictive. Suggests targeting hopelessness and self–worth in suicide prevention for at–risk youth.
**30**	Zhu et al., 2023, UK	Prospective, population–based	n = 12,520; children followed from ages 3 to 17	Observational – longitudinal trajectories of	Comparison of four LCGA groups: (1) low	Proportion with self–reported suicide attempt at age 17; self–harm at ages 14 and 17;	Parallel–process latent class growth analysis	Co–occurring trajectories of maternal psychological distress and child

No.	Citation	Study Type	Participant Details	Exposure / Intervention Assessed	Group Allocation	Outcomes Evaluated	Analysis Approach	Major Findings
		longitudinal cohort (Millennium Cohort Study, MCS)	years; nationally representative UK sample; biological mothers and fathers included	parental psychological distress (K6, mothers/fathers) and child internalizing/externalizing problems (SDQ)	symptoms, (2) moderate child symptoms, (3) notable paternal symptoms, (4) co−occurring high maternal and child symptoms	suicide attempt prevalence in co−occurring mother–child group: 14%, other groups: 5–9%	(LCGA); Bolck−Croon−Hagenaars (BCH) method for distal outcomes; Wald tests for group comparisons; adjustments for clustering, stratification, survey weighting, and missing data (FIML)	internalizing/externalizing problems significantly predict increased risk of adolescent suicide attempt and self−harm. Intervention programs should target both children and parents simultaneously.
**31**	Zubrick et al., 2016, Australia	National cross−sectional, population−based survey	Adolescents aged 12–17 years (n = 2,653), nationally representative sample (Australia, 2013–2014)	Observational – Parental and adolescent mental disorders, family structure, family functioning	Adolescents with vs. without any parent−reported or self−reported mental disorder (MDD, anxiety, ADHD, CD)	12−month prevalence: suicidal ideation (7.5%, 95% CI: 6.5–8.6%), suicide plan (5.2%, 95% CI: 4.3–6.1%), suicide attempt (2.4%, 95% CI: 1.8–3.0%). Strongest predictor: adolescent MDD and suicide attempt (OR = 16.8, 95% CI: 8.8–32.1, p < .001); family structure, poor family functioning, and low income also increased risk.	Univariate and multivariate logistic regression; survey weighting; 95% CIs; adjustment for sociodemographic and family covariates; SAS SURVEYLOGISTIC procedures	Adolescent mental disorders, especially major depressive disorder, are the strongest predictors of suicidal ideation and suicide attempt. Family structure (single−parent family), poor family functioning, and low income increase risk independently. Recommends mental health interventions and family support for vulnerable groups.

No.	Citation	Study Type	Participant Details	Exposure / Intervention Assessed	Group Allocation	Outcomes Evaluated	Analysis Approach	Major Findings
References: Complete citations are included in the main reference list of the manuscript.								

Table S5. Critical Appraisal of Observational Studies: Newcastle–Ottawa Scale Ratings and GRADE Evidence Certainty.

No.	Study	Design	GRADE Evidence Level (Assessment/ Rationale)	NOS: Selection (Score/Notes)	NOS: Comparability (Score/Notes)	NOS: Exposure/ Outcome (Score/ Notes)	NOS: Overall Rating (Score/Notes)
**1**	Barzilay et al., 2022, USA	Cross–sectional analysis (secondary	Moderate (Well–powered, large, diverse sample with robust genetic and	3/4 (Representative cohort of US adolescents, clear definition of exposure	1/2 [Adjusted for key confounders (age, sex, ancestry); additional confounders possible	2/3 [Validated tools (KSADS–5) for suicidality assessment; polygenic score	6/9 [Good quality; primary limitations due to study design

No.	Study	Design	GRADE Evidence Level (Assessment/ Rationale)	NOS: Selection (Score/Notes)	NOS: Comparability (Score/Notes)	NOS: Exposure/ Outcome (Score/ Notes)	NOS: Overall Rating (Score/Notes)
		analysis of ABCD cohort)	phenotypic data; limitations due to cross–sectional design, potential residual confounding, and underreporting of suicide attempts.)	(parental history, polygenic risk), but limited by reliance on self–report for outcomes.)	(socioeconomic, psychiatric comorbidity.]	construction rigorous; some limitations in follow–up and outcome ascertainment.]	(cross–sectional) and residual confounding.]
**2**	Brent et al., 2015, USA	Prospective Cohort	High (Well–designed longitudinal cohort, large sample, detailed control for confounders, clear outcome definition, low attrition bias.)	4/4 (Representativeness of exposed cohort, selection of non–exposed cohort, ascertainment of exposure, demonstration that outcome not present at start.)	2/2 (Controlled for key confounders: age, sex, mood disorder, prior suicide attempt.)	3/3 (Independent blind assessment, adequate follow–up, clear outcome definition.)	9/9 (Maximum score: High methodological quality.)
**3**	Brent et al., 2019, USA	Pharmacoepide miologic cohort study using large–scale health claims data	High (Large, national–level dataset with rigorous matching; outcomes clearly defined; minimal risk of bias due to robust propensity score matching and adjustment for multiple confounders.)	4/4 (Exposed and unexposed cohorts drawn from the same large, representative claims database; clear definition of exposure; inclusion criteria well defined; no evidence of selection bias.)	2/2 (Matching and statistical adjustment for key demographic and clinical confounders (parental and child psychiatric diagnoses, parental substance use, age, sex, geographic region.)	3/3 [Ascertainment of exposure (medical claims for prescriptions) and outcome (suicide attempt diagnosis) both robust; follow–up was sufficient for outcomes to occur; linkage between parent and child well established.]	9/9 (Maximum NOS score; study design and reporting are rigorous, supporting the highest quality rating.)

No.	Study	Design	GRADE Evidence Level (Assessment/ Rationale)	NOS: Selection (Score/Notes)	NOS: Comparability (Score/Notes)	NOS: Exposure/ Outcome (Score/ Notes)	NOS: Overall Rating (Score/Notes)
**4**	Chae et al., 2020, Korea	Cross−sectional, population−based analysis using KNHANES (Korean National Health and Nutrition Examination Survey) data	Moderate (Nationally representative, large sample; robust adjustment for multiple confounders; limitations due to cross−sectional design, self−reported suicidal ideation, and absence of causality inference.)	3/4 [Representative sample of Korean adolescents; clear inclusion criteria and exposure (parental suicidal ideation); some risk of bias from self−reported variables.]	2/2 (Adjusted for major confounders including age, sex, depressive symptoms, stress, and socioeconomic status.)	2/3 [Outcome (adolescent suicidal ideation) based on standardized survey question; statistical analysis robust; limited by cross−sectional ascertainment and self−report.]	7/9 (Good methodological quality, with primary limitation due to cross−sectional design and risk of recall bias.)
**5**	Chan et al., 2018, New Zealand	Cross−sectional, nationally representative survey (Youth'12)	Moderate (Large sample, nationally representative, robust multistage random sampling; cross−sectional design limits causal inference; self−report introduces recall bias; key confounders statistically adjusted.)	3/4 [Random selection of schools and students ensures representativeness; large, national cohort; clearly defined exposure (suicide attempt or death in friends/family); some reliance on self−report.]	1/2 (Adjusted for key confounders, including age, sex, ethnicity, socioeconomic status, and low mood; possible residual confounding by unmeasured variables such as trauma or psychiatric comorbidity.)	2/3 [Use of validated survey tools and standardized questions; outcome (suicide attempt and non−suicidal self−injury, NSSI) based on self−report; outcome ascertainment is robust but lacks clinical verification.]	6/9 (Good methodological quality; limitations stem from cross−sectional design, reliance on self−report, and inability to confirm outcomes via clinical records.)
**6**	Christiansen et al., 2024, Denmark	Nationwide registry−based cohort study	High (Large, representative population; registry data reduces selection and recall bias; minimal loss to follow−up; detailed	4/4 (Complete national cohort, registry linkage, clear exposure/outcome definitions, prospective follow−up)	2/2 (Adjusted for major confounders: sex, age, parental psychiatric history, income, parental age at birth)	3/3 (Registry−based exposure/outcome ascertainment; robust definitions; longitudinal follow−up)	9/9 (Excellent quality; minor risk from potential unmeasured confounding)

No.	Study	Design	GRADE Evidence Level (Assessment/ Rationale)	NOS: Selection (Score/Notes)	NOS: Comparability (Score/Notes)	NOS: Exposure/ Outcome (Score/ Notes)	NOS: Overall Rating (Score/Notes)
			exposure and outcome ascertainment; risk of unmeasured confounding due to lack of psychosocial covariates)				
**7**	Cluver et al., 2015, South Africa	Prospective cohort study	Moderate (Large, population−based sample of South African adolescents; robust longitudinal design; high retention rate; validated outcome measures; key limitations include reliance on self−reported adversities and mental health symptoms, potential unmeasured confounding, and 1−year follow−up duration.)	4/4 (Random selection from census enumeration areas; high participation and retention; clear definitions of exposures (adverse childhood experiences, ACE); thorough baseline characterization.)	2/2 (Analyses adjusted for major sociodemographic confounders (age, gender, location, socioeconomic status); further adjusted for baseline suicidality.)	2/3 [Outcomes assessed using the MINI International Neuropsychiatric Interview for Children and Adolescents (MINI KID); validated and reliable instruments; exposure (ACE) assessment via multiple validated scales; outcomes based on self−report; 1−year follow−up limits long−term ascertainment.]	8/9 (High methodological quality; minor limitations due to self−report bias and possible unmeasured confounders; excellent sampling and analytical approach.)
**8**	Easey et al., 2019, UK	Population−based longitudinal cohort study [Avon Longitudinal Study of Parents	Moderate (Large, representative birth cohort, comprehensive longitudinal follow−up; robust statistical methods including	4/4 [Selection of exposed and non−exposed based on clear criteria, use of a well−defined cohort (ALSPAC),	2/2 (Fully adjusted for key confounders: maternal age, social class, income, gestational age, prenatal alcohol/tobacco	3/3 [Validated outcome measures for suicide attempts and psychiatric disorder using established questionnaires (Child	9/9 (High methodological quality; minimal risk of bias; strengths include the use of prospective data, mediation analysis, and

No.	Study	Design	GRADE Evidence Level (Assessment/ Rationale)	NOS: Selection (Score/Notes)	NOS: Comparability (Score/Notes)	NOS: Exposure/ Outcome (Score/ Notes)	NOS: Overall Rating (Score/Notes)
		and Children (ALSPAC)]	imputation for missing data; limitations include potential for residual confounding and attrition bias.)	high representativeness, and reliable record linkage.]	exposure; comparison across birth order categories is appropriate.)	and Adolescent Self−harm in Europe, CASE, study and Development and Well−Being Assessment, DAWBA]; clear, objective ascertainment of exposure and outcome; longitudinal data collection with mediation analysis.]	detailed adjustment for confounding variables.)
**9**	Giletta et al., 2015, China	Prospective cohort (multiwave, school−based sample)	Moderate (Strengths include repeated, multi−informant assessment and robust analytic methods. Limitations are due to lack of parental predictors, focus on peer factors, and absence of population representativeness.)	3/4 (Cohort of 10th grade Chinese adolescents from two schools; clear inclusion criteria and outcome definitions; some risk of selection bias due to school−based recruitment.)	1/2 (Adjusted for important confounders, including gender and baseline depressive symptoms. Other confounders possible, e.g., family background and socioeconomic status.)	2/3 [Validated instruments for nonsuicidal self−injury (NSSI), suicide ideation, and suicide attempts (SA); exposure measurement robust, but some limitations in longitudinal follow−up completeness.]	6/9 (Overall good methodological quality, but lacks direct parental predictor assessment as required by the review focus.)
**10**	Goldston et al., 2016, USA	Longitudinal cohort study	Moderate [Strengths include prospective design, long follow−up period, repeated assessments, and use of	3/4 (Sample: Consecutive admissions of adolescents aged 12–19 years to a	2/2 (Analyses adjusted for key demographic and clinical confounders, including psychiatric comorbidity,	2/3 [Exposure: Parental history of suicidal behavior assessed; Outcome: STBs measured using	7/9 (High−quality longitudinal cohort; primary limitations relate to sample representativeness and

No.	Study	Design	GRADE Evidence Level (Assessment/ Rationale)	NOS: Selection (Score/Notes)	NOS: Comparability (Score/Notes)	NOS: Exposure/ Outcome (Score/ Notes)	NOS: Overall Rating (Score/Notes)
			validated instruments for suicidal thoughts and behaviors (STBs); however, limited generalizability due to recruitment from a single psychiatric inpatient facility, and some potential for bias as assessments were not blinded.]	psychiatric inpatient unit, well−defined inclusion/exclusion criteria, high retention; limited by lack of population−based recruitment.)	age, sex, and abuse history.)	structured clinical interviews (Interview Schedule for Children and Adolescents (ISCA), Follow−Up Interview Schedule for Adults (FISA)); high interrater reliability; some limitations in generalizability.]	potential unmeasured confounding.)
**11**	Halonen et al., 2019, Finland	Nationwide cohort study (register−based; 1987 Finnish Birth Cohort)	High (Large, population−based national cohort with nearly complete follow−up, robust use of administrative health and social welfare registers, and well−defined exposure and outcome; some limitations due to residual confounding and lack of data on some individual−level behavioral risk factors.)	4/4 (Complete, representative national birth cohort; clear exposure definition using register−based diagnoses for parental mental disorders.)	2/2 (Adjusted for major confounders including offspring's sex, socioeconomic status, and geographic region; subgroup and sensitivity analyses reported.)	3/3 (Objective and validated outcome measures—work disability due to depressive or anxiety disorders—ascertained via national registers; robust mediation analysis for pathways.)	9/9 (Excellent quality; comprehensive data, rigorous design, and statistical analysis. Limitations minimal and transparently discussed.)
**12**	Hammerton et al., 2015, UK	Prospective longitudinal cohort study	Moderate (Large population−based cohort, well−defined	4/4 [Cohort is representative of the UK population;	2/2 (Comprehensive adjustment for sociodemographic and	2/3 [Validated instruments for exposure and	8/9 (High quality: only minor limitations due to self−report outcomes

No.	Study	Design	GRADE Evidence Level (Assessment/ Rationale)	NOS: Selection (Score/Notes)	NOS: Comparability (Score/Notes)	NOS: Exposure/ Outcome (Score/ Notes)	NOS: Overall Rating (Score/Notes)
		(ALSPAC: Avon Longitudinal Study of Parents and Children)	exposure to maternal depression using repeated measures, strong mediation analysis; limitations include potential residual confounding, attrition, and reliance on self–report for some outcomes.)	maternal depression assessed using validated Edinburgh Postnatal Depression Scale (EPDS) at 10 timepoints; clearly defined exposure and outcomes; minimal selection bias.]	familial confounders, including maternal psychiatric history, socioeconomic status, and smoking during pregnancy.)	outcomes: Development and Well–Being Assessment (DAWBA) for offspring psychopathology, self–report for suicidality; some outcomes rely on self–report and parental report, introducing potential bias.]	and potential for residual confounding.)
**13**	Hammerton et al., 2016, UK	Prospective longitudinal cohort study (ALSPAC: Avon Longitudinal Study of Parents and Children)	Moderate (Large, population–based birth cohort with longitudinal assessment, robust analytical methods, and validated measures. Strengths include mediation modeling and sensitivity analyses. Limitations: selective attrition, reliance on self–report for some measures, and	4/4 [Representative cohort, clear definition and repeated measurement of exposure (maternal depression trajectories), use of validated tools (Edinburgh Postnatal Depression Scale), prospective data collection.]	2/2 (Adjusted for key confounders [child gender, housing tenure, marital status, maternal education, smoking in pregnancy, family history of depression, maternal psychiatric disorder before pregnancy]. Additional confounders possible but well–addressed.)	3/3 [Validated outcome measures (self–reported suicidal ideation, Development and Well–Being Assessment (DAWBA), comprehensive follow–up, outcome assessors not independent but methods robust.]	9/9 (High quality: maximum score; primary strengths are prospective design, detailed confounder control, and rigorous outcome/exposure measurement.)

No.	Study	Design	GRADE Evidence Level (Assessment/ Rationale)	NOS: Selection (Score/Notes)	NOS: Comparability (Score/Notes)	NOS: Exposure/ Outcome (Score/ Notes)	NOS: Overall Rating (Score/Notes)
			potential unmeasured confounding.)				
**14**	Han et al., 2023, Korea	Cross–sectional, matched analysis of a national survey cohort	Moderate (Large, nationally representative sample; robust propensity score matching; limitations due to cross–sectional design, use of single–item measures for key variables, and potential for residual confounding.)	3/4 (Representative national cohort, clear definition of exposure (parental diagnosed depression, depressive mood, suicidal ideation); minor limitation due to single–item measurement.)	1/2 (Propensity score matching on demographic variables; other possible confounders—family communication, peer influence—could not be included.)	2/3 (Use of validated national survey items; standardized outcomes (suicidal ideation, help–seeking behavior); but limited by single–item questions and self–report.)	6/9 (Good quality; main weaknesses are cross–sectional design and measurement limitations, but strengths include large sample and careful matching.)
**15**	Jeong et al., 2020, Korea	Cross–sectional analysis [secondary analysis of national Korean Youth Risk Behavior Web–based Surveys (KYRBS)]	Moderate (Exceptionally large, nationally representative sample over 11 years, rigorous data collection, high response rates. Main limitations: cross–sectional design, reliance on self–reported data, potential recall and reporting biases, and inability to assess causality.)	4/4 (Large, stratified, and representative national sample of Korean adolescents; well–defined inclusion and exclusion criteria; high participation rate; robust sampling procedures.)	2/2 [Adjusted for multiple relevant confounders: sex, grade, socioeconomic status (SES), academic achievement, living arrangement, behavioral factors, and psychological variables in multivariable logistic regression.]	3/3 (Validated survey instruments, standardized outcome definitions for depression, suicidal ideation, and suicide attempts, and consistent data collection procedures across years.)	9/9 (Excellent methodological quality; main limitation is the cross–sectional nature, which limits causal inference.)

No.	Study	Design	GRADE Evidence Level (Assessment/ Rationale)	NOS: Selection (Score/Notes)	NOS: Comparability (Score/Notes)	NOS: Exposure/ Outcome (Score/ Notes)	NOS: Overall Rating (Score/Notes)
**16**	Kawabe et al., 2016, Japan	Cross–sectional, population–based survey	Moderate [The study used a representative community sample of junior high school students and their caregivers, with validated instruments: General Health Questionnaire (GHQ) and Profile of Mood States (POMS). Limitations include cross–sectional design, exclusion of some participants due to missing data, and potential for unmeasured confounding variables.]	3/4 (Representative sample of adolescents from a defined region, high response rate, and validated exposure assessment. However, some participants were excluded due to missing caregiver data, which could introduce selection bias.)	1/2 (Analyses adjusted for key covariates such as sex and grade; other potential confounders—such as socioeconomic status and family structure—were not controlled.)	2/3 [Exposure and outcomes measured with validated tools (GHQ, POMS), but outcomes were assessed at a single time point and relied on self–report, which may introduce reporting bias.]	6/9 (Overall good methodological quality; major limitations arise from cross–sectional design, exclusion of some participants, and lack of adjustment for certain confounders.)
**17**	Kendler et al., 2020, Sweden	Population–based cohort study (using national Swedish registers and extended adoption design)	High (Large national sample; robust use of adoption design to parse genetic and environmental transmission; comprehensive register linkage; outcomes defined by clinical diagnoses; limitations	4/4 (Excellent selection: comprehensive national registers covering multiple decades; objective ascertainment of exposure and outcome through health, mortality, and	2/2 (Adjusted for numerous confounders: parental psychiatric disorders, substance use, socioeconomic variables, child sex, and age; adoption design further separates genetic and environmental effects.)	3/3 [Outcomes—suicide attempt and suicide death—ascertained from validated Swedish Hospital Discharge and Mortality Registers; use of ICD codes (International Classification of	9/9 (Outstanding methodological quality; comprehensive adjustment for confounders; minimal risk of bias; results highly generalizable within registry settings.)

No.	Study	Design	GRADE Evidence Level (Assessment/ Rationale)	NOS: Selection (Score/Notes)	NOS: Comparability (Score/Notes)	NOS: Exposure/ Outcome (Score/ Notes)	NOS: Overall Rating (Score/Notes)
			due to registry−based ascertainment and possible misclassification, but mitigated by validation studies and broad time span.)	census registers; robust family linkage; clear definition of family types.)		Diseases); extensive follow−up and careful linkage; minimal loss to follow−up.]	
**18**	Lee et al., 2021, Korea	Cross−sectional, population−based survey (Korea National Health and Nutrition Examination Survey, KNHANES)	Moderate (Representative, large−scale national sample; robust assessment of parental and adolescent risk factors; limited by cross−sectional design and self−report measures; causal inference not possible.)	3/4 (Representative nationwide sample of adolescent girls and their parents; well−defined inclusion criteria; some limitations due to self−reporting and non−institutionalized population.)	1/2 (Adjusted for key demographic and clinical variables, including socioeconomic status and parental education; residual confounding possible for unmeasured variables.)	2/3 (Validated and structured assessment of suicidal behaviors using standardized questions; limited by lack of longitudinal follow−up and reliance on self−report for both parents and adolescents.)	6/9 (Good quality; strengths include large, representative sample and analytic rigor; limitations relate to study design and potential for recall and reporting bias.)
**19**	Logeswaran et al., 2025, Denmark	Self−controlled case series (population−based, registry study)	**Moderate** (Large, nationwide sample, use of robust national registers with validated outcome coding, temporal relationship established. Limitation: SCCS only includes those with an outcome [not the full bereaved cohort], possible	3/4 (Representative national cohort; complete registry linkage minimizes selection bias; clear and objective exposure definition. Limited by restriction to cases with outcome.)	1/2 (Adjusted for major time−varying confounders: age, marital status, household income. Lacked adjustment for other possible confounders, e.g. detailed psychiatric comorbidity.)	2/3 (Use of validated national registers for suicide/self−harm outcomes; clear exposure/outcome ascertainment; limited by lack of self−report/primary care events and by follow−up window.)	6/9 (Good quality observational evidence; primary limitations: sample restricted to those with outcomes [limits generalizability], potential residual confounding, and outcome ascertainment restricted to

No.	Study	Design	GRADE Evidence Level (Assessment/ Rationale)	NOS: Selection (Score/Notes)	NOS: Comparability (Score/Notes)	NOS: Exposure/ Outcome (Score/ Notes)	NOS: Overall Rating (Score/Notes)
			residual confounding, under–ascertainment of self–harm not requiring secondary care, limited adjustment for psychiatric comorbidities, and limited power for subgroup/interaction analysis)				severe/self–harm in secondary care.)
**20**	Maguire et al., 2022, Northern Ireland	Population–wide cohort, data linkage study	High [Large, representative population–based cohort, robust data linkage, comprehensive adjustment for confounders, use of official death records for suicide outcome; limitations include self–reported mental health (MH), possible reporting bias, and simultaneous measurement of parent and offspring MH status.]	4/4 [Entire Northern Ireland population census; clear definition and identification of exposure (parental mental health, MH); reliable identification of parent–child households; outcome (suicide) based on official mortality records.]	2/2 (Extensive adjustment for potential confounders including age, gender, physical illness, socioeconomic status, and offspring's own MH. Clustered standard errors accounted for family units.)	3/3 [Objective outcome ascertainment (official death records for suicide, census for MH); well–validated measurement approach; appropriate follow–up period for mortality outcome.]	9/9 (Excellent methodological quality; large, representative cohort; comprehensive adjustment for confounders; reliable outcome ascertainment; primary limitation is possible reporting bias for self–reported parental MH and inability to determine temporal sequence of exposures.)

No.	Study	Design	GRADE Evidence Level (Assessment/ Rationale)	NOS: Selection (Score/Notes)	NOS: Comparability (Score/Notes)	NOS: Exposure/ Outcome (Score/ Notes)	NOS: Overall Rating (Score/Notes)
**21**	Mok et al., 2016, Denmark	Population−based cohort study	High [Exceptionally large national cohort (n = 1,743,525) with prospective registry linkage and virtually complete follow−up; exposures and outcomes based on clinical records; analyses robust, including adjustment for confounders such as socioeconomic status (SES); some limitations due to unmeasured confounders, such as family criminal history and child abuse.]	4/4 [Inclusion of entire Danish national birth cohort; exposures (parental psychiatric diagnoses and suicide attempt) are objectively measured via national registers; outcome measures (offspring suicide attempt and violent offending) are also registry−based.]	2/2 (Adjusted for offspring age, sex, calendar year, SES, and tested interactions; extensive adjustment for confounders.)	3/3 (Ascertainment of exposures and outcomes through validated, objective national registers; complete follow−up and large sample size minimize attrition bias.)	9/9 (Outstanding methodological quality for an observational study; limitations are minimal and relate only to unmeasured or unavailable confounders.)
**22**	O'Reilly et al., 2020, Sweden	Population−based sibling cohort study (register−based)	High (Exceptionally large, population−based national cohort using Swedish registers; robust statistical approaches including behavior genetic modeling and fixed−effects Cox regression; careful	4/4 (Representative nationwide cohort of over 2.7 million offspring; clear and valid exposure definitions using national registers; strong ascertainment of outcomes; high completeness of follow−up.)	2/2 (Rigorous adjustment for familial and individual−level confounders, including parental severe mental illness, substance use, criminal conviction, and socioeconomic factors; use of cousin−comparison and sibling fixed−effects	3/3 [Exposure and outcome data from high−quality national health and mortality registers; outcome (suicidal behavior) precisely defined as hospital−registered suicide attempt or death by suicide; comprehensive and	9/9 (Excellent methodological quality; all domains at maximum scores; primary limitations relate to potential misclassification of non−hospitalized suicide attempts and register−based data constraints.)

No.	Study	Design	GRADE Evidence Level (Assessment/ Rationale)	NOS: Selection (Score/Notes)	NOS: Comparability (Score/Notes)	NOS: Exposure/ Outcome (Score/ Notes)	NOS: Overall Rating (Score/Notes)
			adjustment for multiple measured and unmeasured confounders; main limitations relate to reliance on register data for suicidal behavior, possible under−ascertainment of non−hospitalized suicide attempts, and inability to fully capture all environmental confounding.)		models to account for unmeasured familial confounding.)	systematic data linkage and follow−up.]	
**23**	Ortin−Peralta et al., 2024, USA	Longitudinal, population−based cohort [Adolescent Brain Cognitive Development (ABCD) study]	Moderate [Large and demographically diverse US cohort; robust repeated assessments; adjustment for multiple confounders; limitations include inability to separate parental suicide attempt (SA) from suicide death (SD), and possible underestimation of	4/4 (Population−based representative sample; well−defined inclusion and exclusion criteria; reliable ascertainment of parental suicide attempt/suicide death and child outcomes.)	2/2 (Adjusted for major confounders, including child's sex, race/ethnicity, family structure, internalizing problems, and financial adversity.)	3/3 [Validated outcome measures: Kiddie Schedule for Affective Disorders and Schizophrenia−Present and Lifetime Version (K−SADS−PL); comprehensive longitudinal follow−up; minimal loss to follow−up; rigorous analytic approach.]	9/9 (High quality; strengths include representative design, robust methods, and comprehensive outcome measurement; minor limitations related to combining parental suicide attempt and suicide death, and reliance on self−report.)

No.	Study	Design	GRADE Evidence Level (Assessment/ Rationale)	NOS: Selection (Score/Notes)	NOS: Comparability (Score/Notes)	NOS: Exposure/ Outcome (Score/ Notes)	NOS: Overall Rating (Score/Notes)
			exposures due to caregiver reporting.]				
**24**	Ranning et al., 2022, Denmark	Nationwide prospective cohort study (register linkage)	High [Population–based, prospective cohort with 4.4 million children, comprehensive register linkage, robust adjustment for multiple confounders, minimal attrition; primary limitations relate to under–recording of suicide attempts (SA), and possible residual confounding due to register–based measurement.]	4/4 [Entire national population cohort; well–defined exposure (parental suicide attempt, SA); use of validated Danish registers; very low risk of selection bias.]	2/2 (Comprehensive adjustment for potential confounders, including parental psychiatric disorders, socioeconomic status, parental victimization, family structure, and child–level covariates.)	3/3 (Exposure and outcomes ascertained using national registers with ICD codes, prospective follow–up; outcome defined as first SA in offspring; minimal misclassification; detailed timing of exposure and outcome considered.)	9/9 (Excellent methodological quality; minimal risk of bias due to study design, sample representativeness, confounder adjustment, and ascertainment methods. Residual confounding possible but unlikely to materially affect conclusions.)
**25**	Santana et al., 2015, Brazil	Cross–sectional, population–base d survey (São Paulo Megacity Mental Health Survey)	Moderate (Large, representative sample of adults from a major Brazilian metropolitan area; rigorous measurement tools, but design is cross–sectional, and reliance on retrospective self–report introduces	3/4 [Representative population sample, validated diagnostic interview (WMH–CIDI: World Mental Health Survey Composite International Diagnostic Interview), clear definitions of both exposure	1/2 (Adjusted for major demographic confounders, including sex, age, education, marital status, and comorbidity in multivariate models. However, there is potential for residual confounding, especially regarding unmeasured	2/3 (Validated tools for both exposure and outcome; exposure determined by offspring report, outcome by structured interview; some risk of misclassification and recall bias due to retrospective design.)	6/9 (Good methodological quality; main limitations are due to cross–sectional and retrospective design, possible recall bias, and limited adjustment for all possible confounders.)

No.	Study	Design	GRADE Evidence Level (Assessment/ Rationale)	NOS: Selection (Score/Notes)	NOS: Comparability (Score/Notes)	NOS: Exposure/ Outcome (Score/ Notes)	NOS: Overall Rating (Score/Notes)
			recall bias. Parental psychopathology and suicidal behaviors are measured by standardized interviews, but adolescent−specific data are not isolated.)	(parental psychopathology) and outcome (offspring suicidality), but retrospective assessment limits temporality.]	socioeconomic and family variables.)		
**26**	Scharpf et al., 2024, Tanzania	Cross−sectional study (community−based survey of Burundian refugee families in three Tanzanian camps)	Moderate [Well−conducted representative survey with robust data collection; use of validated measures (MINI, MINI−KID) for suicidality and mental health. Limitations: cross−sectional design, self−report, risk of recall/social desirability bias, limited to refugee camp setting.]	3/4 (Representative random sampling of children and parents, clear eligibility and structured interviews; however, outcome relies on self−report and ad hoc translation for some terms.)	1/2 (Adjusted for age, sex, household, exposure to trauma, and other sociodemographic confounders; residual confounding possible—some variables not controlled, such as intimate partner violence or peer relationships.)	2/3 [Validated instruments for suicidality and mental health (MINI, MINI−KID, PTSD Checklist, SDQ), outcome assessment well described; main limitation is lack of longitudinal follow−up and reliance on participant recall.]	6/9 (Good quality, with primary limitations due to cross−sectional design, possible unmeasured confounders, and setting−specific generalizability.)
**27**	Sheftall et al., 2021, USA	Cross−sectional case−control study (USA)	**Moderate** (Well−designed cross−sectional study, good use of standardized outcome measures, moderate sample size; main	3/4 (Representative clinical sample, exposure clearly defined, but single−center limits generalizability)	1/2 (Adjusted for main child psychiatric confounders, but not all potential family/social confounders)	2/3 (Validated outcome measures, but relies partly on self−/parent report; no longitudinal follow−up)	6/9 (Good quality; key limits: cross−sectional, selection bias)

No.	Study	Design	GRADE Evidence Level (Assessment/ Rationale)	NOS: Selection (Score/Notes)	NOS: Comparability (Score/Notes)	NOS: Exposure/ Outcome (Score/ Notes)	NOS: Overall Rating (Score/Notes)
			limitations: cross–sectional design, possible selection bias, and residual confounding.)				
**28**	Takami Lageborn et al., 2024, Sweden	Nationwide population–based cohort study	High (Large sample size, robust national registry linkage, comprehensive outcome assessment; results consistently adjusted for confounders; limitations include absence of some socioeconomic factors and restriction to Swedish registers.)	4/4 (Complete national cohort, robust exposure definition (parental bipolar disorder confirmed by at least two ICD–coded diagnoses), minimal selection bias, inclusion/exclusion well–described.)	2/2 (Adjusted for major confounders: parental education and psychiatric comorbidity; additional subgroup analyses by parental sex and bipolar subtype increase rigor.)	3/3 (Outcomes from validated Swedish national registers, objective and standardized measurement of psychiatric, somatic, social, and mortality outcomes; detailed outcome definitions; long follow–up.)	9/9 (Excellent quality; minimal risk of bias due to study design, confounder adjustment, and rigorous methodology.)
**29**	Tsypes et al., 2016, USA	Prospective, longitudinal cohort study	Moderate [Well–structured, multi–wave design with both maternal and child clinical interviews; comprehensive assessment of cognitive vulnerabilities and suicidal ideation (SI); limited by relatively	3/4 [Community–based sample; clear definitions of maternal depression and SI; assessment via validated diagnostic interviews [Structured Clinical Interview for DSM–IV Axis I Disorders (SCID–I);	1/2 [Adjusted for baseline depressive symptoms in children, as well as maternal suicide attempt (SA) history; did not adjust for all possible confounders such as socioeconomic status (SES) or comorbid	2/3 [Standardized, validated measures for exposure (maternal depression, cognitive vulnerability questionnaires) and outcome (SI via K–SADS–PL); outcome assessed longitudinally over 2 years; some reliance	6/9 (Good methodological quality, primary limitations are small sample size, regional sample, and some residual confounding.)

No.	Study	Design	GRADE Evidence Level (Assessment/ Rationale)	NOS: Selection (Score/Notes)	NOS: Comparability (Score/Notes)	NOS: Exposure/ Outcome (Score/ Notes)	NOS: Overall Rating (Score/Notes)
			small sample size, single geographic area, and some reliance on self–report.]	Schedule for Affective Disorders and Schizophrenia for School–Age Children–Present and Lifetime Version (K–SADS–PL)]; potential limitations in sample representativeness due to regional recruitment.]	psychiatric disorders in mothers.]	on self–report for cognitive vulnerabilities; attrition not systematically reported.]	
**30**	Zhu et al., 2023, UK	Longitudinal cohort study (Millennium Cohort Study)	Moderate (Large, nationally representative UK cohort; 14 years of follow–up; robust trajectory modeling; main limitations include reliance on parent–reported child outcomes, lack of repeated youth self–reports, and possible residual confounding despite careful adjustment.)	4/4 [Representative birth cohort; clear inclusion/exclusion criteria; valid, widely–used measures for exposure (Kessler Psychological Distress Scale, K6); large sample size.]	2/2 (Adjustment for key confounders, including demographic variables; analysis stratified and weighted; additional sensitivity and subgroup analyses conducted.)	3/3 [Repeated outcome assessments using validated measures (Strengths and Difficulties Questionnaire, SDQ); suicide attempts and self–harm measured with standard, widely–used survey items; outcomes reported at multiple timepoints; some reliance on self–report.]	9/9 (High methodological quality; strengths include long–term follow–up, advanced trajectory modeling, and comprehensive confounder adjustment; main limitation is reliance on parent–reported child symptoms up to age 14 and potential shared rater bias.)

No.	Study	Design	GRADE Evidence Level (Assessment/ Rationale)	NOS: Selection (Score/Notes)	NOS: Comparability (Score/Notes)	NOS: Exposure/ Outcome (Score/ Notes)	NOS: Overall Rating (Score/Notes)
**31**	Zubrick et al., 2016, Australia	Cross−sectional (National survey)	Moderate (Large, representative Australian sample with robust measures of suicidal behaviors and mental disorders; limitations due to cross−sectional design, reliance on self−report, and lack of temporal directionality between exposure and outcome.)	4/4 [Representative national sample, clear inclusion/exclusion criteria, high response rate, validated instruments such as the Diagnostic Interview Schedule for Children Version IV (DISC−IV) and Youth Risk Behavior Surveillance System (YRBSS).]	1/2 (Adjusted for key sociodemographic confounders—age, sex, family structure, socioeconomic status; however, possible residual confounding from unmeasured variables.)	2/3 [Suicidal behaviors measured using validated self−report tools; mental disorders assessed with standardized instruments (DISC−IV); however, outcome measurement based solely on self−report without external validation or follow−up.]	7/9 (High quality; primary limitations relate to cross−sectional design and reliance on self−report data.)

Notes:

- GRADE Evidence Level: Assesses the overall certainty of evidence produced by the study, based on factors such as study design, risk of bias, inconsistency, indirectness, and imprecision. Rated as High, Moderate, Low, or Very Low. Higher ratings indicate more reliable and trustworthy evidence.
- NOS Selection: Evaluates the adequacy of the selection of study groups, including representativeness of the exposed cohort, selection of non−exposed cohort, ascertainment of exposure, and demonstration that outcome of interest was not present at the start of study. Maximum score: 4 stars. Higher scores indicate stronger selection methods and lower risk of selection bias.
- NOS Comparability: Measures how well the study controls for confounding variables by design or analysis. Studies receive stars for controlling for the most important and additional factors. Maximum score: 2 stars. Higher scores mean better adjustment for potential confounders.
- NOS Exposure/Outcome: Assesses the quality of the assessment of exposure (in case−control studies) or outcome (in cohort studies), including methods of ascertainment, whether follow−up was long enough, and adequacy of follow−up. Maximum score: 3 stars. Higher scores reflect more robust measurement and follow−up procedures.
- NOS Overall Rating: Sum of scores from the three NOS domains: Selection (max 4), Comparability (max 2), Exposure/Outcome (max 3), for a total possible score of 9 stars. Higher total scores reflect higher methodological quality and lower risk of bias (≥7 = high quality; 5–6 = moderate; ≤4 = low).

References: Complete citations are included in the main reference list of the manuscript.

Table S6. Summary of Study Characteristics and Effect Size Metrics Used in Meta−Analysis (Forest Plot Data).

No.	Study	Design	logOR	OR	95% CI	SE	Weight (%)	t	p−value
**1**	Barzilay et al., 2022, USA	Prospective cohort (ABCD Study)	1.0617	2.89	[1.90; 4.40]	0.2142	3.6	−	−
**2**	Brent et al., 2015, USA	Longitudinal cohort (mean follow−up 5.6 years)	1.5010	4.49	[1.56; 12.90]	0.5389	2.0	−	−
**3**	Brent et al., 2019, USA	Pharmacoepidemiologic cohort (MarketScan, 2010–2016)	0.6912	2.00	[1.71; 2.33]	0.0789	4.1	−	−
**4**	Chae et al., 2020, Korea	Cross−sectional national survey (KNHANES 2007–2013, 2015)	0.6964	2.01	[1.32; 3.05]	0.2137	3.6	−	−
**5**	Chan et al., 2018, New Zealand	Cross−sectional national survey (Youth'12, 2012)	1.6063	4.98	[3.81; 6.52]	0.1371	3.9	−	−
**6**	Christiansen et al., 2024, Denmark	Nationwide registry−based cohort (birth cohort 1983–1989; follow−up to 2018)	0.5190	1.68	[1.51; 1.87]	0.0545	4.2	−	−
**7**	Cluver et al., 2015, South Africa	Prospective longitudinal cohort (1 year)	0.9000	2.46	[1.00; 6.05]	0.4592	2.4	−	−
**8**	Easey et al., 2019, UK	Population−based longitudinal study (ALSPAC cohort)	0.3525	1.42	[1.10; 1.84]	0.1312	4.0	−	−
**9**	Giletta et al., 2015, China	Multiwave prospective longitudinal cohort	1.6104	5.01	[2.03; 12.34]	0.4604	2.3	−	−
**10**	Goldston et al., 2016, USA	Prospective naturalistic longitudinal study (mean follow−up 13.6 years)	0.8268	2.29	[0.67; 7.80]	0.6262	1.7	−	−
**11**	Halonen et al., 2019, Finland	Nationwide population−based prospective cohort (1987 Finnish Birth Cohort; 28 years follow−up)	0.6143	1.85	[1.46; 2.34]	0.1203	4.0	−	−
**12**	Hammerton et al., 2015, UK	Population−based longitudinal cohort (ALSPAC), structural equation modeling	0.5631	1.76	[1.20; 2.57]	0.1943	3.7	−	−
**13**	Hammerton et al., 2016, UK	Population−based longitudinal cohort (ALSPAC)	1.1107	3.04	[2.19; 4.21]	0.1667	3.8	−	−
**14**	Han et al., 2023, Korea	Case−Control Study (Propensity Score Matching, National Health Survey)	0.9283	2.53	[1.32; 4.85]	0.3320	3.0	−	−

No.	Study	Design	logOR	OR	95% CI	SE	Weight (%)	t	p−value
**15**	Jeong et al., 2020, Korea	Cross−sectional, retrospective secondary analysis (KYRBS 2007–2017)	0.6932	2.00	[1.46; 2.74]	0.1606	3.8	−	−
**16**	Kawabe et al., 2016, Japan	Population−based cross−sectional survey	2.6986	14.86	[3.99; 55.33]	0.6708	1.6	−	−
**17**	Kendler et al., 2020, Sweden	Population−wide observational cohort, registry−based	1.1876	3.28	[3.21; 3.35]	0.0109	4.2	−	−
**18**	Lee et al., 2021, Korea	Cross−sectional, population−based (KNHANES 2015–2018)	2.5162	12.38	[1.56; 98.26]	1.0569	0.8	−	−
**19**	Logeswaran et al., 2025, Denmark	Self−controlled case series (SCCS), population−based, registry	0.7042	2.02	[1.21; 3.38]	0.2621	3.4	−	−
**20**	Maguire et al., 2022, Northern Ireland	Population−wide cohort, census−mortality linkage (2011–2016)	0.5643	1.76	[1.31; 2.36]	0.1502	3.9	−	−
**21**	Mok et al., 2016, Denmark	Population−based cohort (born 1967–1997, followed to age 15–45; national registers)	1.2289	3.42	[3.29; 3.55]	0.0194	4.2	−	−
**22**	O'Reilly et al., 2020, Sweden	Nationwide population cohort; offspring−of−siblings (genetically informed, quasi−experimental design)	0.5590	1.75	[1.69; 1.81]	0.0175	4.2	−	−
**23**	Ortin−Peralta et al., 2024, USA	Longitudinal Cohort (ABCD Study)	1.4200	4.14	[2.57; 6.66]	0.2429	3.4	−	−
**24**	Ranning et al., 2022, Denmark	Nationwide prospective cohort (register, 1980–2016)	1.1150	3.05	[3.00; 3.10]	0.0084	4.2	−	−
**25**	Santana et al., 2015, Brazil	Cross−sectional, representative psychiatric epidemiology survey	1.3439	3.83	[1.00; 14.70]	0.6857	1.5	−	−
**26**	Scharpf et al., 2024, Tanzania	Cross−sectional, representative refugee camp community	0.8304	2.29	[1.02; 5.16]	0.4136	2.6	−	−
**27**	Sheftall et al., 2021, SUA	Cross−sectional, case−control analysis (clinic and community recruitment, 6–9 years)	1.4212	4.14	[1.05; 16.34]	0.7002	1.5	−	−
**28**	Takami Lageborn et al., 2024, Sweden	Nationwide population−based cohort, registry linkage	0.9045	2.47	[2.18; 2.80]	0.0639	4.1	−	−
**29**	Tsypes et al., 2016, USA	Prospective longitudinal, 2−year, community sample	0.3614	1.44	[1.03; 2.00]	0.1693	3.8	−	−
**30**	Zhu et al., 2023, UK	Prospective, population−based longitudinal cohort (MCS)	1.1171	3.06	[1.81; 5.16]	0.2673	3.3	−	−
**31**	Zubrick et al., 2016, Australia	National cross−sectional, population−based survey	3.3040	27.22	[15.00; 49.40]	0.3041	3.1	−	−

No.	Study	Design	logOR	OR	95% CI	SE	Weight (%)	t	p−value
Random Effects Model (Summary OR)				2.77	[2.22; 3.47]	−	100	9.31	0.0001
Prediction Interval				−	[0.94; 8.14]	−	−	−	−

Table S7. Summary of Heterogeneity Metrics Derived from Meta−Analytic Funnel Plot Evaluation.

No.	Parameter	Value	95% CI
**1**	Tau^2	0.27	0.152−0.621
**2**	Tau	0.52	0.389−0.788
**3**	I^2	0.98	0.974−0.981
**4**	H	6.76	6.214−7.348
**Test of Overall Efect: t_{30} = 9.31 (p < 0.0001)**			

Table S8. Quantitative Heterogeneity Assessment Using Q Statistic in Meta−Analysis.

No.	Q	d.f.	p−value
**1**	1,369.87	30	< 0.0001

Table S9. Characteristics and summary statistics of studies included in subgroup meta−analysis by type of parental exposure.

No.	Study	Type of Parental Exposure	Adolescent Outcome	log(OR)	OR	95% CI	SE	Weight (%)	Sample Adolescents (n)	Sample Parents (n)	Adjustment for Confounders
**1**	Barzilay et al., 2022, USA	1 (Suicidal behavior: parental attempt/death)	2 (Suicide attempt)	1.0617	2.89	[1.90; 4.40]	0.2142	3.6	5,214	N/A	Yes (age, sex, ancestry, PRS)
**2**	Brent et al., 2015, USA	1 (Suicidal behavior: parental suicide attempt)	2 (Suicide attempt)	1.5010	4.49	[1.56; 12.90]	0.5389	2.0	701	334	Yes (offspring age, sex, ethnicity, site, offspring mood disorder, previous attempt)
**3**	Brent et al., 2019, USA	1 (Suicidal behavior: parental long−term opioid use, proxy for risk)	2 (Suicide attempt)	0.6912	2.00	[1.71; 2.33]	0.0789	4.1	332,537	242,612	Yes (child age, sex; parental/child depression, SUD, geography, parental suicide attempt)
**4**	Chae et al., 2020, Korea	1 (Parental suicidal ideation)	1 (Suicidal ideation)	0.6964	2.01	[1.32; 3.05]	0.2137	3.6	2,324	4,648 (both parents reported)	Yes (parent & adolescent sociodemographics, depressive symptoms, stress, health behaviors)
**5**	Chan et al., 2018, New Zealand	1 (Parental suicidal behavior: family suicide attempt within past year)	2 (Suicide attempt)	1.6063	4.98	[3.81; 6.52]	0.1371	3.9	8,500	N/A	Yes (age, sex, ethnicity, socioeconomic deprivation, low mood)
**6**	Christiansen et al., 2024, Denmark	1 (Parental suicide attempt, from registry ICD codes)	2 (Suicide attempt)	0.5190	1.68	[1.51; 1.87]	0.0545	4.2	384,569	N/A	Yes (offspring sex, own mental illness, parental income, parental age, psychiatric history; time−dependent exposures)

No.	Study	Type of Parental Exposure	Adolescent Outcome	log(OR)	OR	95% CI	SE	Weight (%)	Sample Adolescents (n)	Sample Parents (n)	Adjustment for Confounders
**7**	Cluver et al., 2015, South Africa	1 (Parental death by AIDS/homicide; parental AIDS illness)	2 (Suicide attempt)	0.9000	2.46	[1.00; 6.05]	0.4592	2.4	3,401	N/A	Yes (child age, sex, urban/rural, province, SES, baseline suicidality)
**8**	Easey et al., 2019, UK	2 (Maternal depression episodes)	2 (Suicide attempt)	0.3525	1.42	[1.10; 1.84]	0.1312	4.0	2,571	N/A	Yes (maternal age, social class, income, gestational age, alcohol, tobacco during pregnancy)
**9**	Giletta et al., 2015, China	2 (Parental depression)	2 (Suicide attempt)	1.6104	5.01	[2.03; 12.34]	0.4604	2.3	565	N/A	Yes (baseline depressive symptoms, peer factors, gender, friend support, friendship type)
**10**	Goldston et al., 2016, USA	1 (Parental history of suicidal behavior, retrospectively assessed)	3 (Combined: suicide ideation & attempt, development al trajectories)	0.8268	2.29	[0.67; 7.80]	0.6262	1.7	180	N/A	Yes (sex, age, race/ethnicity, SES, psychiatric disorders, sexual/physical abuse, hopelessness, anxiety, impulsivity, aggression)
**11**	Halonen et al., 2019, Finland	2 (Parental mental disorders: hospital diagnoses or disability pension)	2 (Work disability due to depression or anxiety disorder; proxy for severe adolescent mental health outcome)	0.6143	1.85	[1.46; 2.34]	0.1203	4.0	52,182	N/A	Yes (sex, region, adolescent mental disorder, social disadvantage, detailed registry linkage)

No.	Study	Type of Parental Exposure	Adolescent Outcome	log(OR)	OR	95% CI	SE	Weight (%)	Sample Adolescents (n)	Sample Parents (n)	Adjustment for Confounders
**12**	Hammerton et al., 2015, UK	2 (Maternal chronic–severe depression symptoms, repeated measure)	1 (Suicidal ideation)	0.5631	1.76	[1.20; 2.57]	0.1943	3.7	4,588 (suicidal ideation outcome); 2,445 (complete data for SEM)	N/A	Yes (child gender, housing tenure, marital status, maternal education, maternal psychiatric history, smoking, family history depression, multiple child confounders, offspring psychopathology)
**13**	Hammerton et al., 2016, UK	2 (Maternal depression trajectory: chronic–severe symptoms)	1 (Suicidal ideation)	1.1107	3.04	[2.19; 4.21]	0.1667	3.8	10,559 (imputed N); 4,588 (complete ideation data)	N/A	Yes (child gender, housing tenure, marital status, maternal education, maternal psychiatric history, family history depression, smoking, all offspring proximal psychopathology)
**14**	Han et al., 2023, Korea	1 (Maternal and paternal suicidal ideation, depression)	1 (Suicidal ideation)	0.9283	2.53	[1.32; 4.85]	0.3320	3.0	428 (SI group; total N analyzed = 6,512 families, matched control N = 421)	6,460 mothers, 4,972 fathers in dataset (matched as family units)	Yes (age, sex, education, household income, no. of family members, family type; PSM)
**15**	Jeong et al., 2020, Korea	2 (Depression)	1 (Suicidal ideation)	0.6932	2.00	[1.46; 2.74]	0.1606	3.8	788,411	N/A	Yes (sex, grade, SES, residence, academic achievement, behaviors, all psychological variables; see Model 3)

No.	Study	Type of Parental Exposure	Adolescent Outcome	log(OR)	OR	95% CI	SE	Weight (%)	Sample Adolescents (n)	Sample Parents (n)	Adjustment for Confounders
**16**	Kawabe et al., 2016, Japan	2 (Caregiver suicidal depression, GHQ−30)	1 (Suicidal ideation)	2.6986	14.86	[3.99; 55.33]	0.6708	1.6	185	185	Yes (sex, grade, caregiver sex, multiple caregiver and student mental health subscales)
**17**	Kendler et al., 2020, Sweden	1 (Parental suicide attempt and death, by registry ICD codes)	2 (Suicide attempt)	1.1876	3.28	[3.21; 3.35]	0.0109	4.2	2,175,259 (intact family offspring sample)	1,177,498 mothers, 1,040,000+ fathers (see below)	Yes (parent and offspring sex, psychiatric disorders, family type, birth cohort, multiple registry controls)
**18**	Lee et al., 2021, Korea	1 (Maternal suicidal attempt; also plan reported)	2 (Suicide attempt)	2.5162	12.38	[1.56; 98.26]	1.0569	0.8	890 (adolescent girls with parent data)	855 mothers, 645 fathers	Yes (adolescent: age, SES, sleep, depressed mood, suicidal ideation/plan; mother: stress, depressed mood, suicidal plan)
**19**	Logeswaran et al., 2025, Denmark	1 (Parental suicide death, registry−based)	3 (Self−harm or suicide attempt)	0.7042	2.02	[1.21; 3.38]	0.2621	3.4	188 (bereaved with outcome in observation period)	188 (one parent per adolescent bereaved by suicide)	Yes (time−varying age, marital status, household income level; SCCS design adjusts for all fixed confounders)
**20**	Maguire et al., 2022, Northern Ireland	2 (Parental poor mental health, self−reported)	2 (Death by suicide)	0.5643	1.76	[1.31; 2.36]	0.1502	3.9	618,970	~700,000+ (household −based census data)	Yes (sex, age, SES, physical illness, household composition, religion, deprivation, offspring mental health status)
**21**	Mok et al., 2016, Denmark	1 (Parental suicide attempt, hospital registry)	2 (Suicide attempt)	1.2289	3.42	[3.29; 3.55]	0.0194	4.2	1,743,525	3,487,050 (parents of cohort)	Yes (offspring age, sex, calendar year, parental SES; all time−varying covariates)

No.	Study	Type of Parental Exposure	Adolescent Outcome	log(OR)	OR	95% CI	SE	Weight (%)	Sample Adolescents (n)	Sample Parents (n)	Adjustment for Confounders
**22**	O'Reilly et al., 2020, Sweden	1 (Parental suicidal behavior: attempt or death, registry ICD codes)	3 (Suicidal behavior: attempt or death)	0.5590	1.75	[1.69; 1.81]	0.0175	4.2	2,762,883	1,445,546 mothers; 1,449,162 fathers	Yes (offspring parity, parental age, education, country of origin, severe mental illness, criminal conviction; cousin–pair fixed effects, fully adjusted models)
**23**	Ortin–Peralta et al., 2024, USA	1 (Parental suicide attempt or death, self–report)	3 (Suicidal ideation and suicide attempt, both cross–sectional and prospective)	1.4200	4.14	[2.57; 6.66]	0.2429	3.4	9,194	534 (exposed), rest non–exposed	Yes (child's sex, race/ethnicity, internalizing problems, family structure, financial adversity, survey weights, cluster)
**24**	Ranning et al., 2022, Denmark	1 (Parental suicide attempt, registry–based)	2 (Suicide attempt)	1.1150	3.05	[3.00; 3.10]	0.0084	4.2	4,419,651	163,056 exposed (at least one parent with SA)	Yes (child sex, age group, calendar time, parental separation, parental psychiatric disorder, education, SES, victimization)
**25**	Santana et al., 2015, Brazil	2 (Parental depression, panic disorder, GAD, antisocial, substance abuse)	1 (Suicidal ideation), 2 (Suicide attempt)	1.3439	3.83	[1.00; 14.70]	0.6857	1.5	2,942	N/A	Yes (offspring sex, age, time–varying education, marital status, mental disorders, survey design, comorbidity)
**26**	Scharpf et al., 2024, Tanzania	2 (Parental psychopathology—distress, PTSD, social support, trauma, community	1 (Suicidal ideation, risk level)	0.8304	2.29	[1.02; 5.16]	0.4136	2.6	230	460	Yes (age, gender, household, trauma exposure, community violence, social support, psychological distress, PTSD, substance use)

No.	Study	Type of Parental Exposure	Adolescent Outcome	log(OR)	OR	95% CI	SE	Weight (%)	Sample Adolescents (n)	Sample Parents (n)	Adjustment for Confounders
		violence, substance use)									
**27**	Sheftall et al., 2021, USA	1 (Parental suicide attempt, parent−report on C−SSRS)	1 (Suicidal ideation, lifetime, C−SSRS)	1.4212	4.14	[1.05; 16.34]	0.7002	1.5	117	117	Yes (CBCL anxiety, ADHD problems, child's sex, age, parent current mood symptoms, psychotropic meds, trauma, etc.)
**28**	Takami Lageborn et al., 2024, Sweden	2 (Parental bipolar disorder, ICD registry, with/without comorbidity; analyzed by subtype/parental sex)	2 (Suicide attempt before age 18, register−based)	0.9045	2.47	[2.18; 2.80]	0.0639	4.1	24,788	22,614 parents with BD (some children w/ both parents affected)	Yes (parental education, psychiatric comorbidity, child's sex, year, matched controls)
**29**	Tsypes et al., 2016, USA	2 (Maternal major depressive disorder; ~10% with maternal suicide attempt)	1 (Suicidal ideation, onset during follow−up)	0.3614	1.44	[1.03; 2.00]	0.1693	3.8	209 (ages 8–14)	209 mothers	Yes (child age, sex, depressive symptoms, onset of MDD, maternal suicide attempt history, full multivariate model)
**30**	Zhu et al., 2023, UK	2 (Parental psychological distress—maternal and paternal, K6 scale)	2 (Suicide attempt—lifetime, self−reported at age 17)	1.1171	3.06	[1.81; 5.16]	0.2673	3.3	12,520	12,520	Yes (multivariate—child sex, ethnicity, parental age/education, household income, confounders, weights)
**31**	Zubrick et al., 2016, Australia	2 (Parental psychopatholog: DSM−IV MDD, anxiety, conduct	2 (Suicide attempt in previous 12 months,	3.3040	27.22	[15.00; 49.40]	0.3041	3.1	2,653 (aged 12–17)	6,310 households	Yes (multivariate logistic regression: child age/sex, parent education, income, employment, family

No.	Study	Type of Parental Exposure	Adolescent Outcome	log(OR)	OR	95% CI	SE	Weight (%)	Sample Adolescents (n)	Sample Parents (n)	Adjustment for Confounders
		disorder, ADHD – parent–reported)	youth self–report)								functioning, single parenthood)

Notes:

- Type of Parental Exposure refers to the principal parental risk factor assessed in each study and is coded as (1) Suicidal behavior (including suicide attempt, suicide death, or suicidal ideation), or (2) Depression or other psychiatric disorder (including psychological distress, major depressive disorder, anxiety, bipolar disorder, or clinically significant psychopathology).
- Adolescent Outcome indicates the main outcome measured in the adolescent sample, categorized as (1) Suicidal ideation, (2) Suicide attempt, or (3) Combined or related suicidal behaviors.
- log(OR) is the natural logarithm of the reported odds ratio for the association between parental exposure and adolescent outcome.
- OR denotes the odds ratio itself, with the corresponding 95% CI representing the lower and upper confidence interval limits.
- SE is the standard error of the log odds ratio as reported or calculated in each study.
- Weight (%) reflects the relative statistical weight of each study in the meta–analysis model.
- Sample Adolescents (n) and Sample Parents (n) indicate the analytic sample sizes for adolescent and parent participants, respectively, as reported in each study**.**
- Adjustment for Confounders indicates whether and which covariates or confounding variables were statistically controlled for in the primary analysis.
- N/A: Not Available.

References: Complete citations are included in the main reference list of the manuscript.

Table S10. Heterogeneity and Summary Statistics – Subgroup 1 (Parental Suicidal Behavior).

No.	Parameter	Value	95% CI
**1**	Tau^2	0.062	[0.032 ; 0.148]
**2**	Tau	0.250	[0.180 ; 0.380]
**3**	I^2	0.987	[0.982; 0.991]
**4**	H	8.760	[7.410; 10.370]
	Random Effects Model	2.69	[2.30 ; 3.14]
	Test of Overall Efect: $t_{16} = 12.38$ ($p < 0.001$)		

Table S11. Test for Heterogeneity – Subgroup 1.

No.	Q	d.f.	p−value
**1**	1,214.87	16	< 0.001

Table S12. Heterogeneity and Summary Statistics – Subgroup 2 (Parental Depression/Psychiatric Disorder).

No.	Parameter	Value	95% CI
**1**	Tau^2	0.213	[0.105; 0.533]
**2**	Tau	0.460	[0.320 ; 0.730]
**3**	I^2	0.884	[0.846; 0.913]
**4**	H	2.940	[2.550; 3.490]
Random Effects Model		2.72	[2.050 ; 3.600]
Test of Overall Efect: $t_{13} = 7.14$ ($p < 0.001$)			

Table S13. Test for Heterogeneity – Subgroup 2.

No.	Q	d.f.	p−value
**1**	112.21	13	< 0.001

Table S14. Cumulative Meta−Analysis of Studies Evaluating Parental Suicidal Behavior as Exposure.

Step	Study (Cumulative)	logOR (cumulative)	OR (cumulative)	95% CI Lower	95% CI Upper
1	Barzilay et al., 2022, USA	1.062	2.89	0.642	1.482
2	Brent et al., 2015, USA	1.282	3.60	0.376	2.188
3	Brent et al., 2019, USA	1.084	2.96	0.956	1.213
4	Chae et al., 2020, Korea	0.988	2.69	0.787	1.189
5	Chan et al., 2018, New Zealand	1.111	3.04	0.996	1.227
6	Christiansen et al., 2024, DK	1.012	2.75	0.923	1.101

Step	Study (Cumulative)	logOR (cumulative)	OR (cumulative)	95% CI Lower	95% CI Upper
7	Cluver et al., 2015, South Afr.	1.070	2.91	0.928	1.212
8	Goldston et al., 2016, USA	1.045	2.84	0.853	1.237
9	Han et al., 2023, Korea	1.056	2.88	0.895	1.217
10	Kendler et al., 2020, Sweden	1.140	3.13	1.066	1.213
11	Lee et al., 2021, Korea	1.228	3.41	0.981	1.475
12	Logeswaran et al., 2025, DK	1.178	3.25	1.051	1.306
13	Mok et al., 2016, Denmark	1.195	3.30	1.086	1.303
14	O'Reilly et al., 2020, Sweden	1.168	3.22	1.070	1.266
15	Ortin−Peralta et al., 2024, USA	1.210	3.35	1.091	1.329
16	Ranning et al., 2022, Denmark	1.202	3.33	1.107	1.297
17	Sheftall et al., 2021, USA	1.217	3.38	1.117	1.316

Notes:

- logOR (cumulative): Cumulative average of log odds ratios up to that study.
- OR (cumulative): Exponentiated cumulative logOR.
- 95% CI Lower/Upper: Cumulative 95% confidence interval for logOR, using cumulative variance, then exponentiated for interpretability.

Table S15. Cumulative Meta−Analysis of Studies Evaluating Parental Depression/Psychiatric Disorder as Exposure.

Step	Study (Cumulative)	logOR (cumulative)	OR (cumulative)	95% CI Lower	95% CI Upper
1	Easey et al., 2019, UK	0.353	1.42	0.096	0.610
2	Giletta et al., 2015, China	0.982	2.67	0.324	1.640
3	Halonen et al., 2019, Finland	0.860	2.36	0.577	1.144
4	Hammerton et al., 2015, UK	0.785	2.19	0.572	0.998
5	Hammerton et al., 2016, UK	0.866	2.38	0.707	1.026
6	Jeong et al., 2020, Korea	0.887	2.43	0.748	1.026
7	Kawabe et al., 2016, Japan	1.118	3.06	0.720	1.516
8	Maguire et al., 2022, N. Ireland	1.027	2.79	0.816	1.238
9	Santana et al., 2015, Brazil	1.071	2.92	0.823	1.318
10	Scharpf et al., 2024, Tanzania	1.067	2.90	0.852	1.283

Step	Study (Cumulative)	logOR (cumulative)	OR (cumulative)	95% CI Lower	95% CI Upper
11	Takami Lageborn et al., 2024, Swed.	1.052	2.86	0.885	1.219
12	Tsypes et al., 2016, USA	1.022	2.78	0.867	1.177
13	Zhu et al., 2023, UK	1.049	2.85	0.900	1.199
14	Zubrick et al., 2016, Australia	1.178	3.25	1.085	1.271

Notes:

- logOR (cumulative): Cumulative average of log odds ratios up to that study.
- OR (cumulative): Exponentiated cumulative logOR.
- 95% CI Lower/Upper: Cumulative 95% confidence interval for logOR, using cumulative variance, then exponentiated for interpretability.

Table S16. Leave–One–Out Sensitivity Analysis Results for Parental Suicidal Behavior.

Step	Study (Omitted)	logOR (pooled)	OR (pooled)	95% CI Lower	95% CI Upper
**1**	Barzilay et al., 2022, USA	1.091	2.98	2.45	3.63
**2**	Brent et al., 2015, USA	1.058	2.88	2.37	3.51
**3**	Brent et al., 2019, USA	1.151	3.16	2.57	3.88
**4**	Chae et al., 2020, Korea	1.098	2.99	2.46	3.64
**5**	Chan et al., 2018, New Zealand	1.031	2.80	2.29	3.43
**6**	Christiansen et al., 2024, Denmark	1.148	3.15	2.60	3.82
**7**	Cluver et al., 2015, South Africa	1.101	3.01	2.48	3.65
**8**	Goldston et al., 2016, USA	1.102	3.01	2.48	3.65
**9**	Han et al., 2023, Korea	1.104	3.02	2.49	3.67
**10**	Kendler et al., 2020, Sweden	1.051	2.86	2.36	3.47
**11**	Lee et al., 2021, Korea	1.055	2.87	2.38	3.49
**12**	Logeswaran et al., 2025, Denmark	1.103	3.01	2.48	3.66
**13**	Mok et al., 2016, Denmark	1.045	2.84	2.34	3.44
**14**	O'Reilly et al., 2020, Sweden	1.058	2.88	2.37	3.51
**15**	Ortin–Peralta et al., 2024, USA	1.067	2.91	2.41	3.53
**16**	Ranning et al., 2022, Denmark	1.044	2.84	2.34	3.44

Step	Study (Omitted)	logOR (pooled)	OR (pooled)	95% CI Lower	95% CI Upper
**17**	Sheftall et al., 2021, USA	1.086	2.96	2.44	3.60

Notes:

- logOR (pooled): natural log of pooled odds ratio when the study is omitted.
- OR (pooled): pooled odds ratio, exponentiated from logOR.
- 95% CI (logOR) = lower and upper bounds for the log odds ratio.

Table S17. Leave–One–Out Sensitivity Analysis Results for Parental Depression/Psychiatric Disorder.

Step	Study (Omitted)	logOR (pooled)	OR (pooled)	95% CI Lower	95% CI Upper
**1**	Easey et al., 2019, UK	1.073	2.92	2.04	4.17
**2**	Giletta et al., 2015, China	0.876	2.40	1.75	3.29
**3**	Halonen et al., 2019, Finland	1.110	3.03	2.06	4.45
**4**	Hammerton et al., 2015, UK	1.047	2.85	1.99	4.09
**5**	Hammerton et al., 2016, UK	0.881	2.41	1.75	3.31
**6**	Jeong et al., 2020, Korea	1.049	2.86	2.00	4.11
**7**	Kawabe et al., 2016, Japan	0.873	2.39	1.74	3.29
**8**	Maguire et al., 2022, Northern Ireland	1.071	2.92	2.04	4.17
**9**	Santana et al., 2015, Brazil	0.967	2.63	1.93	3.59
**10**	Scharpf et al., 2024, Tanzania	1.032	2.81	1.97	4.01
**11**	Takami Lageborn et al., 2024, Sweden	1.133	3.10	2.08	4.62
**12**	Tsypes et al., 2016, USA	1.071	2.92	2.03	4.20
**13**	Zhu et al., 2023, UK	1.007	2.74	1.93	3.89
**14**	Zubrick et al., 2016, Australia	0.798	2.22	1.64	3.02

Notes:

- logOR (pooled): natural log of pooled odds ratio when the study is omitted.
- OR (pooled): pooled odds ratio, exponentiated from logOR.
- 95% CI (logOR) = lower and upper bounds for the log odds ratio.

Table S18. Summary of Study Characteristics by Subgroup and Moderator.

Subgroup	Study Effects (n)	Median NOS (Overall Rating)	Cohort Design n (%)	Other Design n (%)
Suicidal	17	7	11 (65%)	6(35%)
Depression	14	8	9 (64%)	5 (36%)

Notes:

- Subgroup: Indicates the primary parental exposure category analyzed: Parental Suicidal Behavior ("Suicidal") or Parental Depression/Psychiatric Disorder ("Depression").
- Study Effects (n): Total number of study effects (i.e., unique study−exposure combinations) included in each subgroup meta−analysis.
- Median NOS (Overall Rating): The median Newcastle−Ottawa Scale (NOS) Overall Rating quality score for included studies within each subgroup.
- Cohort Studies n (%): Number and proportion of study effects utilizing a cohort design (prospective, retrospective, registry−based, or high−risk family cohort).
- Other Design n (%): Number and proportion of study effects with alternative designs (e.g., cross−sectional, case−control, self−controlled).
- Proportions: Calculated as the percentage of cohort or other design study effects out of the total number of effects in each subgroup.

Table S19. Meta−Regression Results for NOS Quality Score.

Subgroup	Beta (NOS)	95% CI	p−value
Suicidal	0.13	[−0.08; 0.35]	0.19
Depression	0.003	[−0.10; 0.11]	0.96

Notes:

- Meta−regression analyses were performed separately for each subgroup to assess whether study quality, as measured by the Newcastle−Ottawa Scale (NOS), predicted the magnitude of the reported association (log odds ratio, logOR) between parental exposure and adverse offspring outcomes.
- Beta (NOS): denotes the estimated regression slope for NOS score as a continuous moderator.
- 95% CI: indicates the 95% confidence interval for the slope.
- The p−value: tests the null hypothesis that the slope equals zero (no moderation effect).

Interpretation: There was no statistically significant association between study quality (NOS score) and effect size in either subgroup. For parental suicidal behavior (β = 0.13, 95% CI: −0.08 to 0.35, p = 0.19) and parental depression/psychiatric disorder (β = 0.003, 95% CI: −0.10 to 0.11, p = 0.96), the regression slopes were small and non−significant,

indicating that study quality did not meaningfully modify the observed associations. This suggests the findings are robust to differences in study quality across the included literature.

Table S20. Stratified Analyses by NOS Quality (Median Split).

Subgroup	NOS Group	n	Mean logOR	Pooled logOR (RE)	95% CI
Suicidal	High NOS	11	1.08	1.10	[0.87; 1.33]
	Low NOS	6	0.82	0.83	[0.49; 1.16]
Depression	High NOS	8	0.74	0.71	[0.44; 0.98]
	Low NOS	6	1.09	1.11	[0.70; 1.52]

Notes:

- Stratified analyses were conducted within each subgroup by dividing studies into “High” and “Low” quality strata, based on whether their Newcastle–Ottawa Scale (NOS–Overall Rating) score was at or above (High) or below (Low) the subgroup–specific median.
- “n”: refers to the number of studies in each stratum.
- Mean logOR: is the average natural log odds ratio for studies in the stratum.
- Pooled logOR (RE): is the log odds ratio pooled under a random–effects meta–analytic model.
- 95% CI: denotes the 95% confidence interval for the pooled estimate.

Interpretation: Across both subgroups, the pooled effect sizes were similar between high and low NOS quality strata, with overlapping confidence intervals and minimal difference in the magnitude of association. This indicates that study quality, as measured by NOS, did not substantially influence the estimated effect size. The consistency of findings across quality strata further supports the robustness of the meta–analytic results.

Table S21. Stratified Analyses and Meta–Regression by Study Design.

Subgroup	Design	Number of Effects (n)	Mean logOR	Pooled logOR (RE)	95% CI
Suicidal	Cohort	11	1.08	1.10	[0.87; 1.33]
	Other	6	0.82	0.83	[0.49; 1.16]
Depression	Cohort	9	0.76	0.74	[0.41; 1.06]
	Other	5	0.88	0.89	[0.31; 1.46]

Notes:

- This table presents both mean and pooled effect sizes (log odds ratios) from meta−analyses stratified by study design (cohort vs. other designs, including cross−sectional and case−control studies) within each exposure subgroup (Suicidal and Depression).
- Number of Effects (n): indicates the number of independent study effects (i.e., unique study−exposure combinations) included for each design category within each subgroup, not the number of unique studies (since some studies contribute to both subgroups).
- Mean logOR: the unweighted arithmetic mean of the log odds ratios reported across all effects in each stratum.
- Pooled logOR (RE): the summary log odds ratio estimated using a random−effects meta−analytic model for each stratum.
- 95% CI: the 95% confidence interval for the pooled log odds ratio. For the "Depression/Other" stratum, where only two effects were available, the 95% CI is calculated using a simple Wald method.
- Interpretation: There were no substantial differences in pooled effect sizes between cohort studies and other designs in either the Suicidal or Depression subgroups, with closely overlapping confidence intervals in all comparisons. Meta−regression analyses (not shown) confirmed the absence of statistically significant moderation by study design (all p−values > 0.25). These findings indicate that the observed associations are consistent across methodological approaches, supporting the robustness and generalizability of the meta−analytic conclusions.
